# Deoxythymidylate Kinase as a Promising Marker for Predicting Prognosis and Immune Cell Infiltration of Pan-cancer

**DOI:** 10.3389/fmolb.2022.887059

**Published:** 2022-07-12

**Authors:** Tianfeng Lan, Yachao Wang, Jinxin Miao, Haoran Guo, Zheng Wang, Jianyao Wang, Chunyang Zhang, Panpan Yang, Zhongxian Zhang, Louisa Chard Dunmall, Yaohe Wang

**Affiliations:** ^1^ State Key Laboratory of Esophageal Cancer Prevention and Treatment, Sino-British Research Center for Molecular Oncology, National Center for the International Research in Cell and Gene Therapy, School of Basic Sciences, Academy of Medical Sciences, Zhengzhou University, Zhengzhou, China; ^2^ Academy of Chinese Medicine Science, Henan University of Chinese Medicine, Zhengzhou, China; ^3^ Department of Surgical Sciences, The First Affiliated Hospital of Zhengzhou University, Zhengzhou, China; ^4^ Centre for Cancer Biomarkers and Biotherapeutics, Barts Cancer Institute, Queen Mary University of London, London, United Kingdom

**Keywords:** deoxythymidylate kinase, expression profile, prognosis, immune infiltration, pancancer

## Abstract

**Background:** Deoxythymidylate kinase (DTYMK) serves as a pyrimidine metabolic rate-limiting enzyme that catalyzes deoxythymidine monophosphate (dTMP) to generate deoxythymidine diphosphate (dTDP). It remains unclear whether DTYMK expression has the potential to predict outcome and immune cell infiltration in cancers.

**Methods:** DTYMK expression profile was analyzed using Oncomine, TIMER, GEPIA and UALCAN databases. The influence of DTYMK on immune infiltration was examined using TIMER and TISIDB databases. DTYMK interactive gene hub and co-expressing genes were obtained and analyzed by STRING and Linkedomics, respectively. The relationship between DTYMK expression and patient prognosis was validated using GEPIA, Kaplan-Meier plotter, and PrognoScan databases. The functions of DTYMK in cancer cells were also biologically validated *in vitro*.

**Results:** DTYMK expression was elevated in tumor tissues compared with their control counterparts. DTYMK expression varied in different stages and discriminatorily distributed in different immune and molecular subtypes. Higher expression of DTYMK predicted worse outcome in several cancer types such as liver hepatocellular carcinoma (LIHC) and lung adenocarcinoma (LUAD). High DTYMK expression was positively or negatively correlated with immune cell infiltration, including B cell, CD8^+^ cell, CD4^+^ T cell, macrophage, neutrophil and dendritic cell, depending on the type of cancers. Additionally, DTYMK co-expressing genes participated in pyrimidine metabolism as well as in T helper cell differentiation in LIHC and LUAD. *In vitro*, knockdown of DTYMK suppressed cell migration of liver and lung cancer cells.

**Conclusion:** DTYMK might be taken as an useful prognostic and immunological marker in cancers and further investigation is warrented.

## Introduction

Metabolic reprogramming is a major hallmark of cancer (Hanahan and Weinberg, 2011). In addition to the energy requirement for cancer cell expansion, cellular building blocks including nucleotides, amino acids and lipids are also crucially in need during tumor expansion (Vander Heiden and DeBerardinis, 2017). Overactivation of signature genes involved in the biosynthesis of cellular components contributes to the progression of malignant tumors (Vander Heiden and DeBerardinis, 2017). Pyrimidine metabolism (PyM) is responsible for the generation of ribonucleotide phosphates and deoxyribonucleoside triphosphates (dNTPs) (Evans and Guy, 2004). Three complex pathways including *de novo* nucleotide synthesis, nucleoside salvage and catalytic degradation of pyrimidines are involved in PyM. Various PyM-associated genes within the pathways are involved in the synthesis, degradation, salvage, interconversion and transport of pyrimidines and their intermediates (Siddiqui and Ceppi, 2020).

The presence of cancer driven mutations like p53^MUT^ and KRAS^MUT^ or overactivation of v-myc avian myelocytomatosis viral oncogene homolog (MYC) and mechanistic target of rapamycin kinase (mTOR) in cancer cells activates PyM-associated genes (Pugacheva et al., 2002; Liu et al., 2008; Ben-Sahra et al., 2013; Santana-Codina et al., 2018). Robust activation of PyM-associated genes not only supplies adequate dNTPs for cancer cell survival, but also contributes to chemoresistance, stemness and epithelial-mesenchymal transition (EMT) (Dongre and Weinberg, 2019). The role of PyM is not limited to the support of cell proliferation. The activities of PyM anabolic enzymes are higher in leukemic cells than normal leukocytes (Shiotani et al., 1989) and inhibition of PyM anabolism in AML results in blast cell maturation (Christian et al., 2019). In breast and liver cancer, PyM catabolism is essential for maintaining the mesenchymal-like state of cancer cells (Shaul et al., 2014; Zhu et al., 2018). Choi et al. (Choi and Na, 2018) examined the association between genetic changes within the metabolic landscape and cancer progression across 29 cancers. Their results demonstrated that PyM expression strongly correlated with tumor mutation burden (TMB) and poor prognosis in several cancers (Choi and Na, 2018).

Deoxythymidylate kinase (DTYMK), also named thymidylate kinase (TMPK; EC 2.7.4.9; reaction: ATP + dTMP = ADP + dTDP), is an essential PyM-associated gene. The enzyme comprises three important domains: the ligand-induced degradation (LID) domain, nucleoside monophosphate (NMP) binding site and the CORE domain (Whittingham et al., 2010). DTYMK serves as a pyrimidine metabolic rate-limiting enzyme that catalyzes production of deoxythymidine diphosphate (dTDP) from deoxythymidine monophosphate (dTMP). In the presence of ATP and Mg2+, DTYMK/TMPK catalyzes the phosphorylation of dTMP to generate dTDP. Nucleosidediphosphate kinase (NDK) adds another phosphate group to dTDP and produces deoxythymidine 5′-triphosphate (dTTP). In the *de novo* pathway, thymidylate synthase (TS) catalyzes the synthesis of dTMP using dUMP, while in salvage pathway, dTMP comes from thymidine kinase (TK) using deoxythymidine (dT) as substrate (Cui et al., 2013). The link between DTYMK and human diseases has been documented in several studies. Lam et al. (Lam et al., 2019) demonstrated that compound heterozygous mutations in DTYMK may be involved in mitochondrial DNA depletion syndrome (MDDS). Enhanced DTYMK expression was characterized in TP53-mutant lung cancer patients and associated with poor prognosis (Wang et al., 2020). Disruption of DTYMK expression using small hairpin RNA (shRNA) resulted in cell death in colon cancer cells regardless of p53 status (Hu and Chang, 2008). DTYMK deletion reduced the dTDP pool and markedly suppressed lung cancer cell growth under serine/threonine kinase 11 (LKB1)-deficient conditions (Liu et al., 2013). Additionally, knockdown of DTYMK expression suppressed cell proliferation while overexpression of DTYMK promote that in hepatocellular carcinoma (HCC) cell line (Zhou et al., 2021). These studies suggest DTYMK as a potential molecular target for the treatment of malignant tumors and other diseases.

It has been well-established that the complex interplay between tumor cells and the tumor microenvironment (TME) drives cancer development (Hinshaw and Shevde, 2019). Infiltrating immune cells are a crucial component of the TME (Hinshaw and Shevde, 2019). The correlation between immune cell infiltration, such as CD4^+^ and CD8^+^ T cells, dendritic cells (DCs) and macrophages with patient prognosis has been noted in a number of studies (Ino et al., 2013; Hu et al., 2020). The presence of innate immune cells including myeloid-derived suppressor cells (MDSCs) and tumor-associated macrophages (TAMs) have been correlated with poor outcome and tumor progression (Gajewski et al., 2013). TAMs have been characterized by excessive production of *de novo* synthesized pyrimidines and mediate chemoresistance in pancreatic cancer (Halbrook et al., 2019). The role of DTYMK on immune infiltration into cancers remains unexplored.

In the present study, a comprehensive analysis of DTYMK expression and its relationship with patient prognosis was performed by taking the advantage of several online databases. Notably, we shed light on the positive correlation between DTYMK expression and immune cell infiltration in several cancers, such as brain lower grade glioma (LGG) and LIHC, but negative correlation in others like LUAD and stomach adenocarcinoma (STAD). Through pan-cancer analysis, the prognostic and immune cell infiltration value of DTYMK expression in human cancers, especially in LIHC and LUAD were proven. Our results strengthen the viewpoint that DTYMK is a promising therapeutic target for cancers.

## Materials and Methods

The detailed workflow for this study is shown in Supplementary Figure S1.

### Oncomine Database

Oncomine database (https://software.oncomine.com/resource/login.html) is useful tool for gene-wide expression analyses (Rhodes et al., 2004). Oncomine was used to summarize DTYMK expression in tumor tissues and their normal counterparts across human cancers. Parameters for disease screens included: *p* Value less than 0.001, fold change over 1.5, Gene rank: Top 10%, data type mRNA.

### GEPIA (Gene Expression Profiling Interactive Analysis) Database

GEPIA (http://gepia.cancer-pku.cn/index.html) contains RNA sequencing expression data of tumors and normal samples from the TCGA and the GTEx projects (Tang et al., 2017). The comparison of DTYMK expression between tumors and normal samples and in different tumor stages was performed using this platform. The effects of DTYMK expression on prognosis (overall survival and disease-free survival) were also studied.

### cBioPortal Database

Genetic alterations of DTYMK in cancers were analyzed using cBioPortal database (http://www.cbioportal.org/), which comprises molecular profiles and clinical attributes from The Cancer Genome Atlas (Cerami et al., 2012; Gao et al., 2013).

### UALCAN Database

Clinical Proteomic Tumor Analysis Consortium (CPTAC) Confirmatory/Discovery dataset in UALCAN database (http://ualcan.path.uab.edu/) was useful for analysis DTYMK protein expression (Chandrashekar et al., 2017; Chen et al., 2019).

### TIMER: Tumor IMmune Estimation Resource Database

TIMER web server (https://cistrome.shinyapps.io/timer/) is a comprehensive resource for systematical analysis of immune infiltrates across diverse cancer types (Li et al., 2016; Li et al., 2017). The abundances of six immune infiltrates (B cells, CD4^+^ T cells, CD8^+^ T cells, Neutrophils, Macrophages and Dendritic cells) were estimated using the TIMER algorithm. The analysis of DTYMK expression in tumors and control samples as well as its relationship with immune infiltration was carried out using TIMER.

### Kaplan-Meier Plotter

The Kaplan-Meier plotter (http://kmplot.com/analysis/) can be harnessed for assessing the impact of genes on patient survival in 21 cancer types (Nagy et al., 2021). The effect of DTYMK on survival (overall survival and disease-free survival) was evaluated in all cancer types.

### Prediction Performance of DTYMK Expression in Prognosis

Based on TCGA data, the univariate Cox regression analysis and multivariate Cox regression analysis to further clarify the relationship between DTYMK expression and the prognosis in LIHC and LUAD. The time-dependent receiver operating characteristic (ROC) analysis was used to compare the predictive accuracy of DTYMK expression in LIHC and LUAD.

### PrognoScan Database

PrognoScan (http://www.prognoscan.org/) was used to evaluate the relationship between gene expression and patient prognosis across a large collection of publicly available cancer microarray datasets (Mizuno et al., 2009). We searched the item “DTYMK” and screened datasets which showed the significant relationship between DTYMK expression and patient survival. Based on the analysis results, a web tool, Sangerbox (http://sangerbox.com/Tool) was then employed to draw a forest plot.

### TISIDB Database

TISIDB (http://cis.hku.hk/TISIDB/index.php) integrates genomics, transcriptomics and clinical data of 30 cancer types from The Cancer Genome Atlas (TCGA) as well as other data for tumor and immune system interaction (Ru et al., 2019). TISIB was used to evaluate DTYMK expression in different immune subtypes and molecular subtypes of tumors. We also used TISIB to explore the relationship between DTYMK and immune infiltration, MHC expression, immune inhibitors, immunostimulators, chemokines and chemokine receptors.

### GeneMANIA

GeneMANIA (http://genemania.org/) helps to determine related genes of input genes with a large set of functional association data (Warde-Farley et al., 2010). We used GeneMANIA to define the protein-protein interaction network of DTYMK.

### STRING

The STRING database (https://www.string-db.org/) comprises and predicts physical and functional associations between proteins (Szklarczyk et al., 2021). Fifty interactive proteins of DTYMK were predicted using STRING. Key parameters included: minimum required interaction score (medium confidence 0.400), max number of interactors to show (no more than 50 interactors). Kyoto encyclopedia of genes and genomes (KEGG) enrichment and gene ontology (GO) analysis of these fifty interactive genes were performed using The Database for Annotation, Visualization and Integrated Discovery (DAVID, https://david.ncifcrf.gov/home.jsp) (Huang da et al., 2009a; Huang da et al., 2009b).

### LinkedOmics Database

The LinkedOmics database (http://www.linkedomics.org/) encompasses multi-omics data and clinical information from TCGA (Vasaikar et al., 2018). This platform allows researchers to analyze cancer multi-omics data within and across tumor types. Here, the LinkFinder module was employed to generate a volcano plot and heatmap plot for gene correlations with DTYMK in LIHC and LUAD. The LinkInterpreter module was used for Gene Set Enrichment Analysis (GSEA) for correlated genes in LIHC and LUAD.

### Drug Bank

Drug bank (https://go.drugbank.com/) combines detailed drug data with comprehensive drug target information (Wishart et al., 2006; Wishart et al., 2008; Knox et al., 2011; Law et al., 2014; Wishart et al., 2018). The pharmaco-transcriptomics module was used to search those pharmaceutical compounds that significantly influenced DTYMK expression.

### Cell Culture

Lung cancer cell lines including 95C and 95D were obtained from the Cell Bank of the Chinese Academy of Sciences (Shanghai, China). A549, H1299, MIA Paca-2 and HepG2 cells were provided by American Type Culture Collection (ATCC, Manassas, VA, United States). 95C, 95D and H1299 were cultured with Roswell Park Memorial Institute (RPMI)-1640 medium (Thermo Fisher Scientific, Waltham, MA, United States) supplemented with 10% fetal bovine serum (FBS, PAN Biotech, Aidenbach, Germany). A549, MIA Paca-2 and HepG2 were cultured with Dulbeccos Modified Eagles Media (DMEM) medium (high glucose, Thermo Fisher Scientific) supplemented with 10% FBS and penicillin/streptomycin (100 U/mL, North China Pharmaceutical Company, Shijiazhuang, China). All cell lines were cultured under 37°C and 5% CO_2_ conditions.

### shRNAs Transfection

Two shRNAs targeting DTYMK (NM_012145) were designed using BLOCK-iT™ RNAi Designer (https://rnaidesigner.thermofisher.com/rnaiexpress/sort.do) and synthesized by Sangon Biotech (Shanghai, China). Paired primers of DTYMK shRNAs or scrambled shRNAs were annealed (heating at 90°C for 15 min and cooling at room temperature for 2 h) and then ligated into the EcoR I/Age I linearized pshRNA-EGFP-P2A-Puro plasmid (designated as pshDTYMK-EGFP-P2A-Puro and pshNC-EGFP-P2A-Puro). Sequences for shDTYMK01: forward primer: CCG​GGG​GAA​CAA​GTG​CCG​TTA​ATT​ATT​CAA​GAG​ATA​ATT​AAC​GGC​ACT​TGT​TCC​CTT​TTT​G; Reverse primer: AATTCAAAAA GGG​AAC​AAG​TGC​CGT​TAA​TTA​TCT​CTT​GAA​TAA​TTA​ACG​GCA​CTT​GTT​CCC; Sequences for shDTYMK02: Forward primer: CCG​GGC​AAA​TCG​CTG​GGA​ACA​AGT​G TTC​AAG​AGA​CAC​TTG​TTC​CCA​GCG​ATT​TGC​TTT​TTG; Reverse primer: AAT​TCA​AAA​AGC​AAA​TCG​CTG​GGA​ACA​AGT​GTC​TCT​TGA​ACA​CTT​GTT​CCC​AGC​GAT​TTG​C.

After cloning and validation by sequencing, pshDTYMK-EGFP-P2A-Puro and pshNC-EGFP-P2A-Puro were used to generate lentiviruses with packaging vectors including psPAX2 and pMD2.G and the transfection reagent Polyethylenimine (PEI, Polysciences, Warrington, PA, United States) as detailed in a previous study (Dey et al., 2017).

To establish stable knockdown cell lines, HepG2 and H1299 cells were transfected with Lenti-shDTYMK and ctrl viruses (10 MOI) and selected using puromycin (2-4 μg/ml) for 2 weeks. Western blot was employed to validate knockdown efficiencies as detailed below.

### Plate Colony Formation Assay

After DTYMK knockdown, HepG2 and H1299 cells were seeded on 6-well plates at 400 cells/well and allowed to grow for 10 days. When more than 50 cells were observed in each colony, culture medium was removed and washed twice with PBS. Tumor cells were fixed using absolute methanol for 15 min and stained with 0.1% crystal violet. After washing with distilled water three times, photos were obtained using microscopy (Olympus, Tokyo, Japan).

### Wound Healing Assay

HepG2 and H1299 cells were seeded on 6-well plates at 5×10^5^ cells/well and allowed to grow for 24 h. When cell confluence reached 70%, culture medium was removed and cells were washed twice with PBS. Cells were scratched using a sterilized pipette tip and then cultured with DMEM (HepG2) or 1640 (H1299) without FBS. Wound healing was examined and photos were captured under a light microscope after 48 h. Wound healing areas were calculated using ImageJ (version 1.53, NIH, Bethesda, MA, United States). Wound closure was calculated using the algorithm: wound closure % = [(A_0h_-A_48h_)/A_0h_] × 100%.

### Transwell Assay

HepG2 and H1299 cells were seeded on the upper chambers of Transwells (Corning, Corning, NY, United States) at 5 × 10^4^ cells/well in medium without FBS. Chambers were then immersed into medium containing 10% FBS and tumor cells were allowed to migrate for 24 h. Migratory cells were fixed with 4% formaldehyde, penetrated by absolute methanol and stained using 0.1% crystal violet. Migratory cells were observed under the microscope and counted in four different fields.

### Western Blot

Western blot analysis of DTYMK expression was performed according to a previous study (Miao et al., 2020). Preparation of protein from 95C, 95D, A549, MIA Paca-2, HepG2 and H1299 cells was performed using the Mammalian Protein Extraction Kit (CWBIO company, Beijing, China) supplemented with proteinase inhibitor (dilution 1:99). Protein concentrations were determined using the Bicinchoninic assay (BCA) method (CWBIO company). Proteins were separated by 10% SDS-PAGE gels and then transblotted into polyvinylidene fluoride (PVDF) membrane. After washing with TBS-Tween and blocking using 5% skim milk powder, the PVDF membrane was incubated with the DTYMK polyclonal antibody (1:1000, #15360-1-AP, Proteintech, Rosemont, IL, United States). *β*-actin (1:1000, Proteintech) was used as an internal loading control. After washing, the membrane was incubated with horseradish peroxidase (HRP)-conjugated goat anti-rabbit IgG (1:5000, Proteintech) for 1 hour. Electrochemiluminescence (ECL) reagent was used to visualize protein bands and ImageJ software (version 1.53, NIH) used to measure the mean gray-value of protein bands.

### Statistical Analysis

Data were presented as Mean ± SD. DTYMK expression comparison among different cancers in TIMER was calculated using the Wilcoxon rank-sum test. Patient survival comparisons between individuals with high and low expression of DTYMK was carried out using GEPIA, Kaplan-Meier plotter and PrognoScan. DTYMK co-expressing genes were obtained from LinkedOmics and screened using Spearman’s correlation. For *in vitro* experiments, the difference among groups was calculated by One-way ANOVA and multiple comparisons was performed using the Tukey post-test. *p* values of less than 0.05 were considered as statistically significant.

## Results

### DTYMK Expression is Upregulated in a Wide Range of Cancers

**FIGURE 1:**
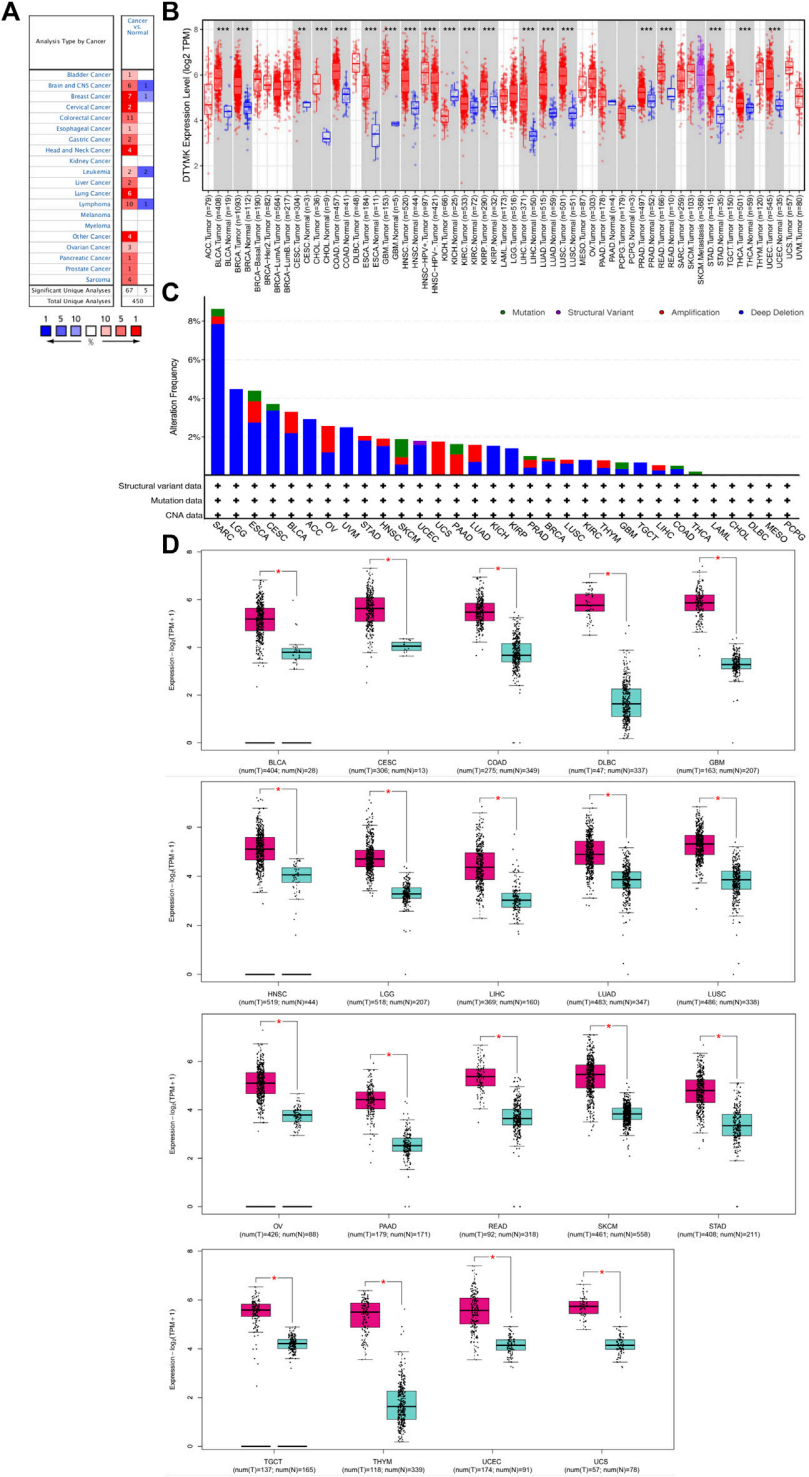
DTYMK expression is upregulated in most types of cancers compared with controls. **(A)** Exploration of DTYMK mRNA expression in human cancers as determined using the Oncomine database. Numbers in red boxes indicates studies that showed increased DTYMK expression in tumors; numbers in blue indicates studies that showed decreased DTYMK expression. **(B)** Comparison of DTYMK mRNA expression in human cancers using the TIMER database. **(C)** Profile of DTYMK expression variation and mutation in cancers using the cBioPortal database. **(D)** Comparison of DTYMK mRNA expression between tumor tissues and normal tissues using GEPIA database. *p < 0.05, **p < 0.01, ***p < 0.001.

**FIGURE 2:**
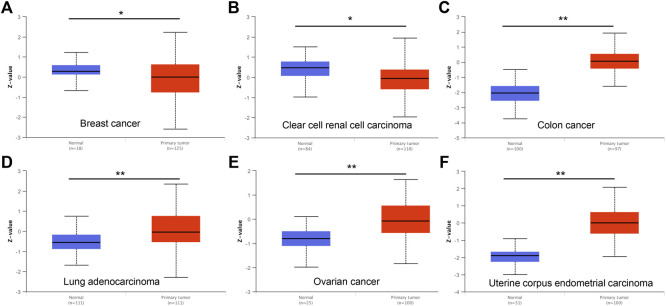
DTYMK protein expression is changed in cancers examined using the UALCAN database. DTYMK protein expression is significantly reduced in breast cancer **(A)**, clear cell renal cell carcinoma **(B)** but augmented in colon cancer **(C)**, lung adenocarcinoma **(D)**, ovarian cancer **(E)** and uterine corpus endometrial carcinoma **(F)**. *p < 0.05.

To characterize DTYMK expression in various of cancer types, DTYMK transcription was analysed using the Oncomine platform (Figure 1A). Colorectal cancer, lymphoma, and breast cancer were the top three malignancies showing increased DTYMK expression in this platform (Supplementary Table S1). DTYMK expression was then compared between tumor tissues and normal controls by TIMER using data derived from the TCGA database. Significant augmentation of DTYMK expression was observed in 19 cancer types, including bladder urothelial carcinoma (BLCA), breast invasive carcinoma (BRCA), and cervical squamous cell carcinoma (CESC) etc. (Figure 1B). Genetic alteration of DTYMK in cancers included gene mutations, structural variants, amplification and deep deletion (homozygous deletion). Although deep deletion of DTYMK was common to most cancers, the frequency was low. For instance, there was 7.84% (20/255) in sarcoma (Figure 1C). Gene amplification of DTYMK was more common in cancers such as ovarian serous cystadenocarcinoma (OV), uterine carcinosarcoma (UCS), pancreatic adenocarcinoma (PAAD), LUAD, thymoma (THYM), and LIHC (Figure 1C). GEPIA was then employed to validate DTYMK mRNA expression. GEPIA combined the data from TCGA and GTEx databases, in which more tumor and normal samples were enrolled. DTYMK expression was increased in 19 cancer types including BLCA, CESC and colon adenocarcinoma (COAD) (Figure 1D). Using the CPTAC dataset from the UALCAN platform, DTYMK protein expression in several cancers was further validated. DTYMK protein expression was reduced in breast cancer and clear cell renal cell carcinoma when compared with normal tissues (Figure 2). In contrast, significantly enhanced DTYMK expression was found in COAD, LUAD, OV, and UCEC tissues (Figure 2). These results demonstrated that DTYMK expression was upregulated in most cancer types and might play an essential role in human cancer progression.

### DTYMK Expression Differentially Correlates With Cancer Stages, Immune Subtypes and Molecular Subtypes

**FIGURE 3:**
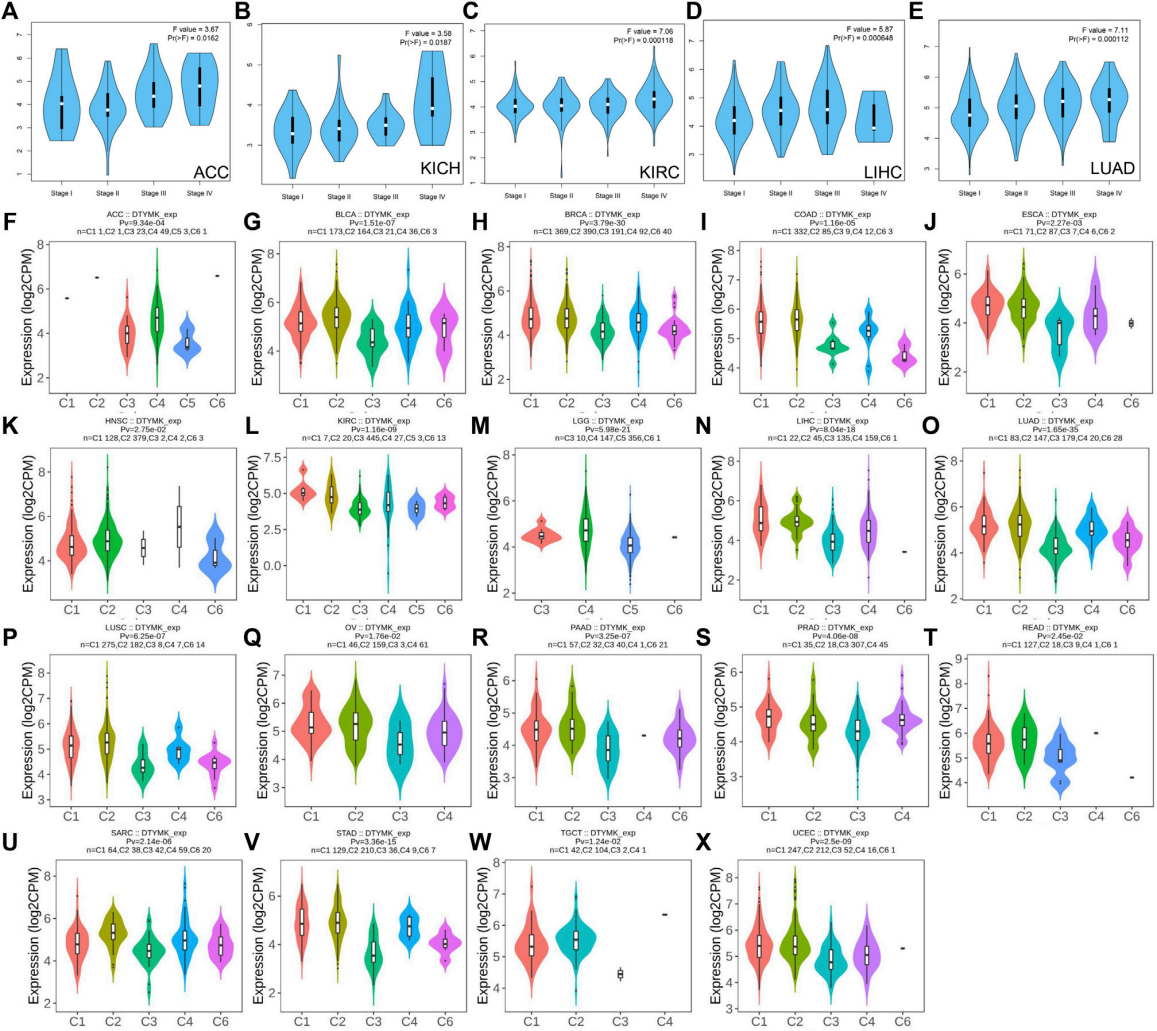
DTYMK expression is discriminately distributed in different stages and immune subtypes of cancers. Variation of DTYMK expression was observed in stage I, II, III and IV of ACC **(A)**, KICH **(B)**, KIRC **(C)**, LIHC **(D)** and LUAD **(E)** using the GEPIA database. DTYMK expression in different immune subtypes of ACC **(F)**, BLCA **(G)**, BRCA **(H)**, COAD **(I)**, ESCA **(J)**, HNSC **(K)**, KIRC **(L)**, LGG **(M)**, LIHC **(N)**, LUAD **(O)**, LUSC **(P)**, OV **(Q)**, PAAD **(R)**, PRAD **(S)**, READ **(T)**, SARC **(U)**, STAD **(V)**, TGCT **(W)** and UCEC **(X)**. C1, wound healing; C2, IFN-gamma dominant; C3, inflammatory; C4, lymphocyte depleted; C5, immunologically quiet; C6, TGF-*β* dominant. ACC, Adrenocortical carcinoma; BLCA, Bladder urothelial carcinoma; BRCA, Breast invasive carcinoma; COAD, Colon adenocarcinoma; ESCA, Esophageal carcinoma; HNSC, Head and neck squamous cell carcinoma; KIRC, Kidney renal clear cell carcinoma; LGG, Brain lower grade glioma; LIHC, Liver hepatocellular carcinoma; LUAD, Lung adenocarcinoma; LUSC, Lung squamous cell carcinoma; OV, Ovarian serous cystadenocarcinoma; PAAD, Pancreatic adenocarcinoma; PRAD, Prostate adenocarcinoma; READ, Rectum adenocarcinoma; SARC, Sarcoma; STAD, Stomach adenocarcinoma; TGCT, Testicular germ cell tumors; UCEC, Uterine corpus endometrial carcinoma.

To further explore DTYMK expression in human cancers obtained from different stages, we analyzed the relationship between DTYMK and cancer stages using GEPIA. In adrenocortical carcinoma (ACC), kidney chromophobe (KICH), kidney renal clear cell carcinoma (KIRC) and LUAD, stage IV tumor tissue was associated with higher expression of DTYMK compared to other stages (Figures 3A–C,E). However, DTYMK expression was decreased in stage IV LIHC tissues (Figure 3D).

**FIGURE 4:**
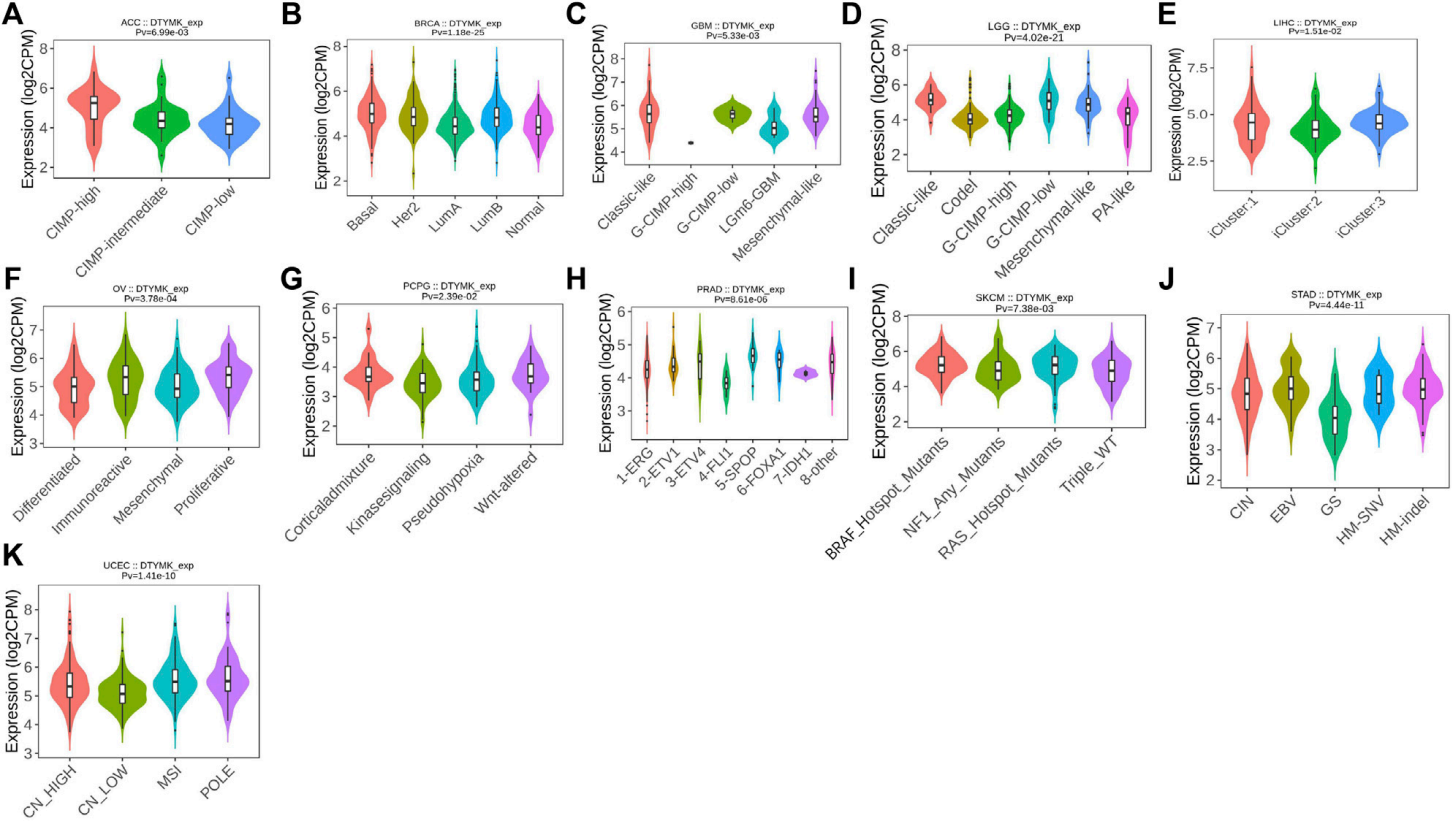
DTYMK expression is discriminately distributed in different molecular subtypes of cancers. Variation of DTYMK expression was shown in ACC **(A)**, BRCA **(B)**, GBM **(C)**, LGG **(D)**, LIHC **(E)**, OV **(F)**, PCPG **(G)**, PRAD **(H)**, SKCM **(I)**, STAD **(J)** and UCEC **(K)**. ACC, Adrenocortical carcinoma; BRCA, Breast invasive carcinoma; GBM, Glioblastoma multiforme; LIHC, Liver hepatocellular carcinoma; OV, Ovarian serous cystadenocarcinoma; PCPG, pheochromocytoma and paraganglioma; PRAD, Prostate adenocarcinoma; SKCM, Skin cutaneous melanoma; STAD, Stomach adenocarcinoma; UCEC, Uterine corpus endometrial carcinoma.

Immune subtypes of solid tumors have been divided into six categories according to the transcriptomic profiles of over 10,000 patients from The Cancer Genome Atlas (TCGA) cancer types (Thorsson et al., 2018). The six categories include C1 (wound healing), C2 (IFN-gamma dominant), C3 (inflammatory), C4 (lymphocyte depleted), C5 (immunologically quiet), C6 (TGF-*β* dominant) (Thorsson et al., 2018). C1 tumors are characterized by high induction of angiogenesis and proliferation; C2 are characterized by the highest M1/M2 macrophage polarization (towards M1); C3 are characterized by elevated Th17 and Th1 genes; C4 are characterized by macrophage infiltration and suppressed Th1 lymphocytes; C5 are characterized by the lowest lymphocyte and highest macrophage response and C6 are characterized by highest TGF-*β* signature and high lymphocyte infiltration (Thorsson et al., 2018). The variation of DTYMK expression in different subtypes was found in 19 cancers (Figures 3F–X). The top five (ranked by *p* value) cancers with high DTYMK expression included LUAD (*p* = 1.65e-35), BRCA (*p* = 3.79e-30), lower grade glioma (LGG) (*p* = 5.98e-21), LIHC (*p* = 8.04e-18), and STAD (*p* = 3.36e-15). We noticed that DTYMK expression was the lowest in C3 subtypes (inflammatory, high Th1 and Th17 infiltration) of human cancers, especially in BLCA, BRCA, ESCA, LUAD, LUSC, OV, PAAD, prostate adenocarcinoma (PRAD), READ, SARC, and STAD. DTYMK expression also differed in different molecular subtypes of cancers. Significant alteration of DTYMK expression was shown in 11 cancer types (Figures 4A–K). The top five cancers (ranked by *p* value) included BRCA (*p* = 1.18e-25), LGG (*p* = 4.02e-21), UCEC (*p* = 1.41e-10), PRAD (*p* = 8.61e-06), and OV (*p* = 3.78e-04). In BRCA, DTYMK expression was higher in basal-like, human epidermal growth factor receptor 2 (Her2)-enriched and luminal B subtypes compared with luminal A and normal-like subtypes. In LGG, DTYMK expression was the highest in glioma cytosine-phosphate-guanine island methylator phenotype (G-CIMP)-low subtype and the lowest in chromosome 1p/19q codeletion (Codel) subtype. In UCEC, DTYMK expression was downregulated in copy number low (CN_low) subtype. In PRAD, DTYMK expression was increased in speckle type BTB/POZ protein (SPOP) subtype and decreased in friend leukemia integration 1 (FLI1) subtype. In OV, DTYMK expression was downregulated in differentiated and mesenchymal subtype but upregulated in immunoreactive and proliferative subtype. Our results suggested that DTYMK expression varied among different immune and molecular subtypes.

### DTYMK Expression Positively or Negatively Correlates With Immune Cell Infiltration in Human Cancers

**FIGURE 5:**
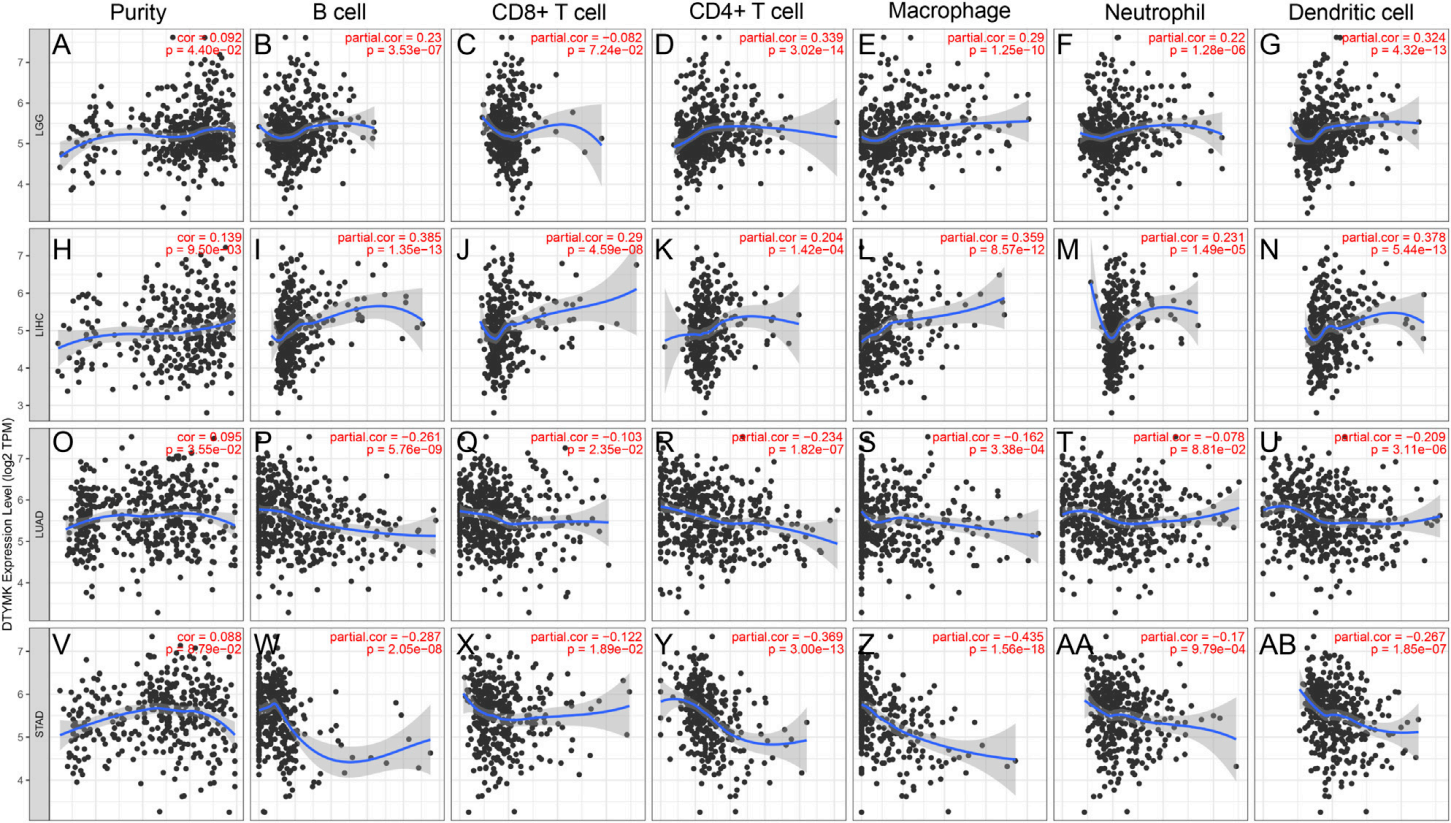
DTYMK expression is correlated with immune infiltration in cancers as analyzed using the TIMER database. DTYMK expression positively correlated with tumor purity in LGG **(A)**, LIHC **(H)**, LUAD (O) and STAD (V). In LGG, DTYMK expression positively correlated with infiltration of B cell **(B)**, CD4^+^ T cell **(D)**, macrophage **(E)**, neutrophil **(F)**, and dendritic cell **(G)**; In LIHC, DTYMK expression positively correlated with infiltration of B cell **(I)**, CD8^+^ T cell **(J)**, CD4^+^ T cell **(K)**, macrophage **(L)**, neutrophil **(M)**, and dendritic cell **(N)**; In LUAD, DTYMK expression negatively correlated with infiltration of B cell **(P)**, CD8^+^ T cell **(Q)**, CD4^+^ T cell **(R)**, macrophage **(S)**, neutrophil **(T)**, and dendritic cell **(U)**; In STAD, DTYMK expression negatively correlated with infiltration of B cell **(W)**, CD8^+^ T cell **(X)**, CD4^+^ T cell **(Y)**, macrophage **(Z)**, neutrophil **(AA)**, and dendritic cell **(AB)**. LGG, Brain lower grade glioma; LIHC, Liver hepatocellular carcinoma; LUAD, Lung adenocarcinoma; STAD, Stomach adenocarcinoma.

The TIMER database was employed to elucidate whether DTYMK impacted on immune infiltration in human cancers. The results showed that DTYMK expression was significantly correlated with the infiltration of six types of immune cells, which included B cell, CD8^+^ T cell, CD4^+^ T cell, macrophage, neutrophil and dendritic cell (Supplementary Figures S2A,B). Among all the tumors, LGG, LIHC, LUAD, and STAD were the most closely correlated cancer types with DTYMK expression (Figures 5A–AB). Notably, DTYMK expression was positively correlated with the infiltration of B cell (LGG: *p* = 3.53e-07; LIHC: *p* = 1.35e-13), CD4^+^ T cell (LGG: *p* = 3.02e-14; LIHC: *p* = 1.42e-04), macrophage (LGG: *p* = 1.25e-10; LIHC: *p* = 8.57e-12), neutrophil (LGG: *p* = 1.28e-06; LIHC: *p* = 1.49e-05) and dendritic cell (LGG: *p* = 4.32e-13; LIHC: *p* = 5.44e-13) in LGG and LIHC (Figures 5A–N). In contrast, in LUAD and STAD, DTYMK expression was negatively correlated with the infiltration of B cell (LUAD: *p* = 5.76e-09; STAD: *p* = 2.05e-08), CD8^+^ T cell (LUAD: *p* = 2.35e-02; STAD: *p* = 1.89e-02), CD4^+^ T cell (LUAD: *p* = 1.82e-07; STAD: *p* = 3.00e-13), macrophage (LUAD: *p* = 3.38e-04; STAD: *p* = 1.56e-18), and dendritic cell (LUAD: *p* = 3.11e-06; STAD: *p* = 1.85e-07) (Figures 5O–AB). These results suggested DTYMK might play distinct roles in immune infiltration in different cancer types.

**FIGURE 6:**
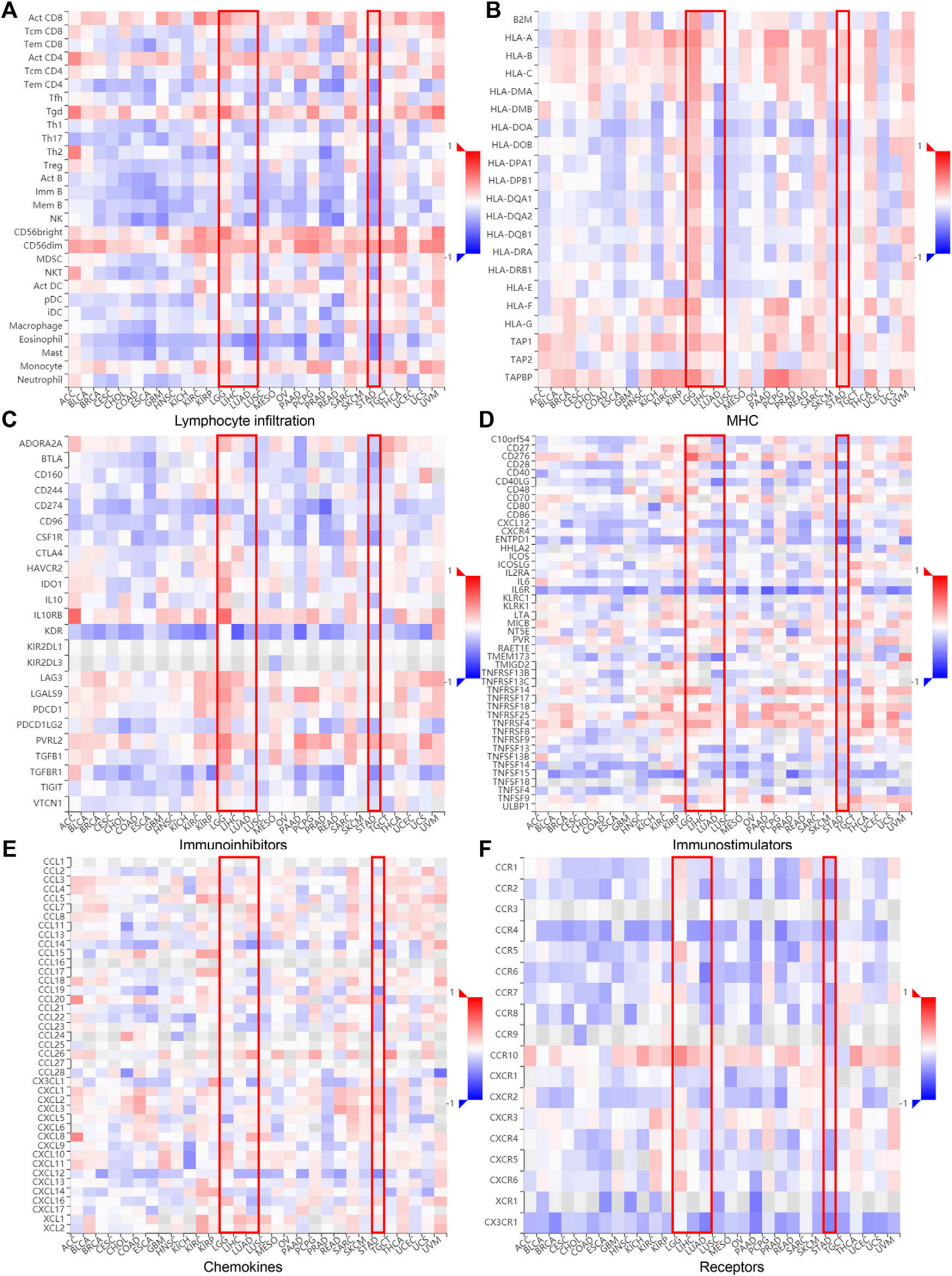
DTYMK expression is linked to immune infiltration as analyzed using the TISIDB database. DTYMK expression affected lymphocyte infiltration **(A)**, MHC expression **(B)**, immune inhibitors expression **(C)**, immunostimulator expression **(D)**, chemokine expression **(E)** and chemokine receptors expression **(F)** in human cancers.

The relationship between DTYMK and immune infiltration was further confirmed using the TISIDB database, which explored 28 types of infiltrated lymphocytes (Supplementary File S1). Although DTYMK was positively correlated with the infiltration of activated CD4^+^ T cells and CD8^+^ T cells in LGG, LIHC, LUAD, and STAD, effector memory CD4^+^ T and CD8^+^ T infiltration was inversely correlated with DTYMK expression (Figure 6A). The infiltration of CD4^+^ T cellsubsets, Th1, Th2 and Th17, was also negatively correlated with DTYMK expression in LGG, LIHC, LUAD, and STAD (Figure 6A). Moreover, follicular helper T cell infiltration was positively correlated with DTYMK in LGG, but was negatively correlated in LUAD and STAD (Figure 6A). Lastly, the infiltration of innate immune cells including eosinophils and natural killer (NK) cells was also negatively correlated with DTYMK in LIHC, LUAD and STAD (Figure 6A). These results revealed the contrasting effects of DTYMK on adaptive and innate immune cells infiltration in different cancers.

The correlation between DTYMK and the major histocompatibility complex (MHC), immune inhibitors, immune stimulators, chemokines, and receptors expressed in the tumor microenvironment (TME) were also inspected across 30 cancer types (Figures 6B–F; Supplementary File S1). Interestingly, DTYMK positively impacted MHC class II molecules (HLA-DP, HLA-DM, HLA-DO, HLA-DQ, HLA-DR) expression in LGG but negatively impacted MHCII expression in LUAD and STAD (Figure 6B). Poliovirus receptor-related 2 (PVRL2), an immunoinhibitor, binds with the coinhibitory receptor T cell immunoreceptor with Ig and ITIM domains (TIGIT) and negatively regulates lymphocyte activity (Stamm et al., 2018). It has been shown that interleukin-6 (IL-6)/IL-6R signaling is involved in the stimulation of Th17 cell differentiation from naive T cells (Yamashita et al., 2011). Our further analysis uncovered that high expression of DTYMK positively influenced PVRL2 expression but negatively influenced IL-6R expression in LGG, LIHC, LUAD and STAD (Figures 6C,D; Supplementary File S1). These results suggested that DTYMK expression not only affected immune cell infiltration, but also influenced major histocompatibility complex (MHC) expression, immune cell activities through regulating immune inhibitors, chemokines/cytokines and their receptor expression.

### GSEA Analysis Reveals a Role for DTYMK in Pyrimidine Metabolism and Th Cell Differentiation

**FIGURE 7:**
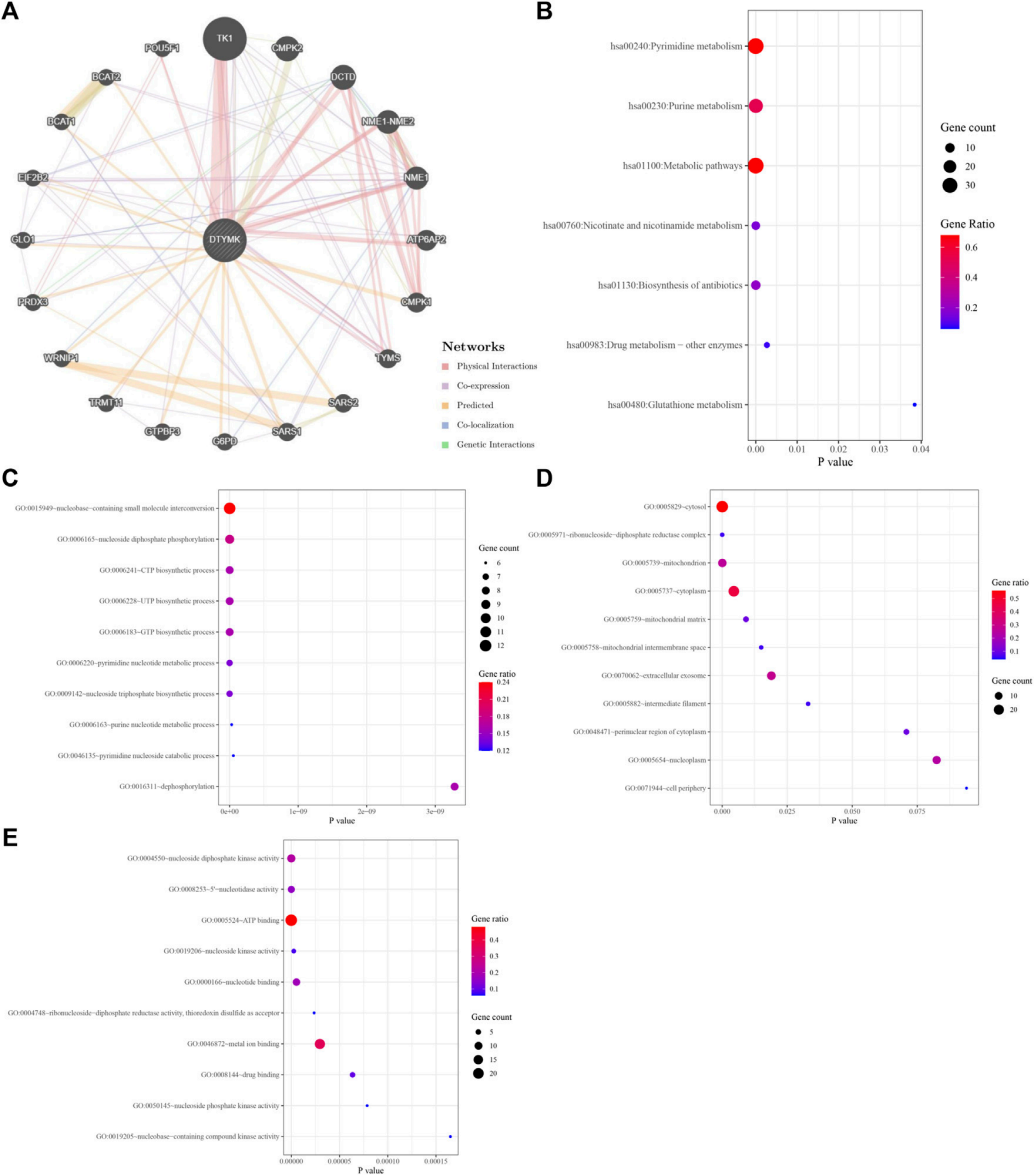
Interactive genes of DTYMK and gene ontology (GO) analysis. **(A)** DTYMK interacted with TK1 and other genes in GeneMANIA database. Top 50 interactive genes of DTYMK were obtained from STRING database and processed for Kyoto Encyclopedia of Genes and Genomes (KEGG) analysis **(B)**. GO enrichment analyses include biological process **(C)**, cellular component **(D)**, and molecular function **(E)** by DAVID platform.

To study the molecular role of DTYMK in tumorigenesis we used the GeneMANIA platform to plot a protein-protein interaction (PPI) network for DTYMK. DTYMK strongly interacted with thymidine kinase 1 (TK1) (Figure 7A). To clarify DTYMK functions in tumor cells, the top 50 proteins interacting with DTYMK were obtained from the STRING database (Supplementary File S2) and processed for KEGG and GO enrichment analyses using the DAVID platform (Figures 7B–E; Supplementary File S3–S6). As shown in the figure, “pyrimidine metabolism”, “purine metabolism” and “metabolic pathways” are critical pathways in which DTYMK functions in human cancers (Figure 7B).

**FIGURE 8:**
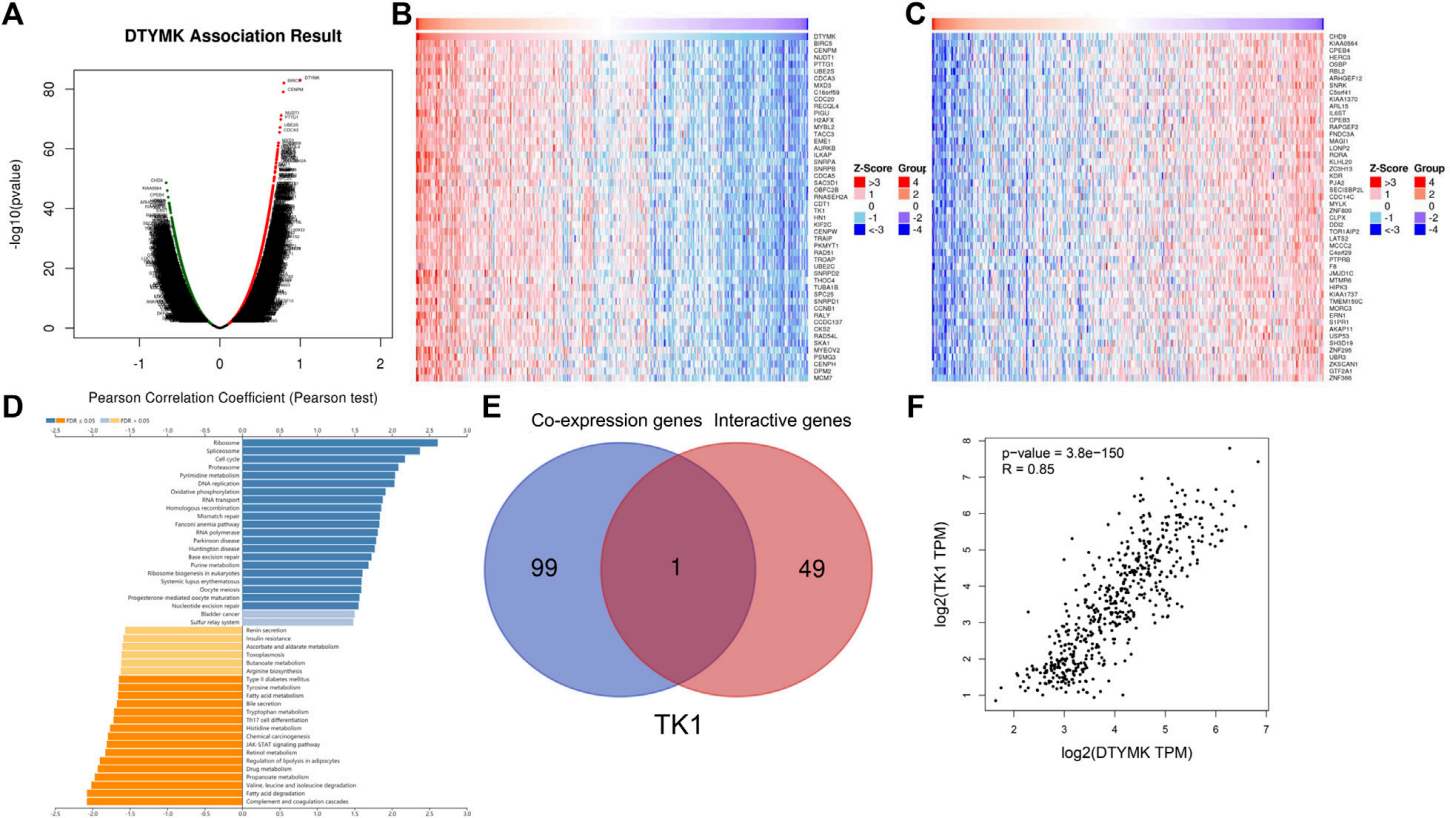
Analysis of DTYMK co-expressing genes in liver hepatocellular carcinoma. **(A)** DTYMK co-expressing genes were shown by Volcano plot. Top 50 genes that positively **(B)** and negatively **(C)** correlated with DTYMK are shown by heatmap plot. **(D)** Gene Set Enrichment Analysis (GSEA) of DTYMK co-expressing genes. **(E)** Common gene thymidine kinase 1 (TK1) was obtained by the intersection of DTYMK interactive genes and DTYMK co-expressing genes. **(F)** The relation between DTYMK expression and TK1 expression was validated by GEPIA database.

**FIGURE 9:**
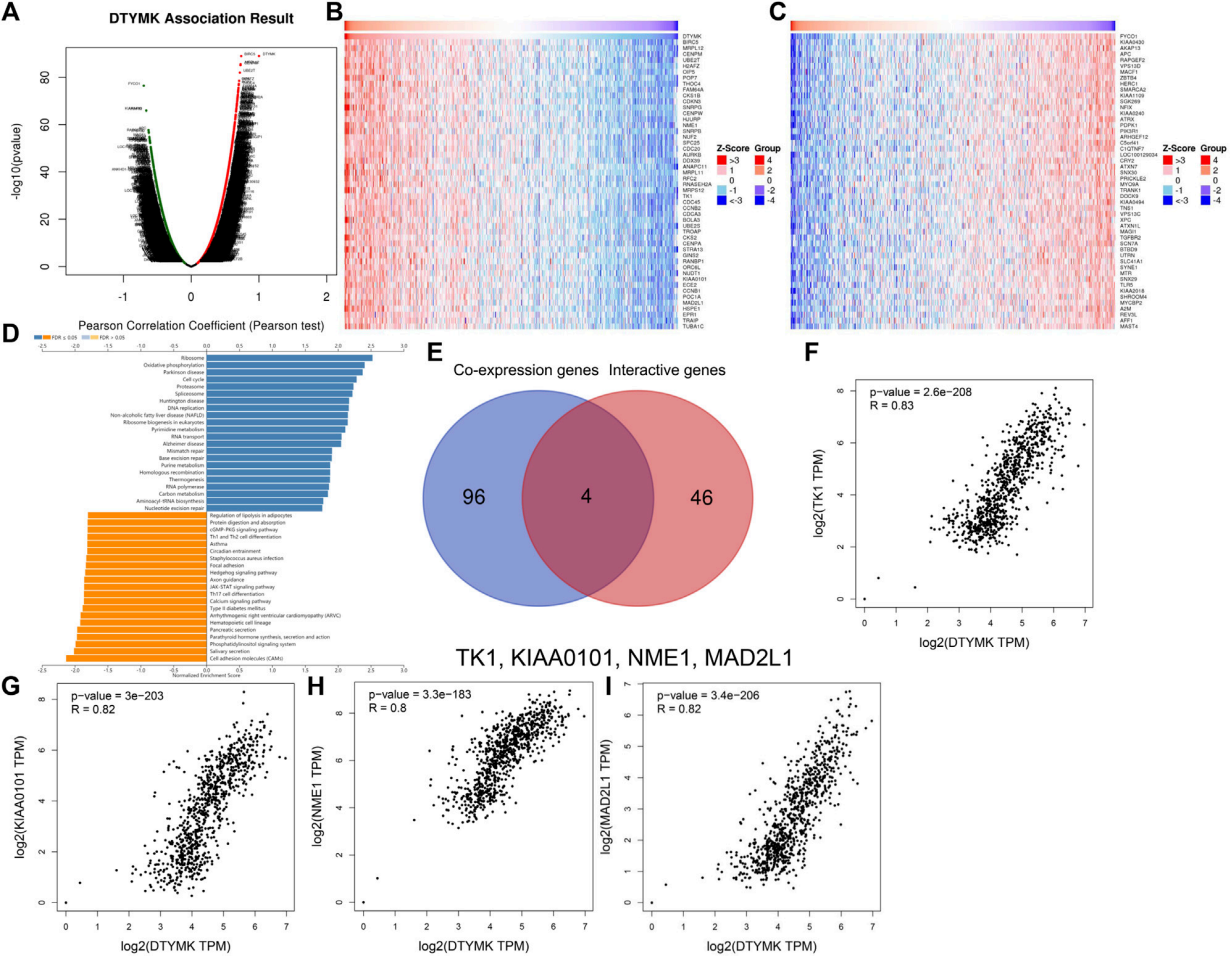
Analysis of DTYMK co-expressing genes in lung adenocarcinoma. **(A)** DTYMK co-expressing genes are shown by Volcano plot. Top 50 genes that positively **(B)** and negatively **(C)** correlated with DTYMK are shown by heatmap plot. **(D)** Gene Set Enrichment Analysis (GSEA) of DTYMK co-expressing genes. **(E)** Common genes including thymidine kinase 1 (TK1), proliferating cell nuclear antigen clamp associated factor (KIAA0101), NME/NM23 nucleoside diphosphate kinase 1 (NME1), mitotic arrest deficient 2 like 1 (MAD2L1) were obtained by the intersection of DTYMK interactive genes and DTYMK co-expressing genes. The relations between DTYMK expression and TK1 expression **(F)**, KIAA0101 expression **(G)**, NME1 expression **(H)**, MAD2L1 expression **(I)** were validated by GEPIA database.

Considering that DTYMK expression was upregulated and also correlated with poor prognosis and immune infiltration in LIHC and LUAD, we then focused on the functions of DTYMK in LIHC and LUAD. DTYMK co-expressing genes in LIHC and LUAD were analyzed using the Linkedomics database (Supplementary Files S7, S8). All correlated genes were shown by Volcano plots (Figures 8A, 9A). The top 50 genes that positively and negatively correlated with DTYMK were indicated in the heatmap plot (Figures 8B,C, Figures 9B,C). GSEA analysis of DTYMK-correlating genes was then carried out. Coinciding with the enrichment results we determined using DAVID, the results of KEGG enrichment underlined that positively correlated genes of DTYMK were involved in “cell cycle,” “pyrimidine metabolism,” and “purine metabolism” pathways in LIHC and LUAD (Figures 8D, 9D). What is noteworthy is that negatively correlated genes were enriched in “Th1 and Th2 cell differentiation” or/and “Th17 cell differentiation” in LIHC and LUAD (Figures 8D, 9D). The results emphasized that DTYMK might be implicated in regulating helper T cell differentiation. Additionally, the intersection between DTYMK interactive genes by STRING and DTYMK co-expressing genes showed that TK1 was the only common gene for LIHC (Figure 8E), while TK1, proliferating cell nuclear antigen clamp associated factor (KIAA0101), NME/NM23 nucleoside diphosphate kinase 1 (NME1), mitotic arrest deficient 2 like 1 (MAD2L1) were common genes for LUAD (Figure 9E). The positive correlations between DTYMK and TK1, KIAA0101, NME1, and MAD2L1 were further validated in GEPIA platform (Figures 8F, 9F–I). Using the drug bank database we found that the metabolism of four drugs led to the upregulation of DTYMK while nine drugs led to the downregulation (Table 1). Their effects on DTYMK expression should be noted considering its potential role in human cancers progression.

**TABLE 1:** Pharmaco-transcriptomics analysis of DTYMK using Drugbank database.

Drug	Change	Interaction	References (PMID)
Acetaminophen	upregulated	Acetaminophen results in increased expression of DTYMK mRNA	22230336
Estradiol	upregulated	Estradiol results in increased expression of DTYMK mRNA	16474171
Copper	downregulated	[NSC 689534 binds to Copper] which results in decreased expression of DTYMK mRNA	20971185
Cyclosporine	downregulated	Cyclosporine results in decreased expression of DTYMK mRNA	20106945 25562108
Calcitriol	downregulated	Calcitriol results in decreased expression of DTYMK mRNA	21592394
Epigallocatechin gallate	downregulated	epigallocatechin gallate results in decreased expression of DTYMK mRNA	18851785
Fluorouracil	upregulated	Fluorouracil results in increased expression of DTYMK mRNA	15109396
Genistein	upregulated	Genistein results in increased expression of DTYMK mRNA	16474171
Nicotine	downregulated	Nicotine results in decreased expression of DTYMK mRNA	16949557
Piroxicam	downregulated	Piroxicam results in decreased expression of DTYMK mRNA	21858171
Testosterone	downregulated	Testosterone results in decreased expression of DTYMK mRNA	21592394
Troglitazone	downregulated	troglitazone results in decreased expression of DTYMK mRNA	19140230
Valproic acid	downregulated	Valproic Acid results in decreased expression of DTYMK mRNA	26272509

### DTYMK Expression Indicates Poor Prognosis in Human Cancers

**FIGURE 10:**
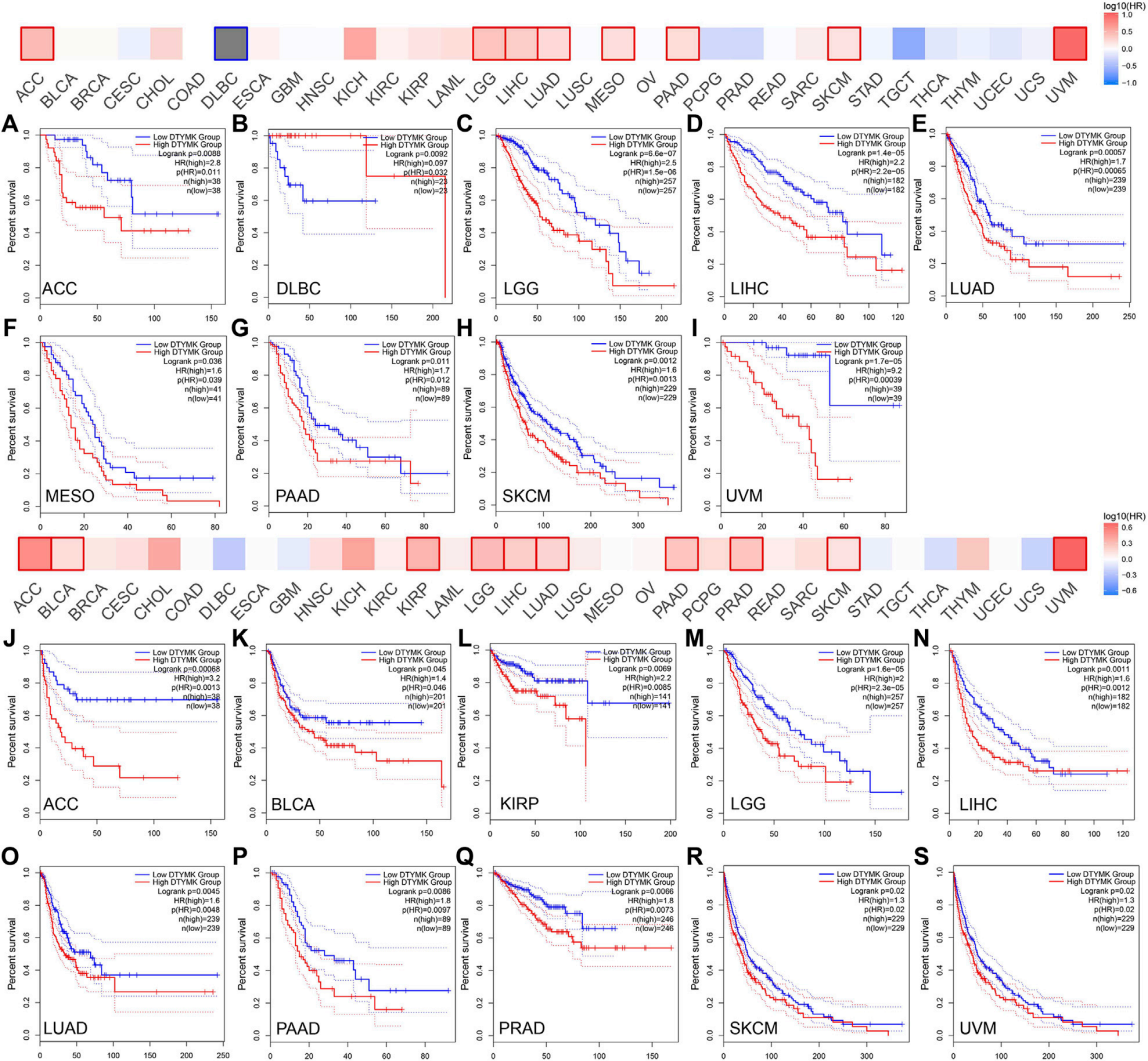
The GEPIA database suggests that higher DTYMK expression correlates with worse prognosis in most cancers. DTYMK expression was significantly correlated with overall survival of ACC **(A)**, DLBC **(B)**, LGG **(C)**, LIHC **(D)**, LUAD **(E)**, MESO **(F)**, PAAD **(G)**, SKCM **(H)**, and UVM **(I)**. DTYMK expression was significantly correlated with disease-free survival of ACC **(J)**, BLCA **(K)**, KIRP **(L)**, LGG **(M)**, LIHC **(N)**, LUAD **(O)**, PAAD **(P)**, PRAD **(Q)**, SKCM **(R)**, and UVM **(S)**. ACC, Adrenocortical carcinoma; DLBC, diffuse large B-cell lymphoma; BLCA, Bladder urothelial carcinoma; KIRP, Kidney renal papillary cell carcinoma; LGG, Brain lower grade glioma; LIHC, Liver hepatocellular carcinoma; LUAD, Lung adenocarcinoma; LUSC, Lung squamous cell carcinoma; MESO, Mesothelioma; PAAD, Pancreatic adenocarcinoma; PRAD, Prostate adenocarcinoma; SKCM, Skin cutaneous melanoma; UVM, Uveal melanoma.

Given that DTYMK expression varied across human cancers and correlated to cancer stages and immune subtypes as well as immune cell infiltrations, it is conceivable that DTYMK might be a potential prognostic marker. This was first evaluated in human cancers using GEPIA. Higher DTYMK expression represented worse OS in cancers including ACC (*p* = 0.0088), LGG (*p* = 6.6e-07), LIHC (*p* = 1.4e-05), LUAD (*p* = 0.00057), mesothelioma (MESO) (*p* = 0.036), PAAD (*p* = 0.011), skin cutaneous melanoma (SKCM) (*p* = 0.0012) and uveal melanoma (UVM) (*p* = 1.7e-05) (Figures 10A,C–I). Intriguingly, DTYMK acted as a protective factor for OS of diffuse large B-cell lymphoma (DLBC) patients (*p* = 0.0092) (Figure 10B). Furthermore, the relationship between DTYMK expression and patients’ relapse-free survival (RFS) was also explored. Higher expression of DTYMK was correlated with shorter RFS in cancers including ACC (*p* = 0.00068), BLCA (*p* = 0.045), KIRP (*p* = 0.0069), LGG (*p* = 1.6e-05), LIHC (*p* = 0.011), LUAD (*p* = 0.0045), PAAD (*p* = 0.0086), PRAD (*p* = 0.0066), SKCM (*p* = 0.02), and UVM (*p* = 0.02) (Figures 10J–S).

**FIGURE 11:**
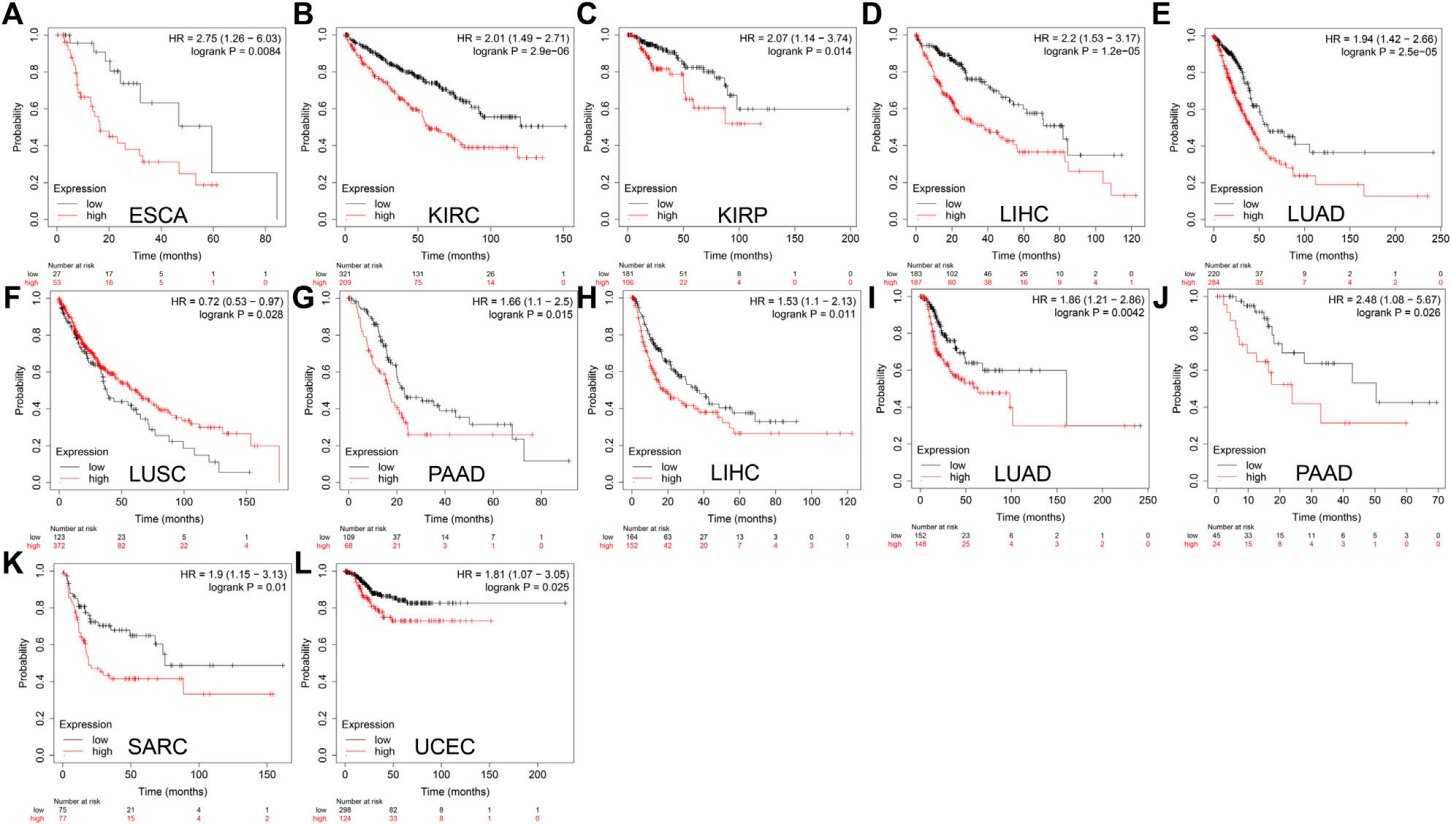
Kaplan-Meier analysis suggests that enhanced DTYMK expression correlated with a poor outcome in most cancers. DTYMK expression was significantly correlated with overall survival of ESCA **(A)**, KIRC **(B)**, KIRP **(C)**, LIHC **(D)**, LUAD **(E)**, LUSC **(F)**, and PAAD **(G)**. DTYMK expression was significantly correlated with disease-free survival of LIHC **(H)**, LUAD **(I)**, PAAD **(J)**, SARC **(K)** and UCEC **(L)**. ACC, Adrenocortical carcinoma; BLCA, Bladder urothelial carcinoma; BRCA, Breast invasive carcinoma; COAD, Colon adenocarcinoma; ESCA, Esophageal carcinoma; HNSC, Head and neck squamous cell carcinoma; KIRC, Kidney renal clear cell carcinoma; LGG, Brain lower grade glioma; LIHC, Liver hepatocellular carcinoma; LUAD, Lung adenocarcinoma; LUSC, Lung squamous cell carcinoma; OV, Ovarian serous cystadenocarcinoma; PAAD, Pancreatic adenocarcinoma; PRAD, Prostate adenocarcinoma; READ, Rectum adenocarcinoma; SARC, Sarcoma; STAD, Stomach adenocarcinoma; TGCT, Testicular germ cell tumors; UCEC, Uterine corpus endometrial carcinoma.

The relationship between DTYMK expression and patient prognosis was then determined using Kaplan-Meier analysis. Data used in the Kaplan-Meier plotter, including gene expression, RFS and OS information were sourced from GEO, EGA and TCGA. It was found that higher DTYMK expression was correlated with shorter OS in cancers like ESCA (*p* = 0.0084), KIRC (*p* = 2.9e-06), KIRP (*p* = 0.014), LIHC (*p* = 1.2e-05), LUAD (*p* = 2.5e-05), and PAAD (*p* = 0.015) (Figures 11A–E,G). However, increased expression of DTYMK in LUSC indicated a prolonged OS (*p* = 0.028) (Figure 11F). Upregulated DTYMK expression also reflected worse RFS in LIHC (*p* = 0.011), LUAD (*p* = 0.0042), PAAD (*p* = 0.026), SARC (*p* = 0.01), and UCEC (*p* = 0.025) (Figures 11H–L).

**FIGURE 12:**
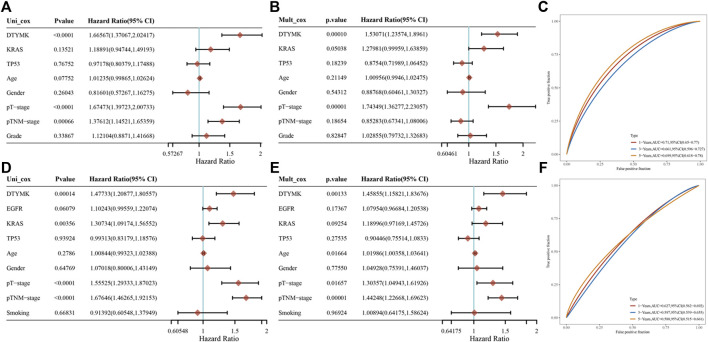
Prognostic function of DTYMK in LIHC and LUAD. Univariate Cox analysis of DTYMK expression and clinical parameters of LIHC **(A)** and LUAD **(D)**. Multivariate Cox analysis of DTYMK expression and clinical parameters of LIHC **(B)** and LUAD **(E)**. The time-dependent receiver operating characteristic (ROC) and area under the curve (AUC) of DTYMK for LIHC **(C)** and LUAD **(F)**.

The results from the GEPIA and Kaplan-Meier analysis showed that DTYMK expression significantly correlated with survival (OS and RFS) of patients with LIHC and LUAD. To further clarify the prognostic role of DTYMK in LIHC and LUAD, the Cox proportional hazard regression model was established to evaluate prognostic factors. LIHC and LUAD patients were divided into groups with high or low DTYMK expression based on median DTYMK expression. Both the univariate Cox analysis and multivariate Cox analysis results demonstrated that higher DTYMK expression correlated with worse prognosis in LIHC and LUAD (Figures 12A,B,D,E). We then employed receiver operating characteristic (ROC) analysis to demonstrate the diagnostic efficacy of DTYMK in LIHC and LUAD (Figures 12C,F). The area under curve (AUC) value was 0.71, 0.661 and 0.699 for 1, 3, and 5 years OS of LIHC, while the AUC value was 0.627, 0.597, and 0.588 for 1, 3, and 5 years OS of LUAD. The results indicated that DTYMK expression might powerfully predict prognosis in LIHC and LUAD.

**FIGURE 13:**
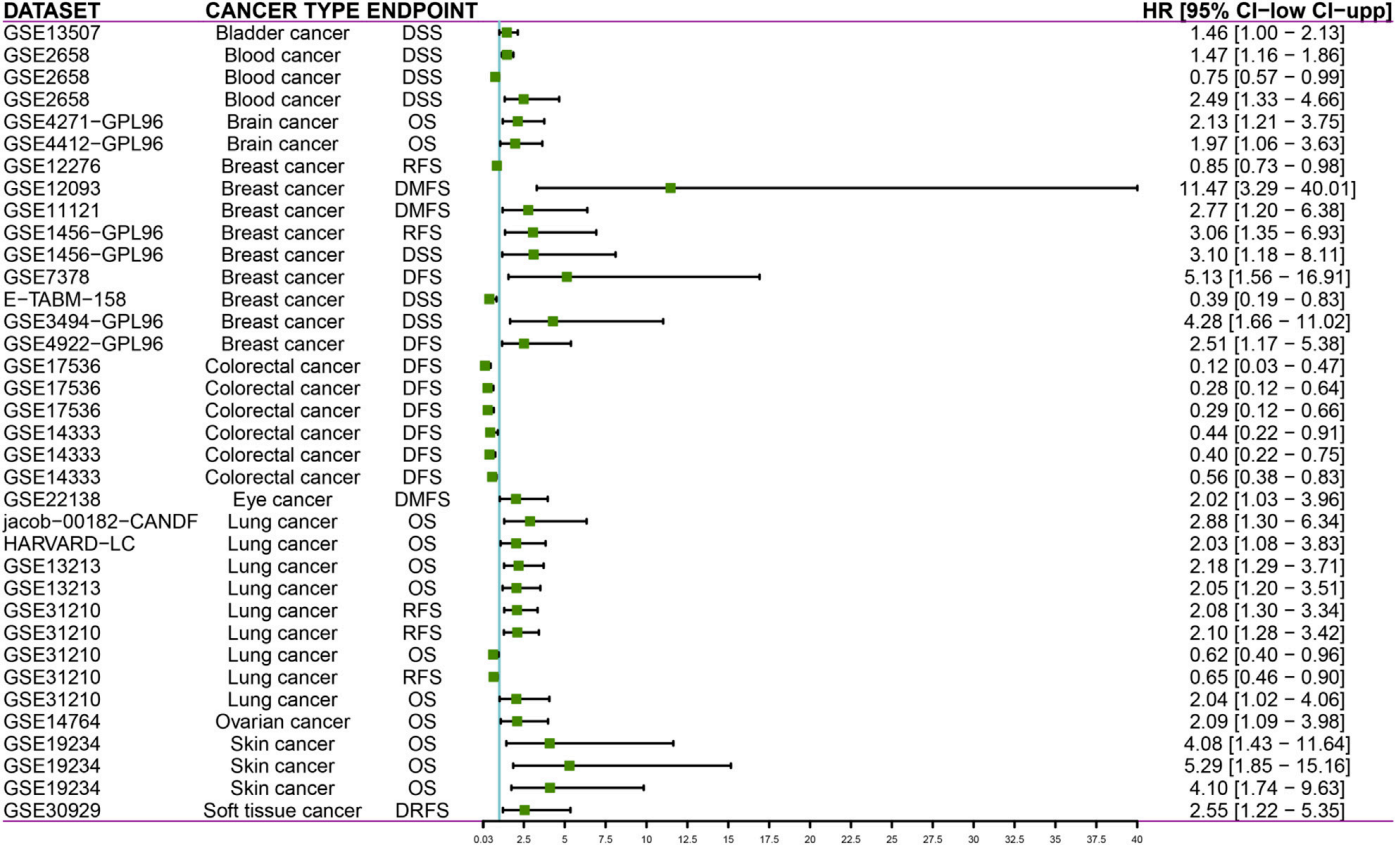
PrognoScan databases suggest that increased DTYMK expression correlates with different outcomes in cancers. DSS, disease specific survival; OS, overall survival; RFS, relapse free survival; DFS, disease free survival; DMFS, distant metastasis free survival; DRFS, distant recurrence free survival.

PrognoScan database facilitates the investigation of gene association with clinical outcome by encompassing a collection of publicly available cancer microarray datasets. Our results showed that DTYMK expression significantly influenced the prognosis of some cancers (Figure 13; Supplementary File S9). In some cancers like bladder, blood, brain, breast, eye, lung, ovarian, skin and soft tissue cancers, elevated expression of DTYMK resulted in poorer prognosis (Figure 13; Supplementary File S9). On the contrary, DTYMK played a protective role in colorectal cancer (Figure 13; Supplementary File S9).

### DTYMK Regulates Tumor Cell Proliferation and Migration *in vitro*

**FIGURE 14:**
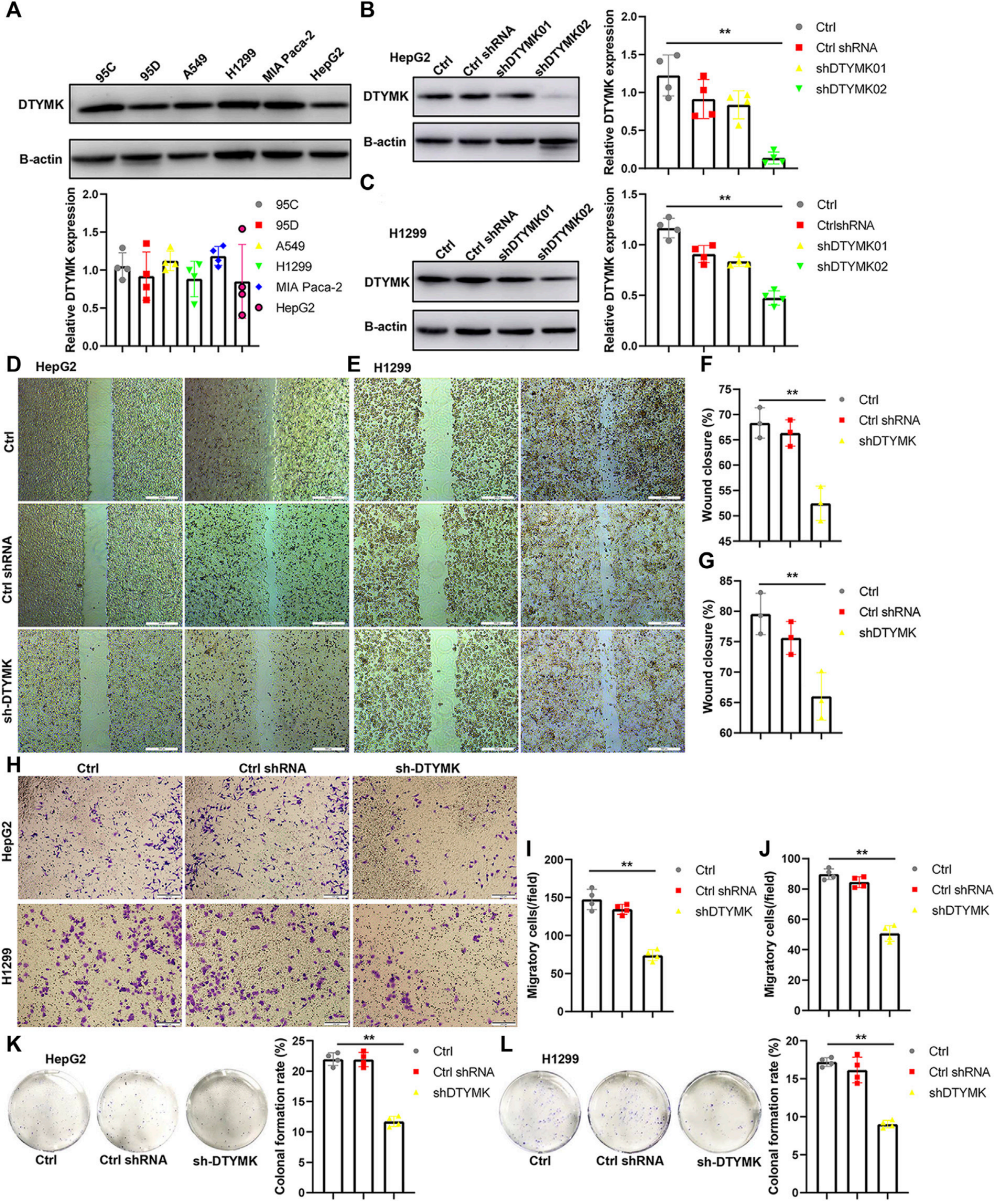
Knockdown of DTYMK expression suppressed cell migration and colony formation. **(A)** DTYMK protein expression in multiple tumor cell lines, including 95C, 95D, A549, H1299, MIA Paca2, and HepG2. **(B) (C)** shDTYMK02 rather than shDTYMK01 knocked down DTYMK expression in HepG2 and H1299 cells. **(D–G)** Knockdown of DTYMK expression dampened cell migration of HepG2 and H1299 cells detected by wound healing assay. **(H–J)** Knockdown of DTYMK expression dampened cell migration of HepG2 and H1299 cells detected by Transwell assay. **(K–L)** Knockdown of DTYMK expression inhibited plate colony formation of HepG2 and H1299 cells. **p < 0.01.

To validate DTYMK function in tumor cells, we examined DTYMK expression in tumor cell lines of various origins (lung cancer including 95C, 95D, A549, and H1299; pancreatic cancer MIA PaCa-2; liver cancer HepG2) (Figure 14A). Two short hairpin RNAs were designed to downregulate DTYMK expression in HepG2 cells and H1299 cells. Compared with ctrl shRNA, shDTYMK02 but not shDTYMK01 markedly reduced DTYMK expression in both tumor cell lines (Figures 14B,C). shDTYMK02 was used to stably disrupt DTYMK expression in cancer cell lines. Subsequently, knockdown of DTYMK impeded cell migration and clonal formation in HepG2 and H1299 cells (Figures 14D–L). Our results confirmed the role of DTYMK in regulating tumor cell migration and clonal formation.

## Discussion

Besides the function of DTYMK in thymidylate phosphorylation, its role in tumorigenesis has been investigated by some researchers. The kinase property of DTYMK has been harnessed for phosphorylating 3′-azido-3′-deoxythymidine (AZT) into AZT-triphosphate (AZT-TP) and disrupting the tumor cell cycle (Sato et al., 2007; Sato et al., 2013). Moreover, DTYMK is required for DNA double-strand break repair in tumor cells *via* interactions with ribonucleotide reductase and restriction of dUTP incorporation (Hu et al., 2012). In hepatocellular carcinoma (HCC), DTYMK has been noted to serve as prognostic and chemotherapeutic response biomarker and positively correlated with tumor immune infiltration (Guo et al., 2021). Our study further demonstrate that DTYMK is a prognostic marker in several cancer types, especially in LGG, LIHC, LUAD, and STAD. This study, together with others, supports and strengthens the significance of DTYMK as a target for cancer therapy.

In the present study, examination of DTYMK expression was performed using four platforms: Oncomine, TIMER, GEPIA and UALCAN. The Oncomine database integrates public cancer microarray data with gene ontology annotations and therapeutic target databases (Rhodes et al., 2004). Increased DTYMK expression could be visualized across various cancer types. The Oncomine platform has been exploited for data-mining in many studies (Ning et al., 2018; Kong et al., 2020). Both TIMER and GEPIA databases contain high-throughput RNA sequencing data of 33 cancer types from TCGA (Li et al., 2017; Tang et al., 2017). GEPIA also includes RNA sequencing data from Genotype-Tissue Expression (GTEx). The results from these two databases commonly revealed that DTYMK expression was elevated in BLCA, CESC, COAD, glioblastoma multiforme (GBM), head and neck squamous cell carcinoma (HNSC), LIHC, LUAD, LUSC, rectum adenocarcinoma (READ), STAD and UCEC. Furthermore, mass-spectrometry-based proteomic-profiling data from UALCAN allows analysis of protein expression in six cancer types: breast, colon, ovarian, renal, and uterine cancer (Chen et al., 2019). DTYMK protein expression was decreased in breast and renal cancer, but it was increased in colon, lung, ovarian and uterine cancer. The discrepancy between DTYMK mRNA and protein expression might be attributed to a smaller sample volume in the CPTAC dataset (TCGA vs. CPTAC, breast cancer: 1084 vs. 125, renal cancer: 512 vs. 110).

Through analysis, we found that augmentation of DTYMK expression was linked to poor prognosis in several types of cancer. Notably, the prognostic potential (OS and RFS) of DTYMK in LIHC and LUAD was confirmed by both GEPIA and Kaplan-Meier plotter databases. These results were consistent with those reported by Wang et al. (Wang et al., 2020), supporting the involvement of DTYMK in affecting disease outcome in patients. Further clarification regarding the mechanism by which DTYMK influences patient survival is urgently required.

Thorsson et al. (Thorsson et al., 2018) integrated immunogenomics methods and characterized the immune TME across 33 cancer types compiled in the TCGA database. Six immune subtypes were established to fractionate human cancers and these were applied to predict disease outcome spanning multiple cancers (Thorsson et al., 2018). We found that DTYMK expression varied among cancer immune subtypes. Interestingly, DTYMK expression was the lowest in C3 subtypes (inflammatory, high Th1 and Th17 infiltration) of human cancers, especially in BLCA, BRCA, ESCA, LUAD, LUSC, OV, PAAD, PRAD, READ, SARC, and STAD. We speculated that DTYMK expression might inversely regulate Th1 and Th17 infiltration. Indeed, the infiltration of Th1, Th2 and Th17 was inhibited by upregulated DTYMK in LGG, LIHC, LUAD, and STAD according to the TISIDB database. DTYMK-correlated genes determined using GSEA further demonstrated that a cluster of negative co-expressing genes participated in Th1, Th2 and Th17 cell differentiation. Th1/Th2 ratio and Th17/Treg ratio have been demonstrated as critical factors that impact on tumor progression (Knochelmann et al., 2018; Anichini et al., 2020). Therefore, the effect of DTYMK on Th1, Th2 and Th17 cell differentiation may contribute to poor prognosis in patients. Our results predicted that genes correlated with DTYMK were involved in the “cell cycle”. As such, DTYMK might affect cancer cell proliferation and this requires further *in vitro* characterization.

In a previous study, DTYMK expression positively correlated with the infiltration of Tfhs, Tregs and M0 macrophages in liver cancer (Guo et al., 2021). Notably, our results indicated a distinct correlation between DTYMK expression and immune infiltration in cancers like LGG, LIHC, LUAD and STAD, suggesting a complex interplay between DTYMK and tumor immune response. Although DTYMK positively affected infiltration of activated CD4^+^ T cell and CD8^+^ T cell in LGG, LIHC, LUAD, and STAD, augmented DTYMK suppressed infiltration of effector memory CD4^+^ T and CD8^+^ T cells. Moreover, high expression of DTYMK suppressed the infiltration of innate immune cells like eosinophils and NK cells in LIHC, LUAD and STAD. Eosinophils derive from CD34^+^ progenitor cells located in the bone marrow. Eosinophils that infiltrate into tumor environment play both antitumorigenic roles and pro-tumorigenic roles, as they can release interleukins (like IL-10, IL-12 and IFN-*γ*) or chemokines and growth factors (like EGF, FGF and TGF-*β*) (Rothenberg and Hogan, 2006; Spencer et al., 2009). NK cells are innate lymphoid cells and derive from CD34^+^ haematopoietic progenitors in the bone marrow (Yu et al., 2013). The prognostic value of NK cell infiltration has been demonstrated in some cancers (Nersesian et al., 2021). Notably, DTYMK expression was positively correlated with the infiltration of macrophages, neutrophil and DCs in Figure 5A. In contrast, DTYMK expression was negatively correlated with the infiltration of macrophages, neutrophils and DCs in Figure 6A. Figure 5 results are derived from the TIMER database (https://cistrome.shinyapps.io/timer/), while Figure 6A results are derived from the TISIDB database (http://cis.hku.hk/TISIDB/index.php). The discrepancy between them could be attributed to the data collection and calculation algorithm. The TIMER database encompasses gene expression data, which includes 10,897 samples across 32 cancer types from The Cancer Genome Atlas (TCGA) and was then calculated using the TIMER algorithm. The TISIDB database encompasses literature mining results from PubMed database, high throughput screening data, exome and RNA sequencing data, data from The Cancer Genome Atlas (TCGA) and other public databases. TISIDB calculates the relationship between gene expression and tumor-infiltrating lymphocytes by Spearman correlation.

We acknowledge that there are several limitations in our study. Firstly, augmented DTYMK expression in multiple cancers needs to be confirmed in tumor tissues using different methods such as qPCR and immunohistochemistry. Secondly, although DTYMK expression was closely linked with Th1, Th2 and/or Th17 cell differentiation, the role and mechanism of DTYMK involvement in Th cell differentiation needs to be validated. Lastly, it remains unknown whether DTYMK expression is correlated with mutations of “driver genes” such as TP53, KRAS or PTEN. Their correlation needs to be further investigated.

In conclusion, DTYMK expression was upregulated and predicted poor prognosis in multiple cancers. At the same time, DTYMK influenced the infiltration of B cell, CD8^+^ T cell, CD4^+^ T cell, macrophage, neutrophil and dendritic cells in several cancers. Inhibition of DTYMK expression in cancer cells impeded cell migration *in vitro*. Therefore, DTYMK could be valuable as a marker for prognosis and immune infiltration in human cancers and may act as a valuable therapeutic target in these cancers.

## Data Availability

The original contributions presented in the study are included in the article/Supplementary Material, further inquiries can be directed to the corresponding authors.

## Supplementary Material

Supplementary Figure S1 The workflow of DTYMK analysis.

Supplementary Figure S2
**(A,B)** Immune infiltration analysis by TIMER.

Supplementary File S1 DTYMK correlated with immune infiltration by TISIDB.

Supplementary File S2 DTYMK interactive gene list obtained from STRING.

Supplementary File S3 KEGG enrichment using DAVID database.

Supplementary File S4 Gene ontology-biological process using DAVID database.

Supplementary File S5 Gene ontology-Cellular component using DAVID database.

Supplementary File S6 Gene ontology-Molecular function using DAVID database.

Supplementary File S7 DTYMK coexpression gene list in LIHC by Linkedomics.

Supplementary File S8 DTYMK coexpression gene list in LUAD by Linkedomics.

Supplementary File S9 DTYMK correlated with prognosis by prognoscan database.

Supplementary Table S1 Comparison of DTYMK expression between cancers and normal tissues using Oncomine.

## Glossary

ACC: Adrenocortical carcinoma
AZT: 3′-azido-3′-deoxythymidine
BLCA: Bladder urothelial carcinoma
BRCA: Breast invasive carcinoma
CESC: Cervical squamous cell carcinoma and endocervical adenocarcinoma
CHOL: Cholangio carcinoma
COAD: Colon adenocarcinoma
CPTAC: Clinical Proteomic Tumor Analysis Consortium
DAVID: The Database for Annotation, Visualization and Integrated Discovery
DLBC: Lymphoid neoplasm diffuse large B-cell lymphoma
dNTP: deoxyribonucleoside triphosphates
dTMP: deoxythymidine monophosphate
dTDP: deoxythymidine diphosphate
DTYMK: deoxythymidylate kinase
ESCA: Esophageal carcinoma
GBM: Glioblastoma multiforme
GEPIA: Gene Expression Profiling Interactive Analysis
GSEA: Gene Set Enrichment Analysis
HNSC: Head and neck squamous cell carcinoma
KICH: Kidney chromophobe
KIRC: Kidney renal clear cell carcinoma
KIRP: Kidney renal papillary cell carcinoma
LAML: Acute myeloid leukemia
LGG: Brain lower grade glioma
LIHC: Liver hepatocellular carcinoma
LKB1: serine/threonine kinase 11
LUAD: Lung adenocarcinoma
LUSC: Lung squamous cell carcinoma
MDDS: mitochondrial DNA depletion syndrome
MESO: Mesothelioma
mTOR: mechanistic target of rapamycin kinase
MYC: v-myc avian myelocytomatosis viral oncogene homolog
OV: Ovarian serous cystadenocarcinoma
PAAD: Pancreatic adenocarcinoma
PCPG: pheochromocytoma and paraganglioma
PRAD: Prostate adenocarcinoma
PVRL2: Poliovirus receptor-related 2
PyM: Pyrimidine metabolism
READ: Rectum adenocarcinoma
SARC: Sarcoma
SKCM: Skin cutaneous melanoma
STAD: Stomach adenocarcinoma
TAMs: tumor-associated macrophages
TCGA: The Cancer Genome Atlas
TGCT: Testicular germ cell tumors
THCA: Thyroid carcinoma
THYM: Thymoma
TIGIT: T cell immunoreceptor with Ig and ITIM domains
TIMER: Tumor IMmune Estimation Resource database
TMB: tumor mutation burden
TME: tumor microenvironment
UCEC: Uterine corpus endometrial carcinoma
UCS: Uterine carcinosarcoma
UVM: Uveal melanoma
