# Supplementary material for: Deoxythymidylate Kinase as a Promising Marker for Predicting Prognosis and Immune Cell Infiltration of Pan-cancer
Source: Front Mol Biosci. 2022 Jul 12;9:887059. doi: 10.3389/fmolb.2022.887059 (PMC9315941; doi:10.3389/fmolb.2022.887059)
Supplement: Supplementary file 1 [file DataSheet1.zip › supplementary files/supplementary file 1.pdf]

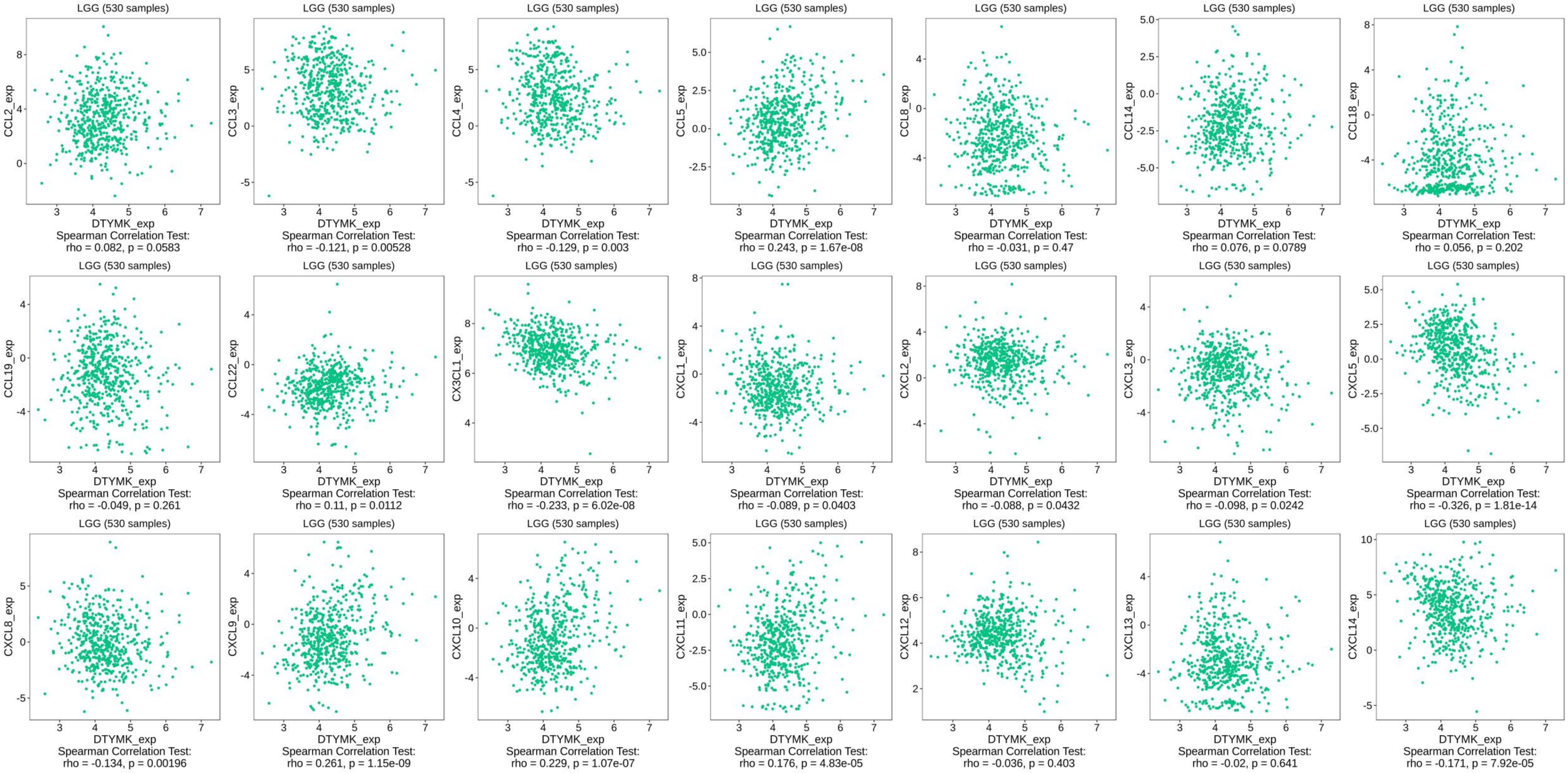

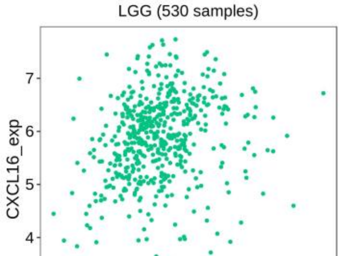

Correlation between DTYMK expression and chemokines expression in LGG

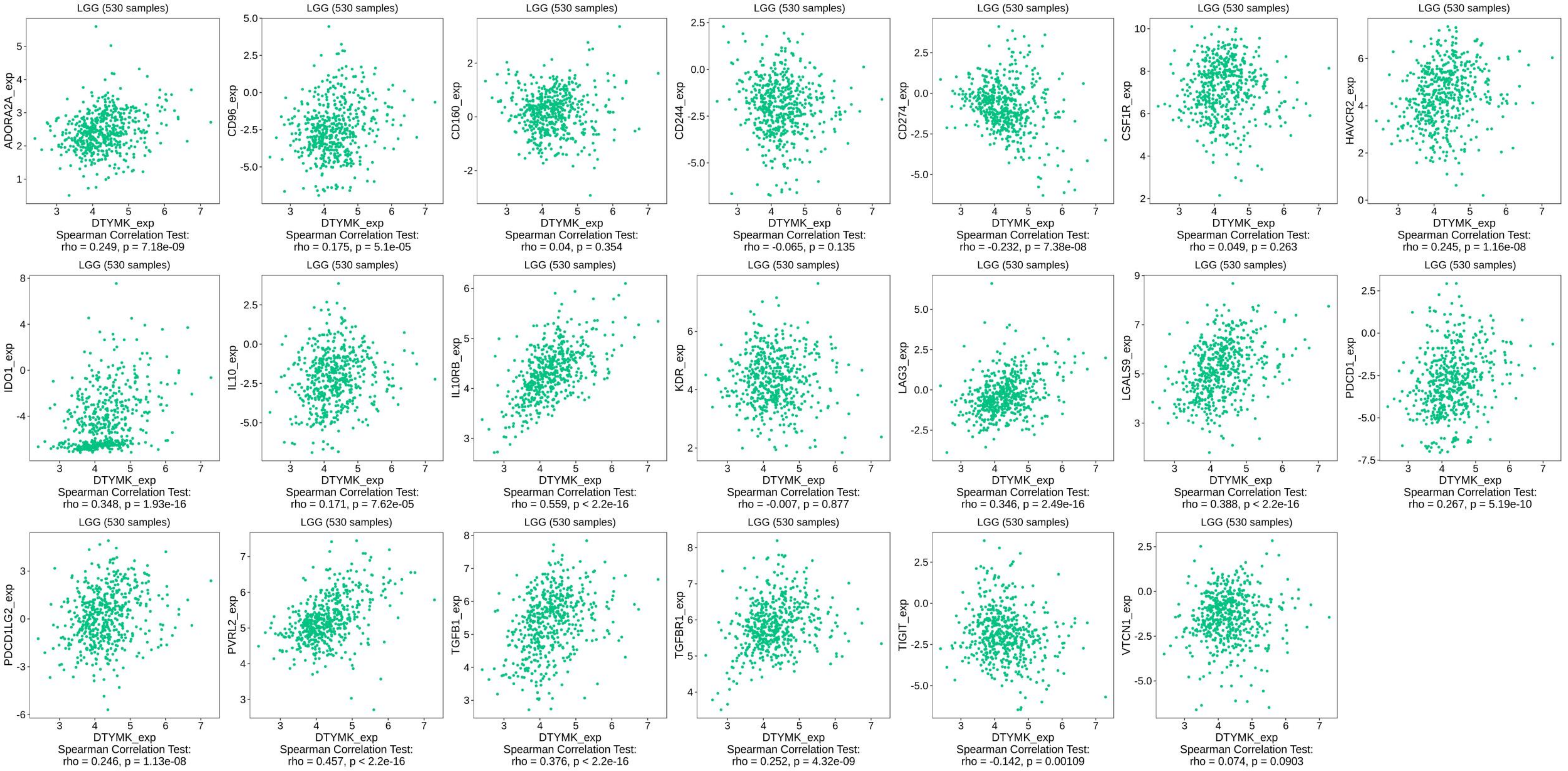

Correlation between DTYMK expression and immunoinhibitors expression in LGG

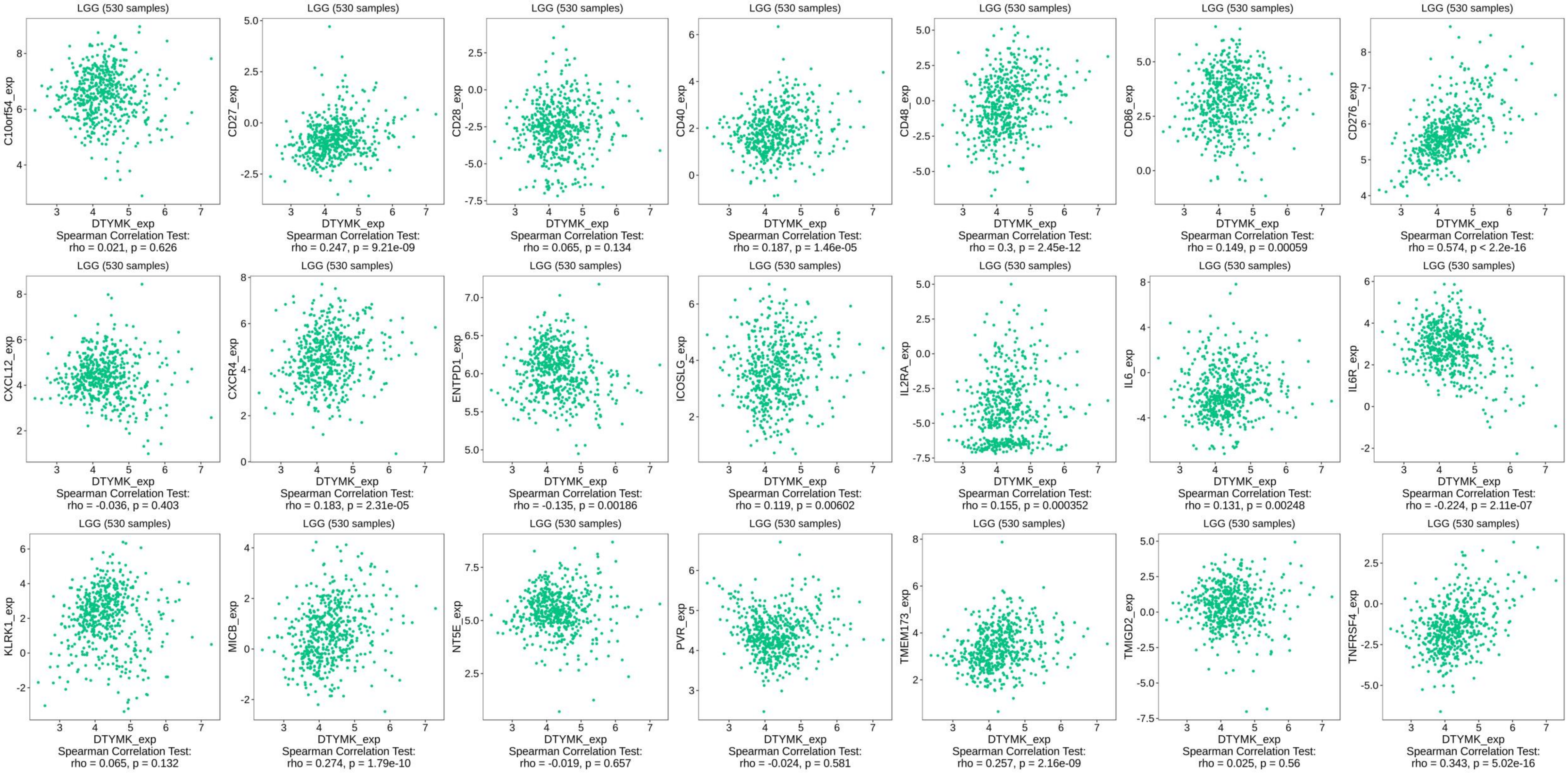

Correlation between DTYMK expression and immunostimulators expression in LGG

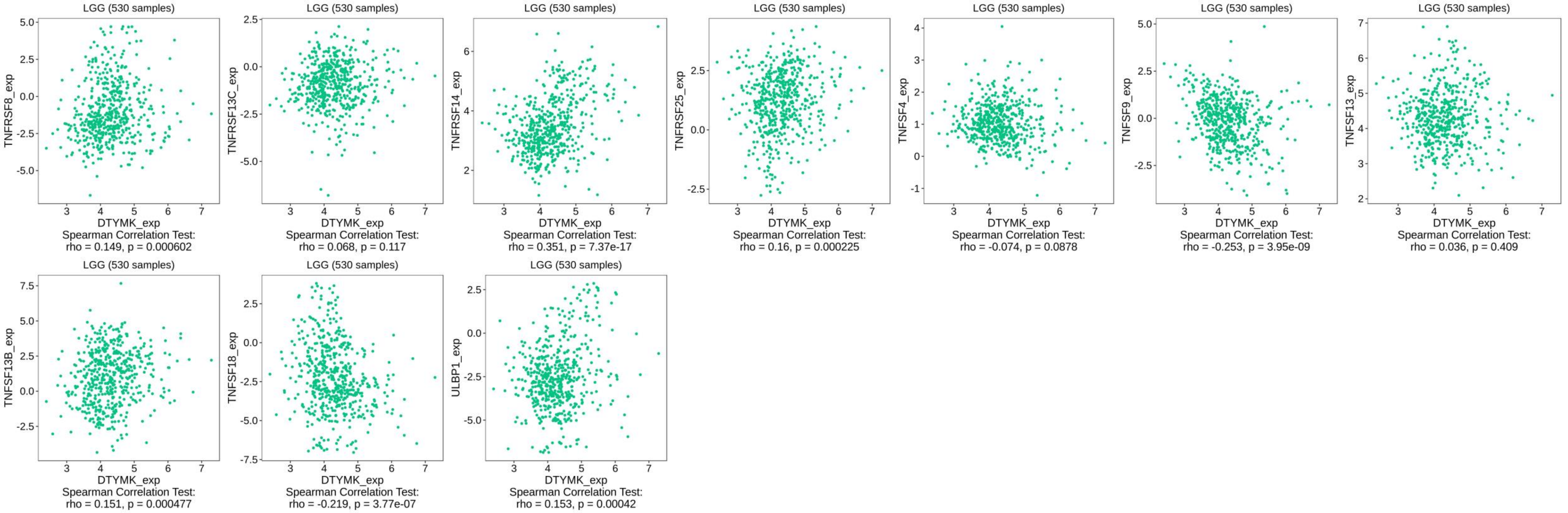

Correlation between DTYMK expression and immunostimulators expression in LGG

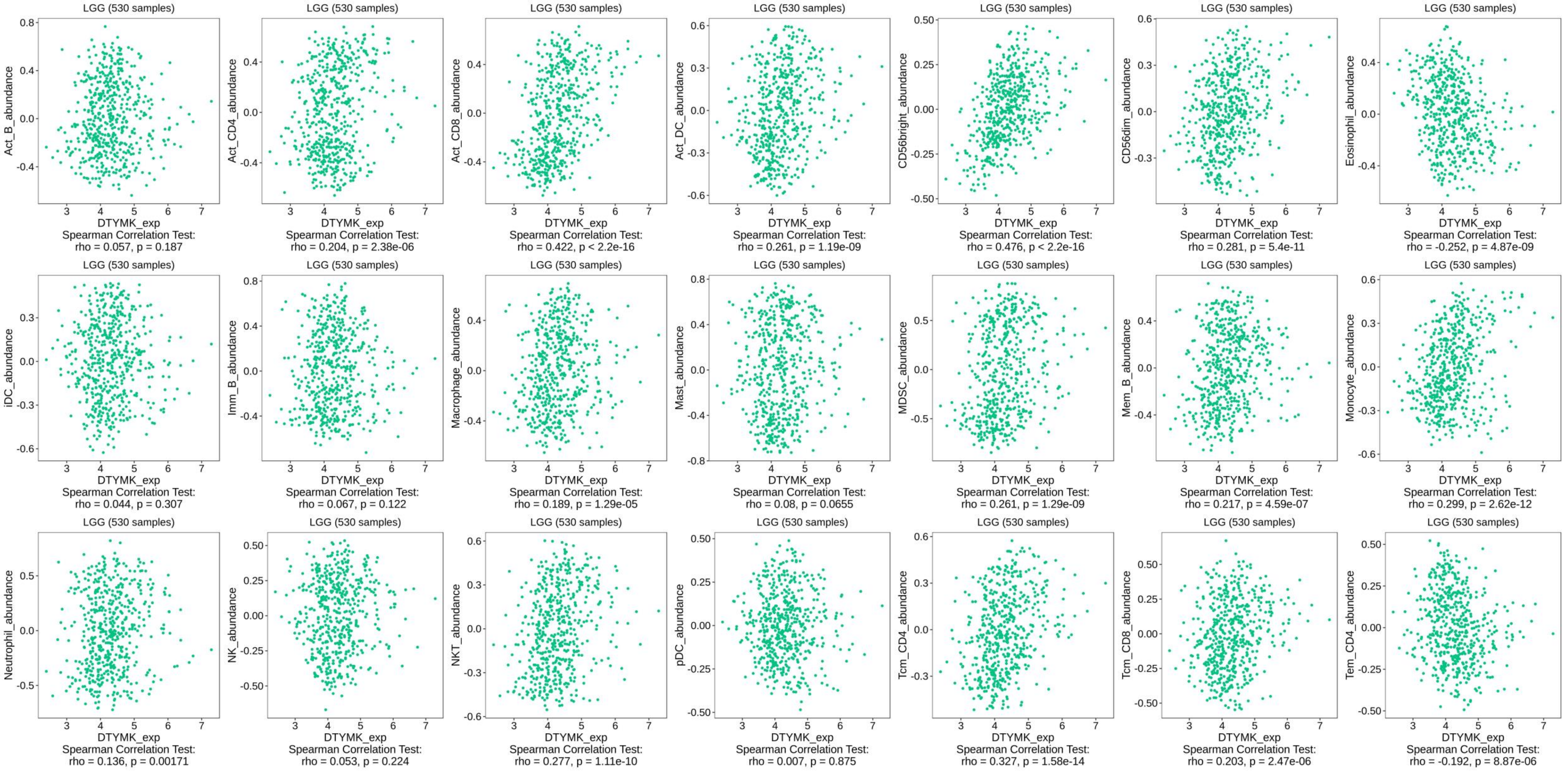

Correlation between DTYMK expression and lymphocyte infiltration in LGG

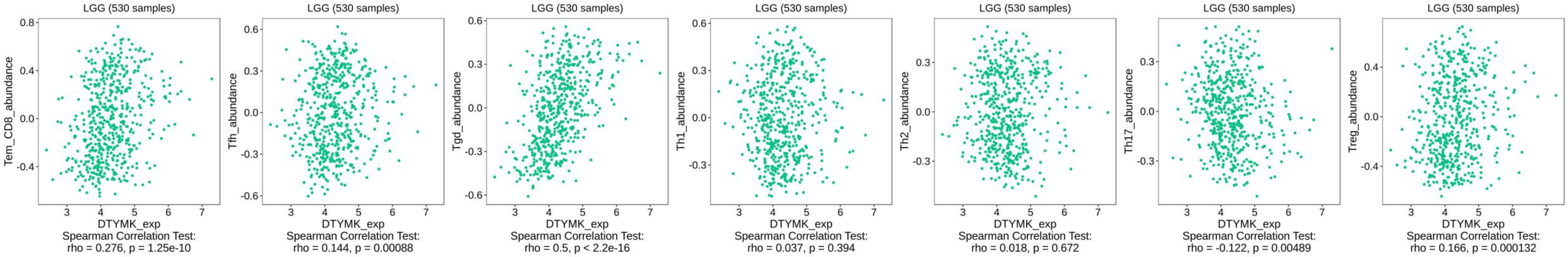

Correlation between DTYMK expression and lymphocyte infiltration in LGG

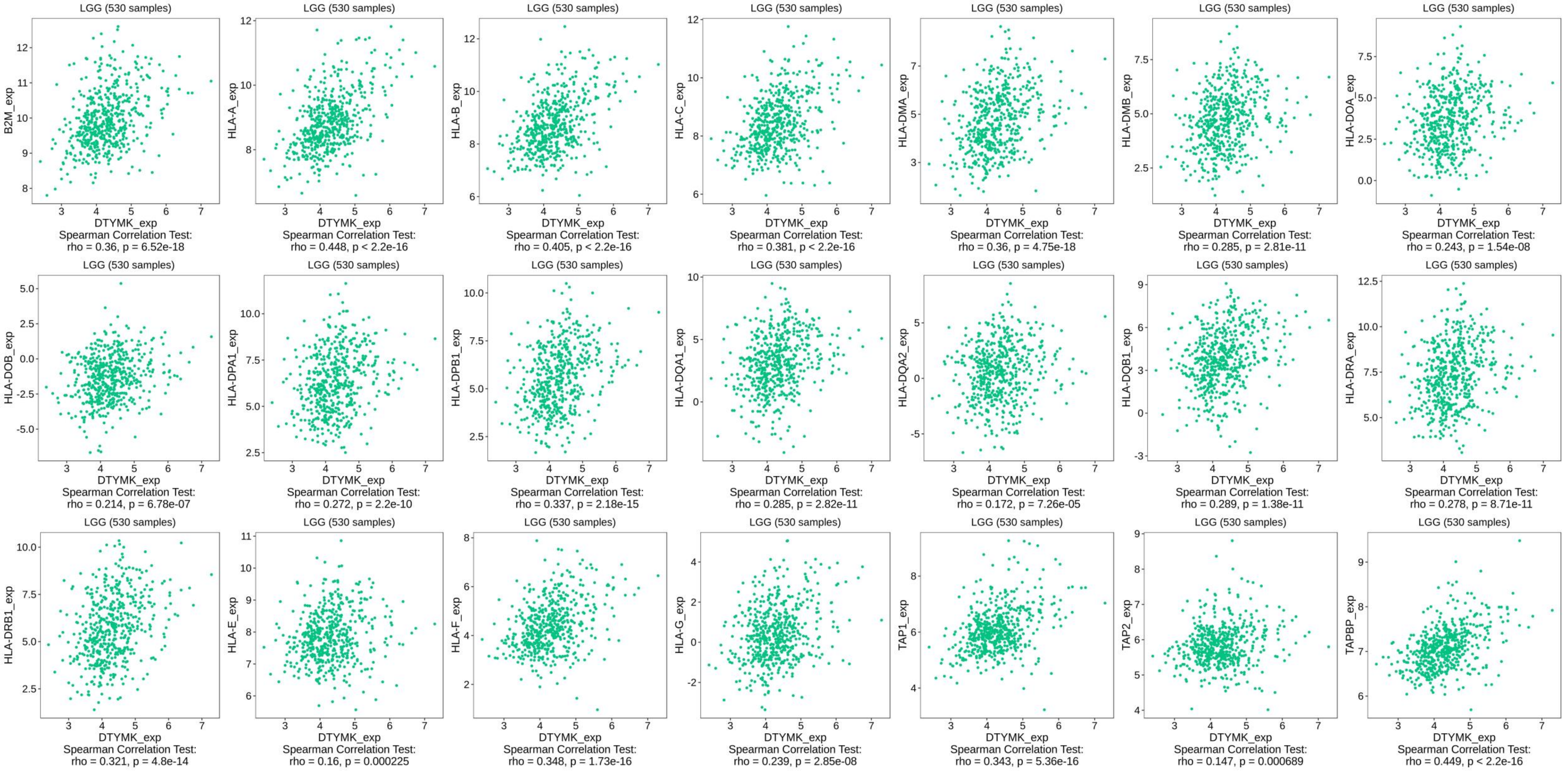

Correlation between DTYMK expression and MHC expression in LGG

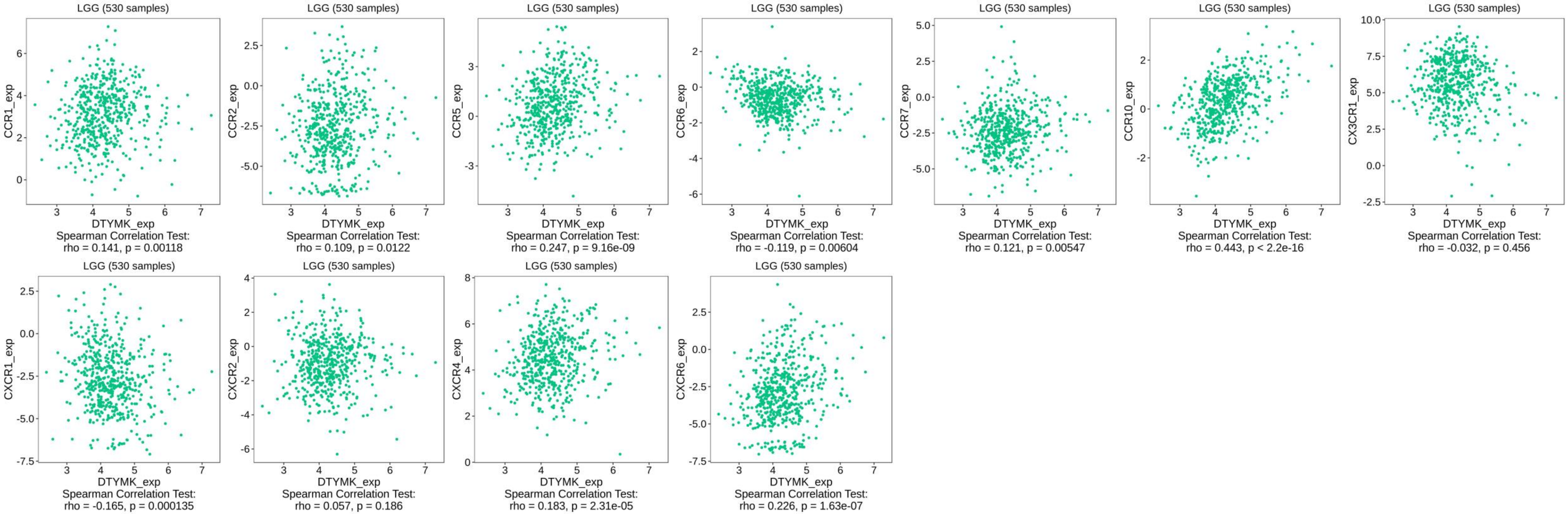

Correlation between DTYMK expression and chemokine receptors in LGG

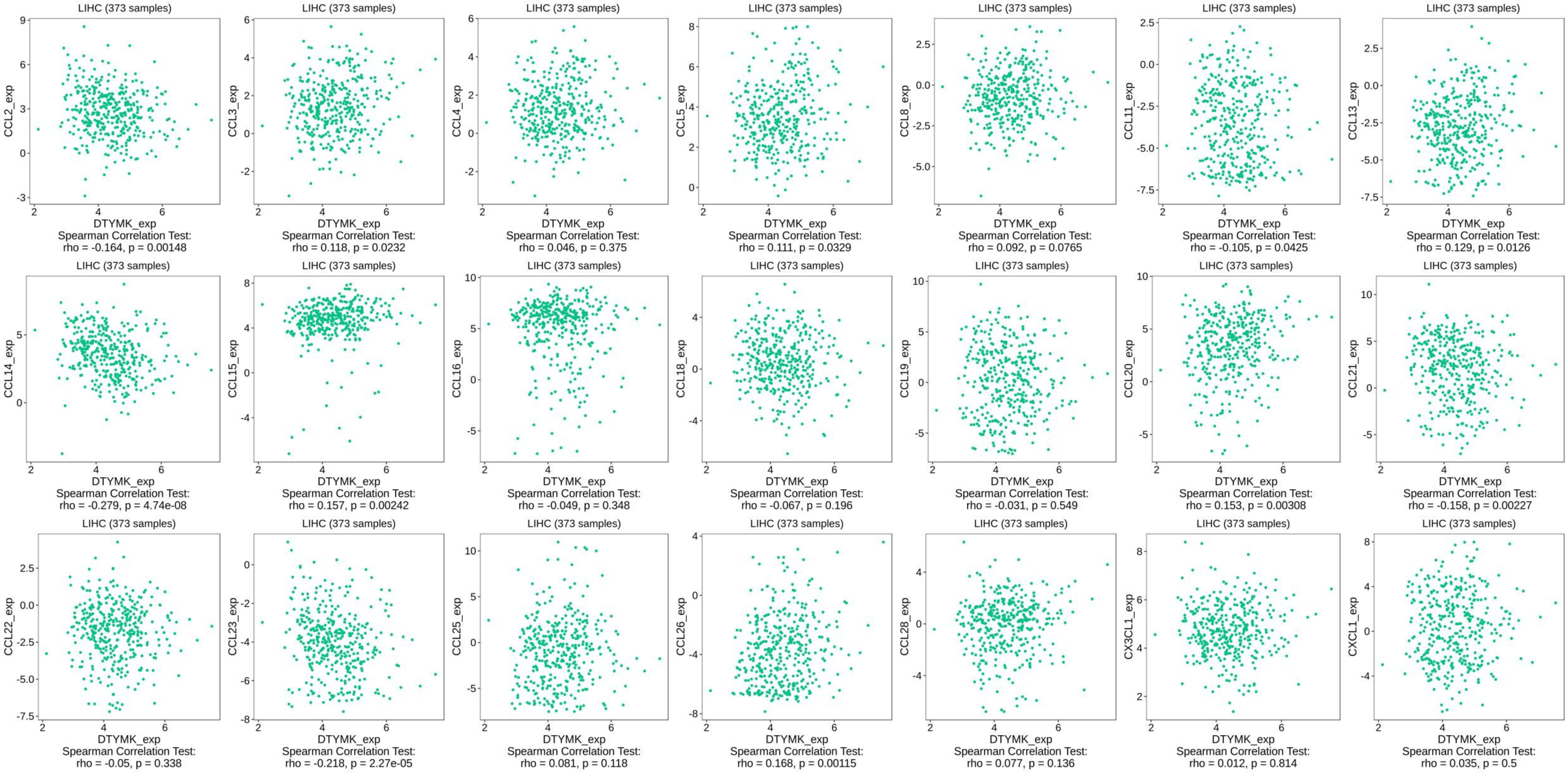

Correlation between DTYMK expression and chemokines expression in LIHC

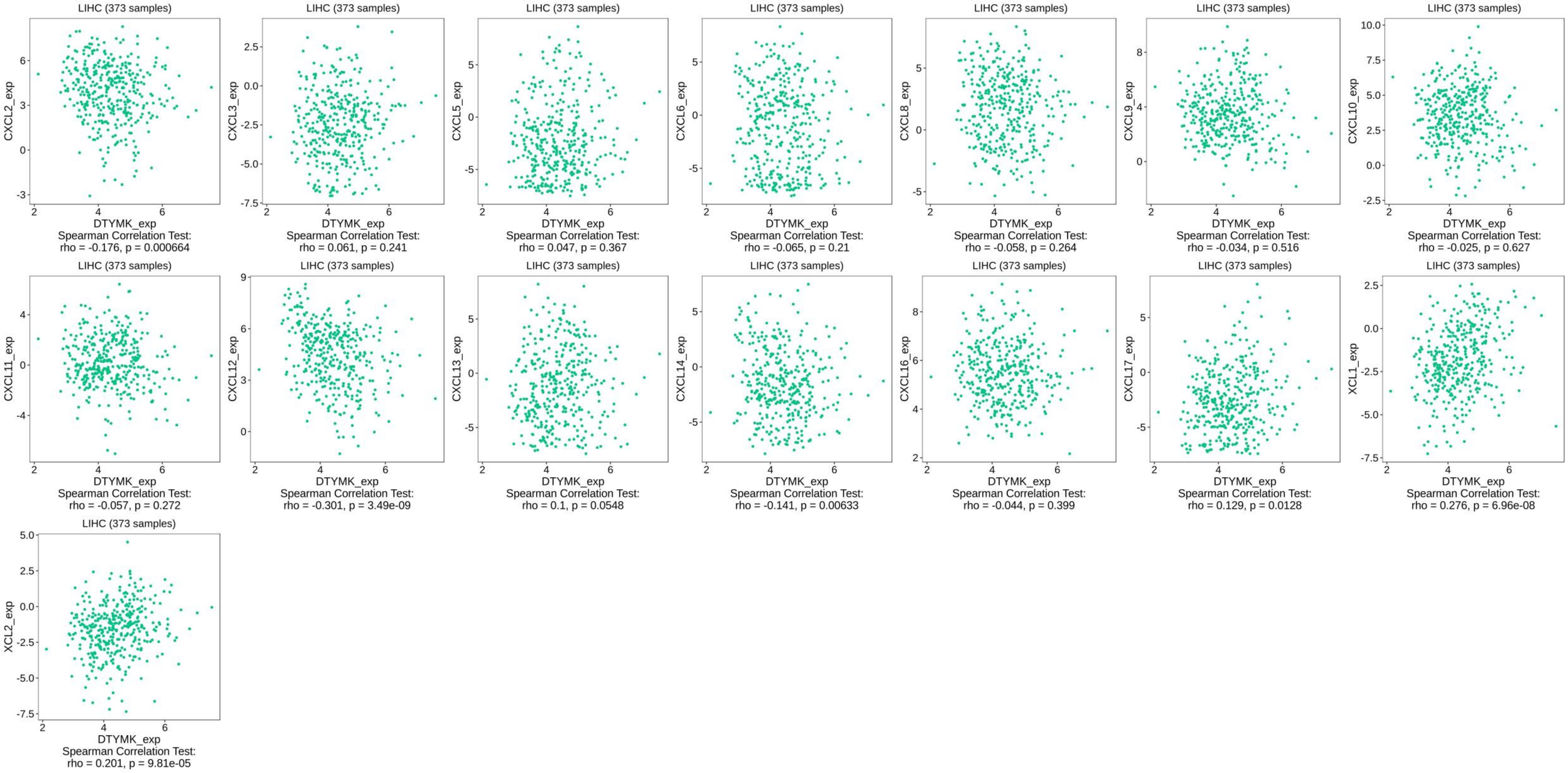

Correlation between DTYMK expression and chemokines expression in LIHC

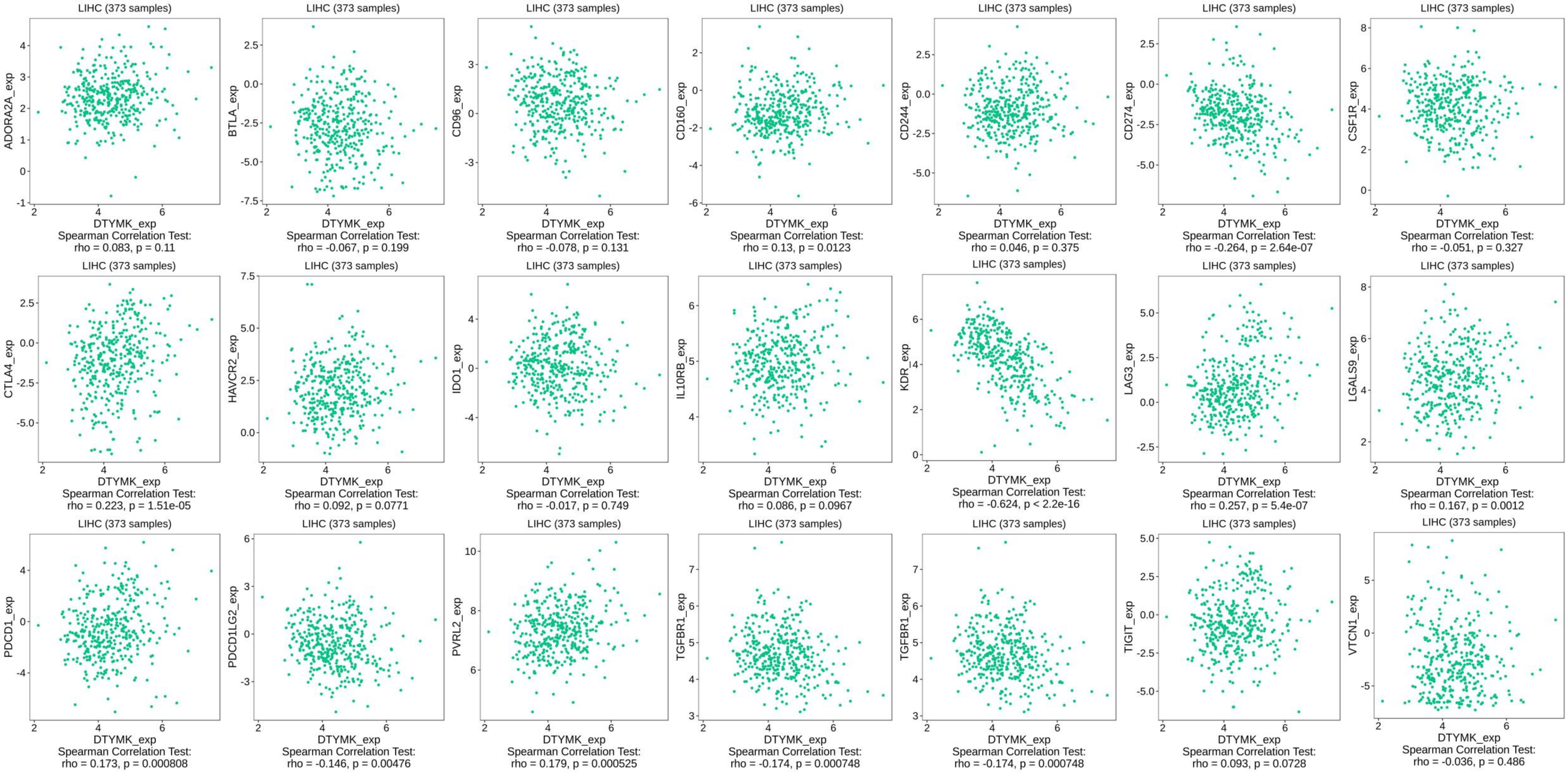

Correlation between DTYMK expression and immunoinhibitors expression in LIHC

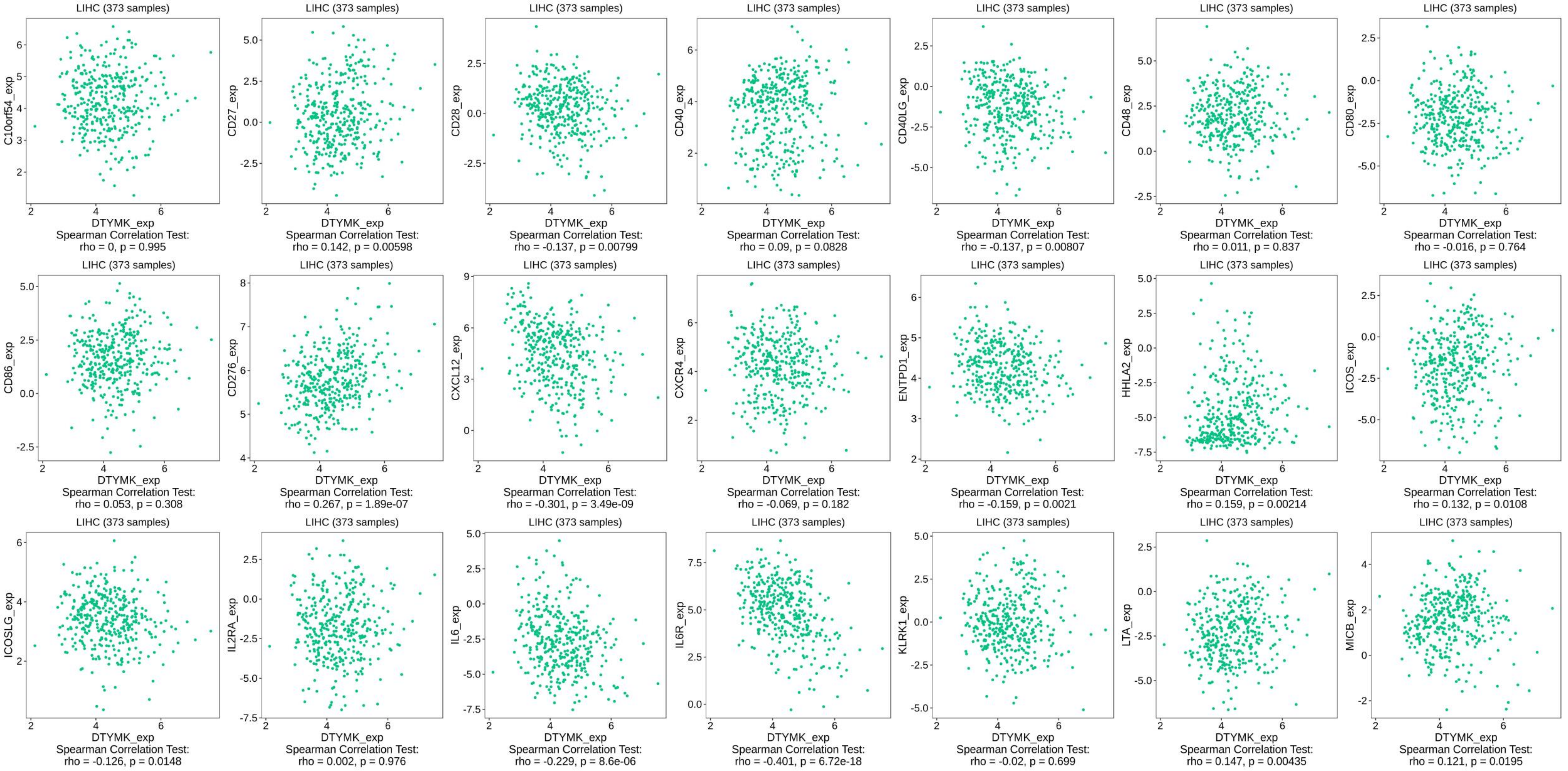

Correlation between DTYMK expression and immunostimulators expression in LIHC

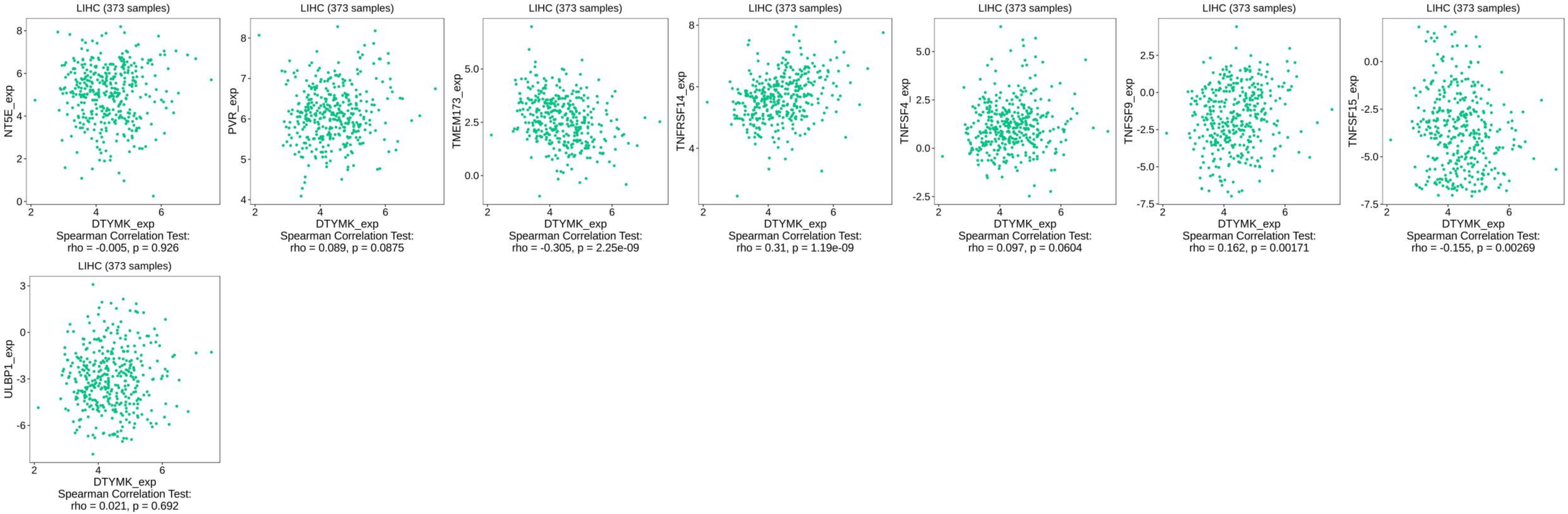

Correlation between DTYMK expression and immunostimulators expression in LIHC

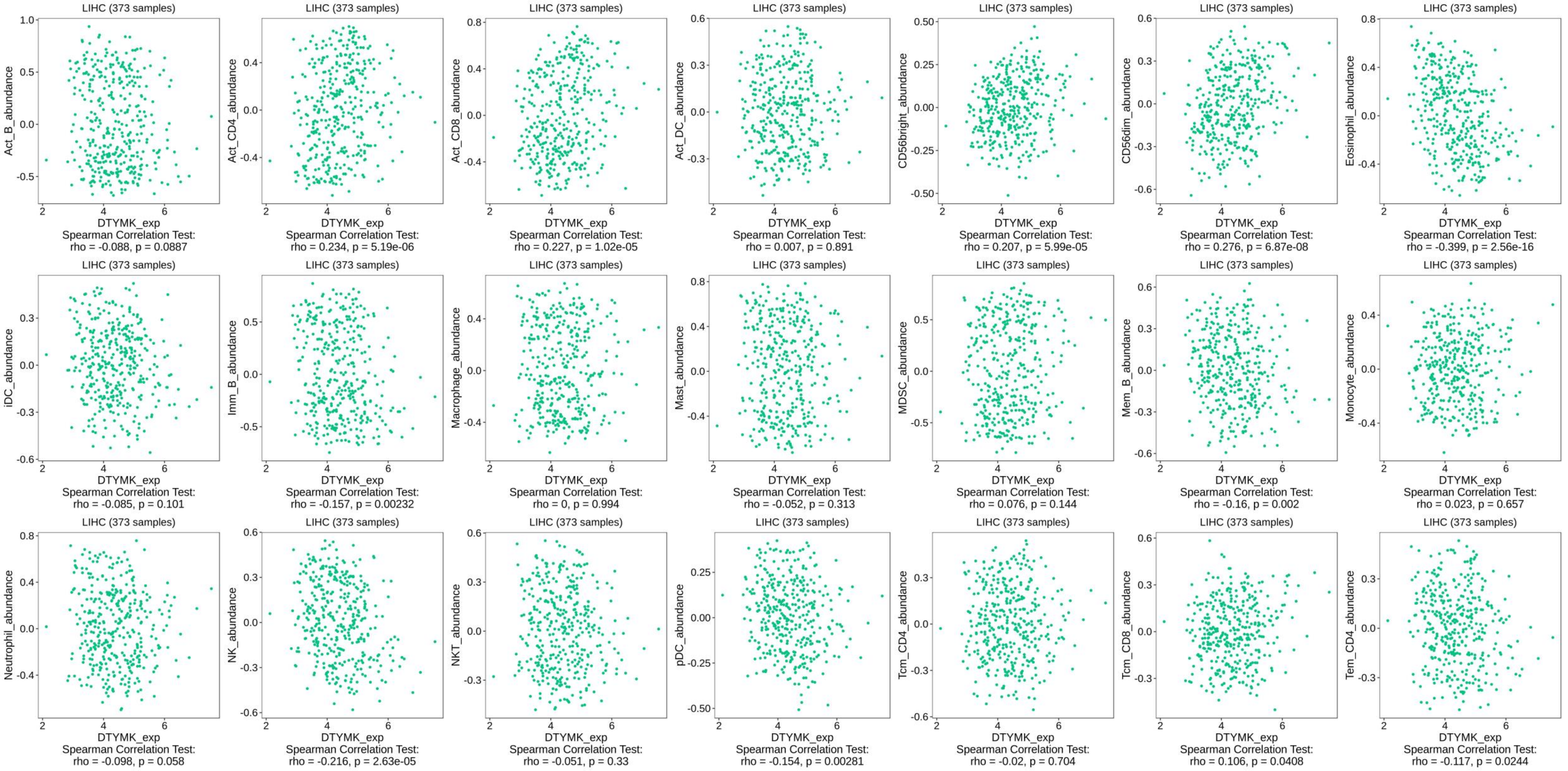

Correlation between DTYMK expression and lymphocytes infiltration in LIHC

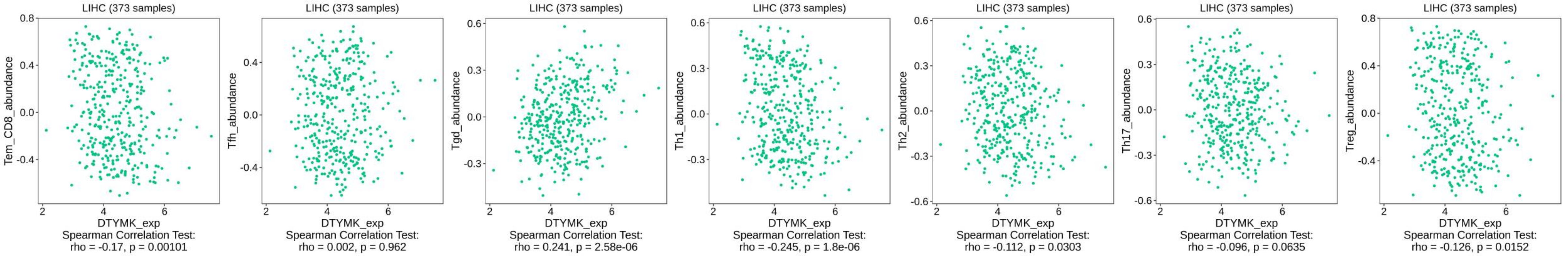

Correlation between DTYMK expression and lymphocytes infiltration in LIHC

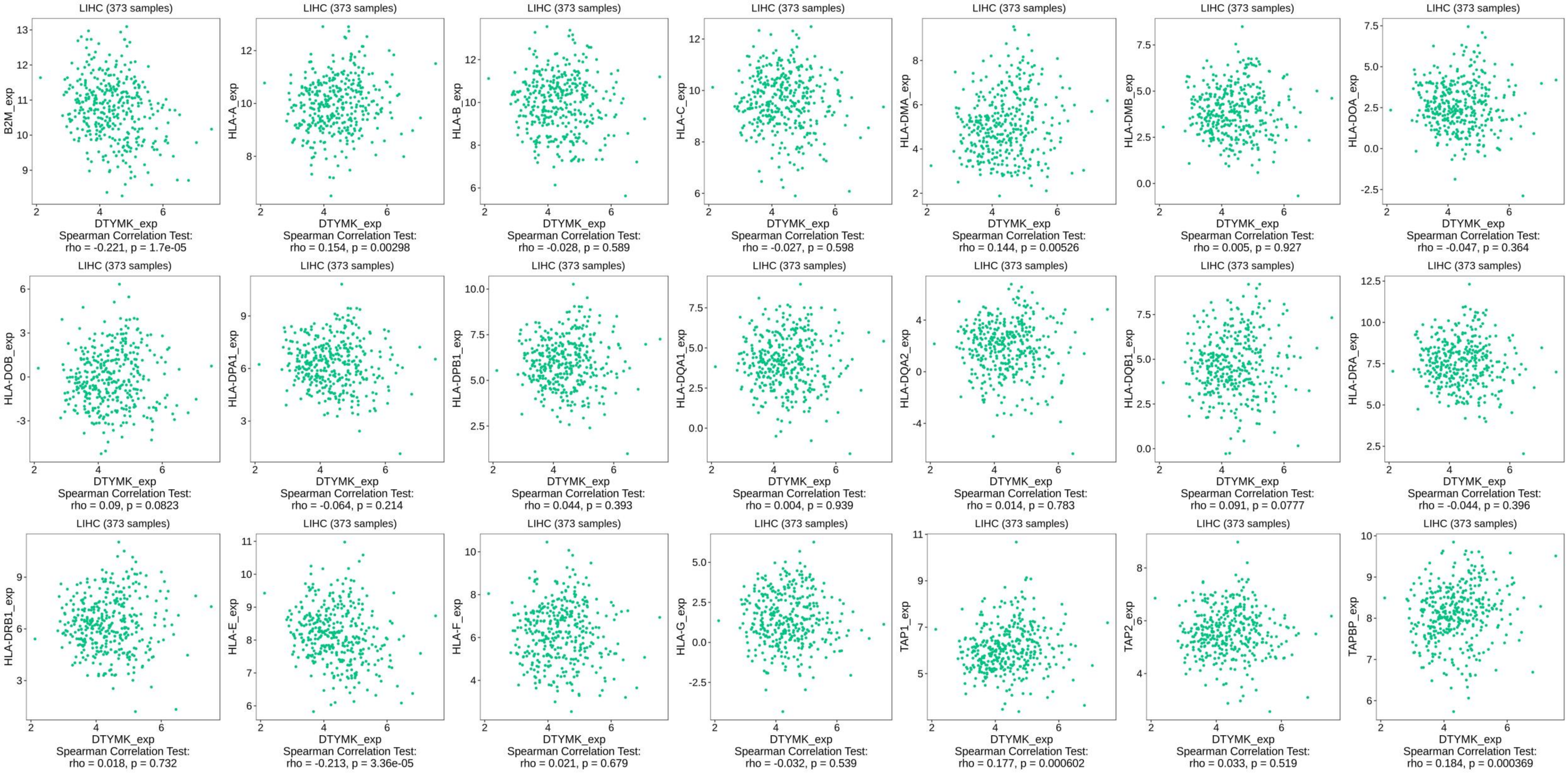

Correlation between DTYMK expression and MHC expression in LIHC

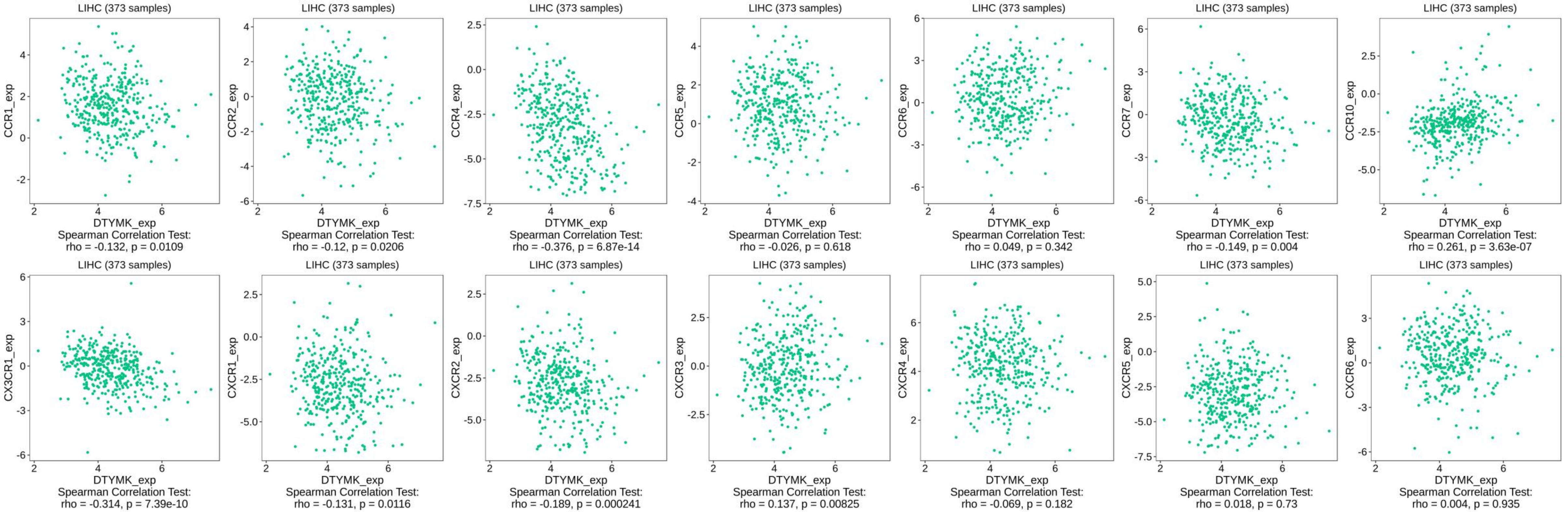

Correlation between DTYMK expression and chemokine receptors expression in LIHC

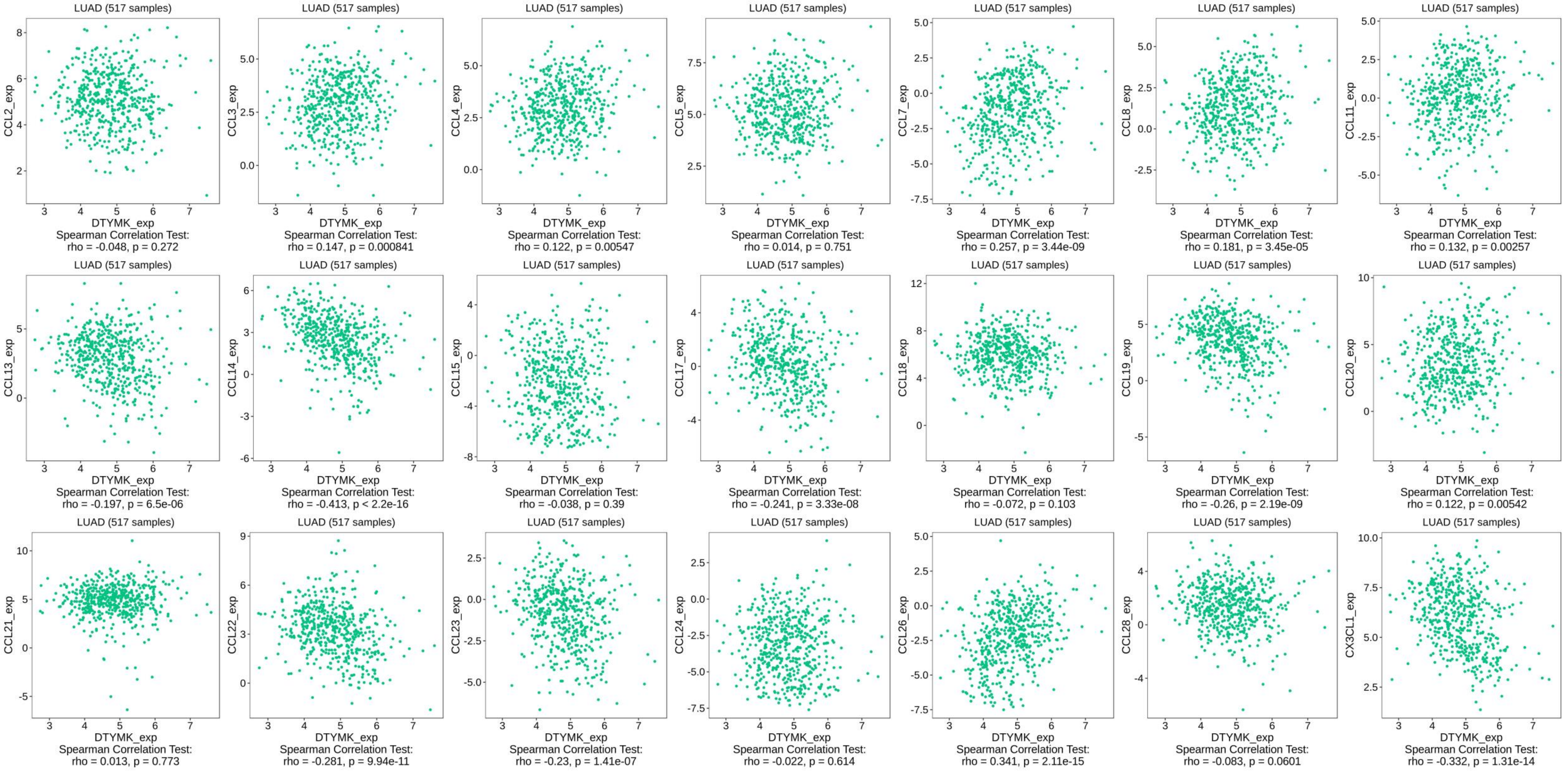

Correlation between DTYMK expression and chemokines expression in LUAD

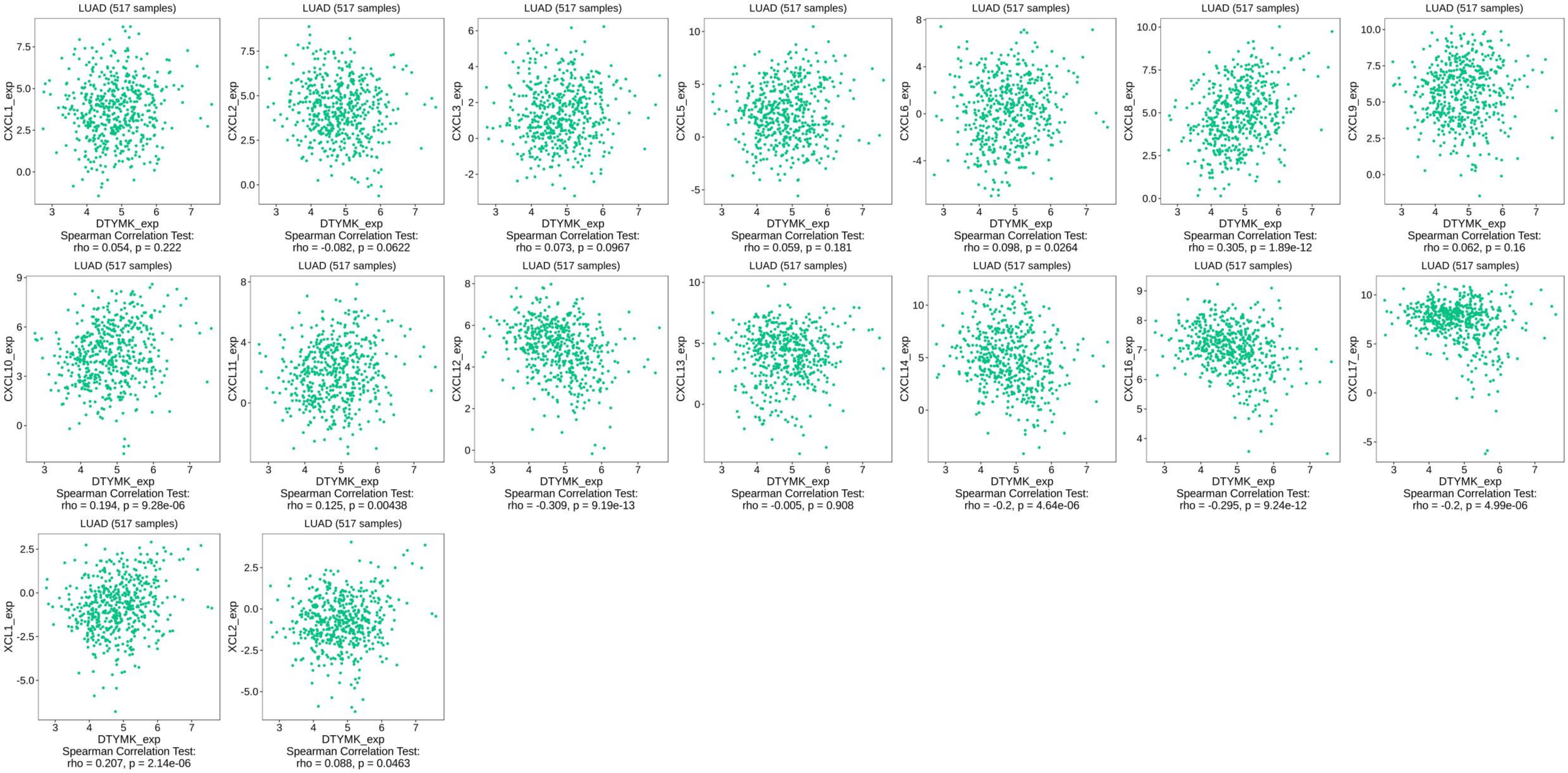

Correlation between DTYMK expression and chemokines expression in LUAD

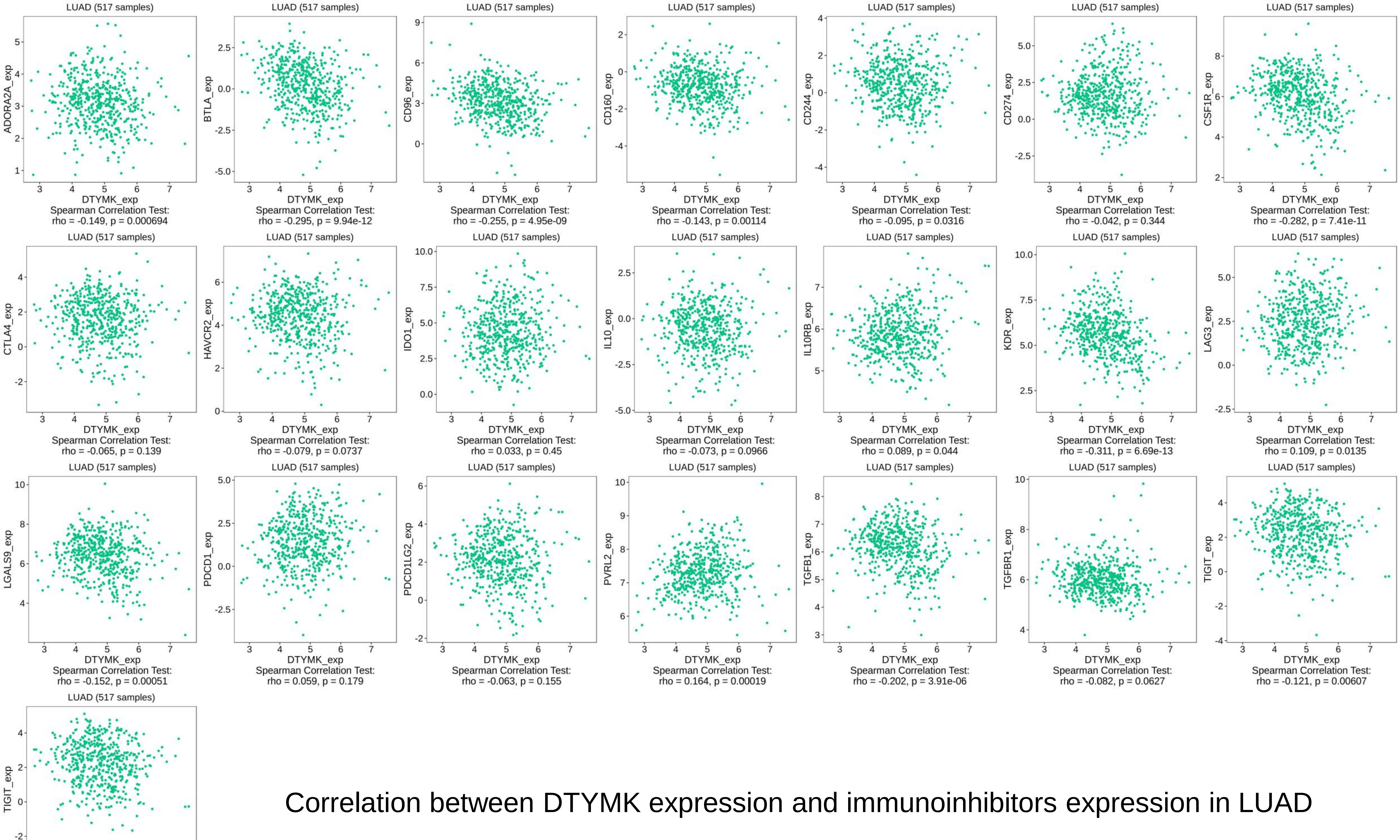

Correlation between DTYMK expression and immunoinhibitors expression in LUAD

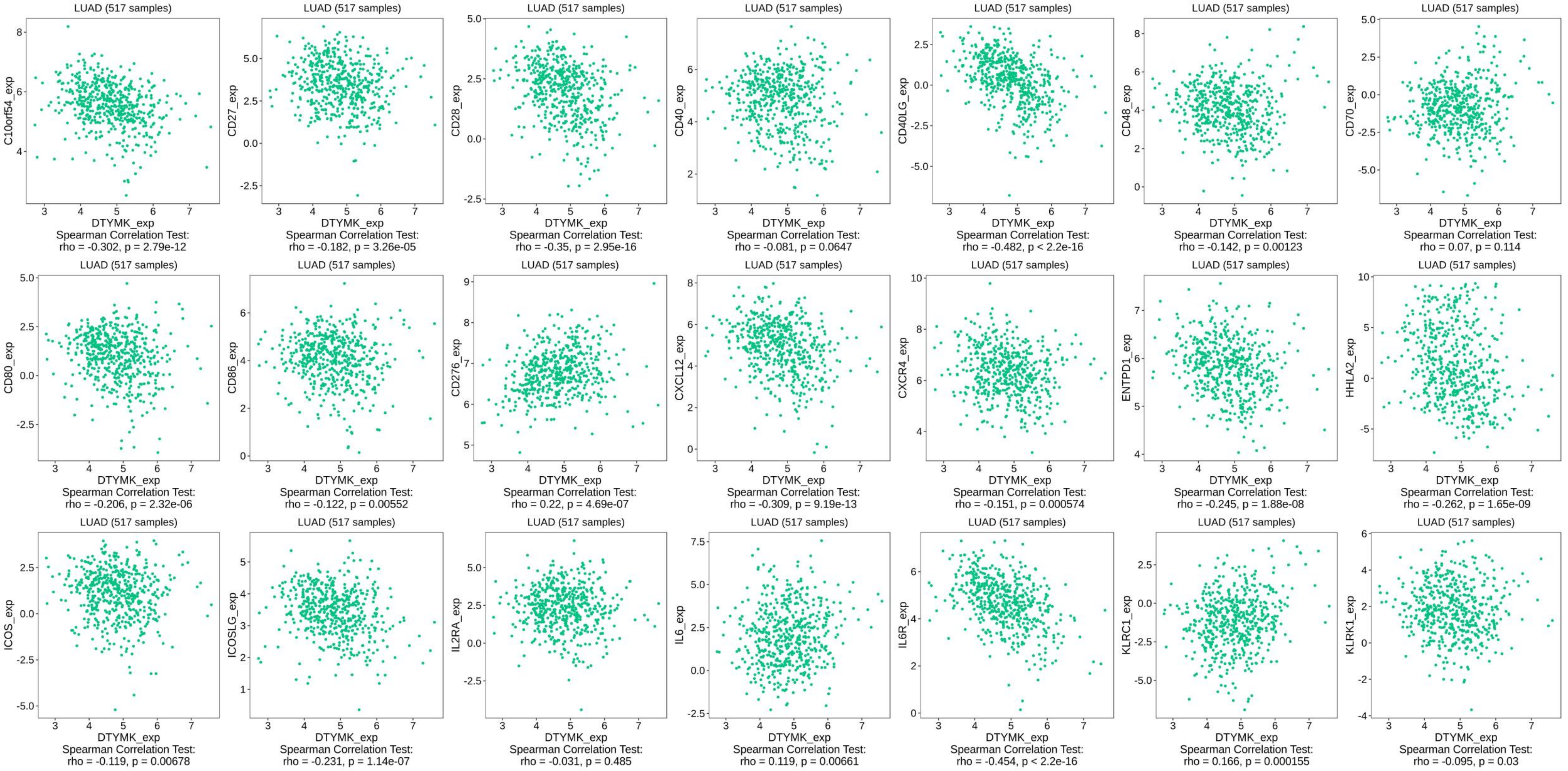

Correlation between DTYMK expression and immunostimulators expression in LUAD

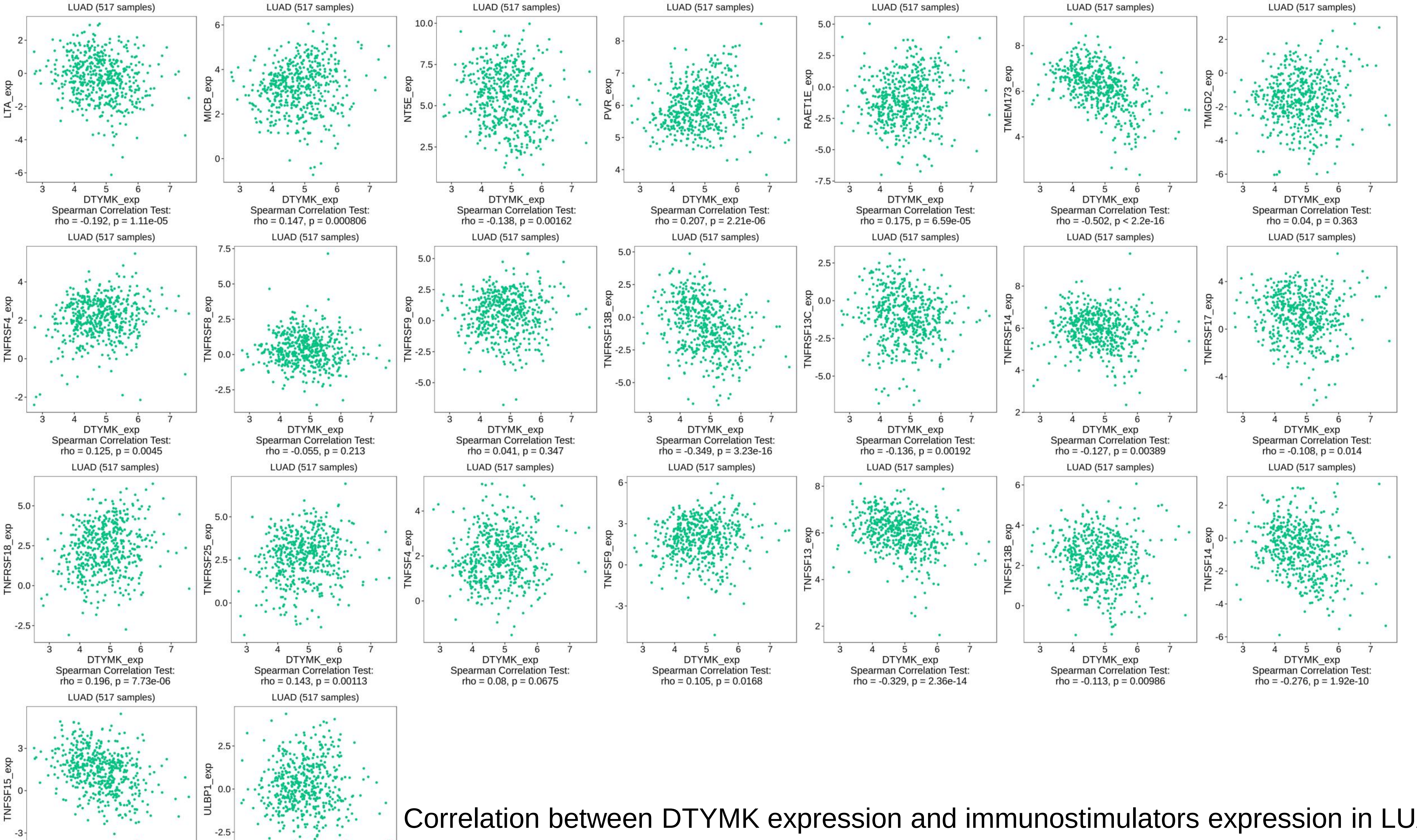

Correlation between DTYMK expression and immunostimulators expression in LUAD

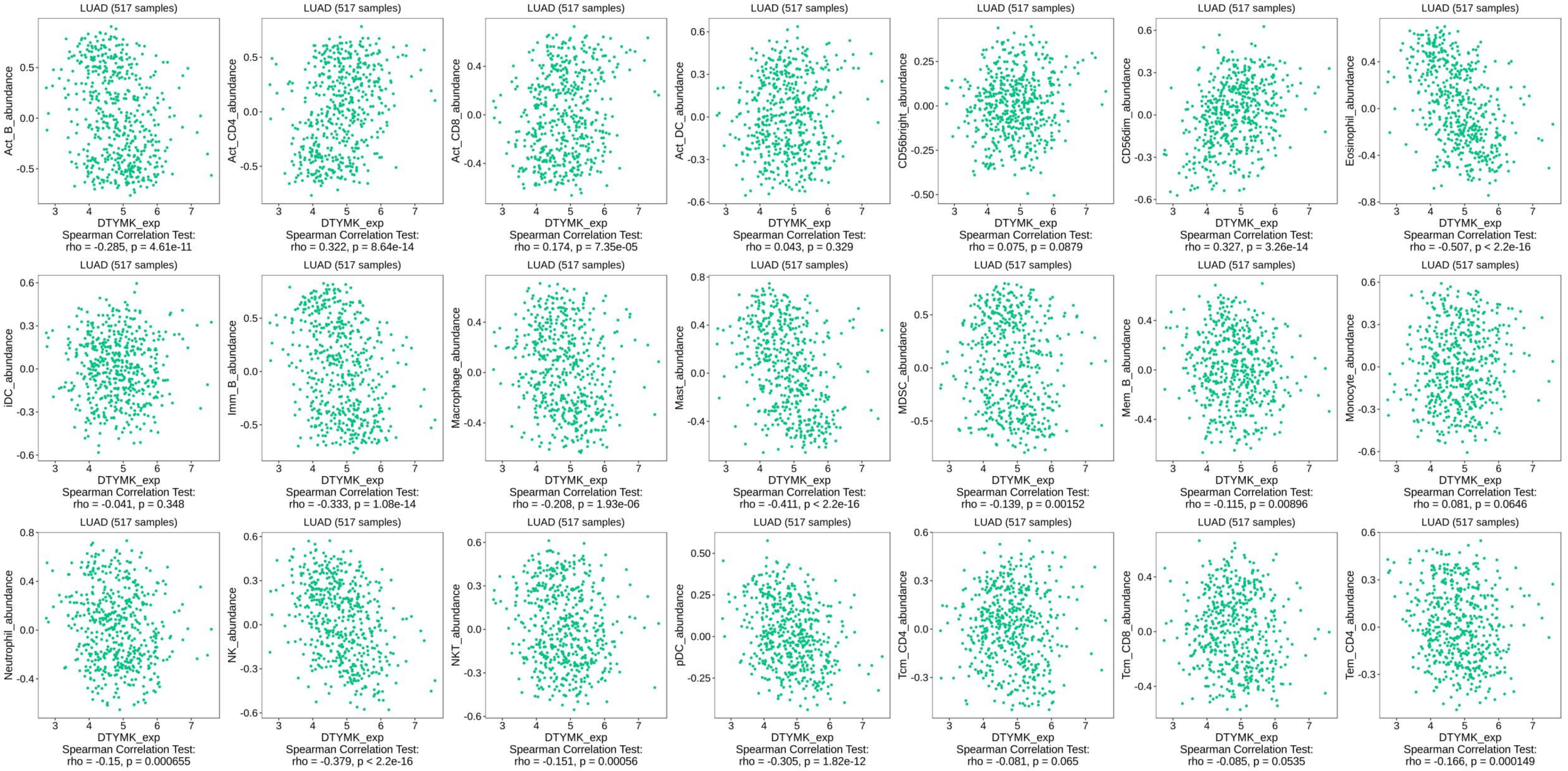

Correlation between DTYMK expression and lymphocytes infiltration in LUAD

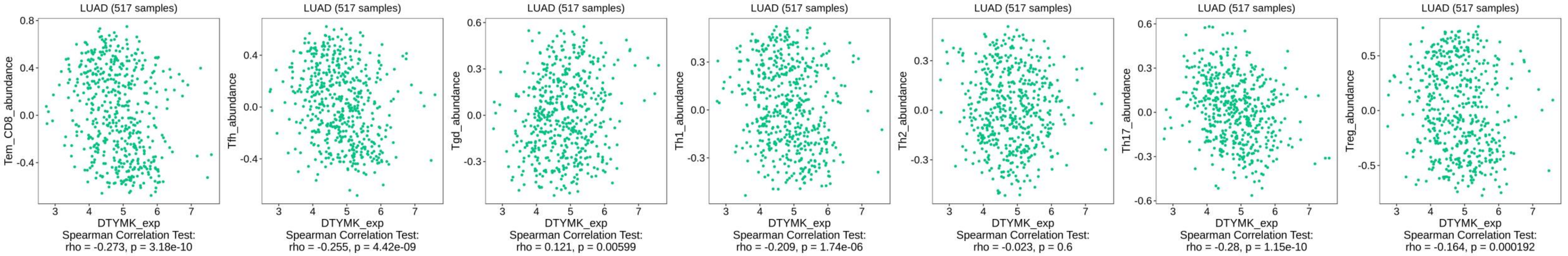

Correlation between DTYMK expression and lymphocytes infiltration in LUAD

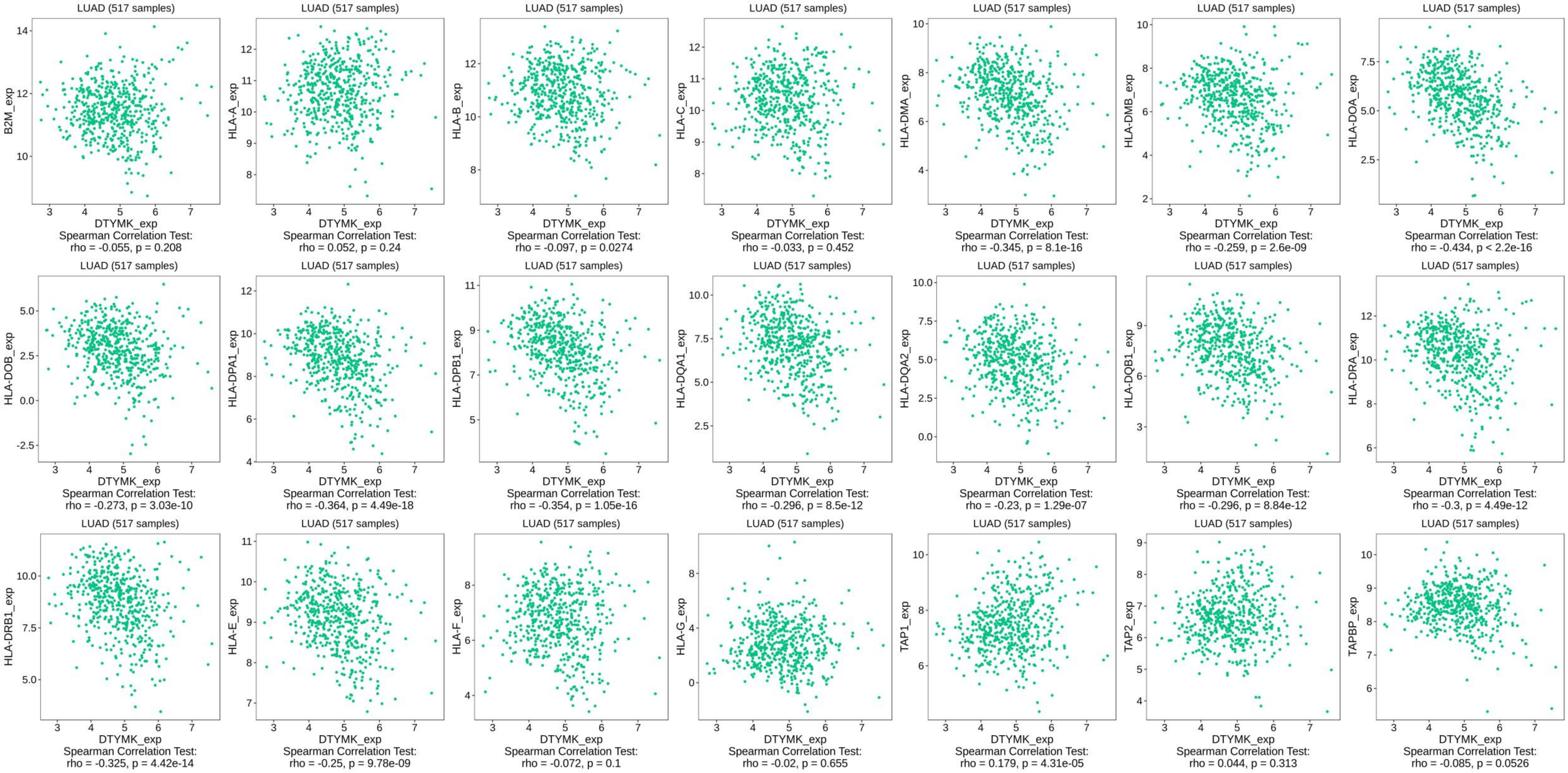

Correlation between DTYMK expression and MHC expression in LUAD

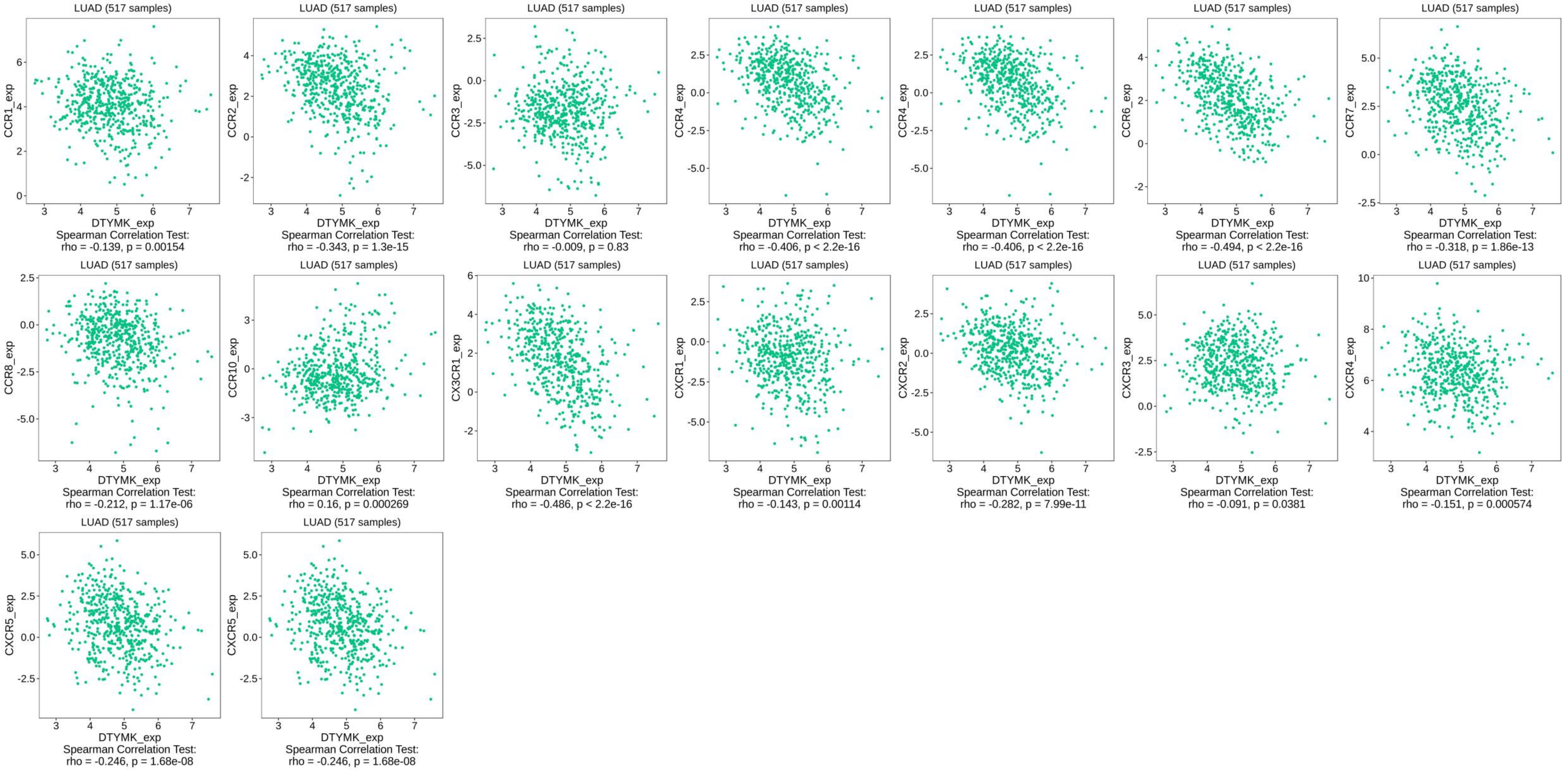

Correlation between DTYMK expression and chemokine receptors expression in LUAD

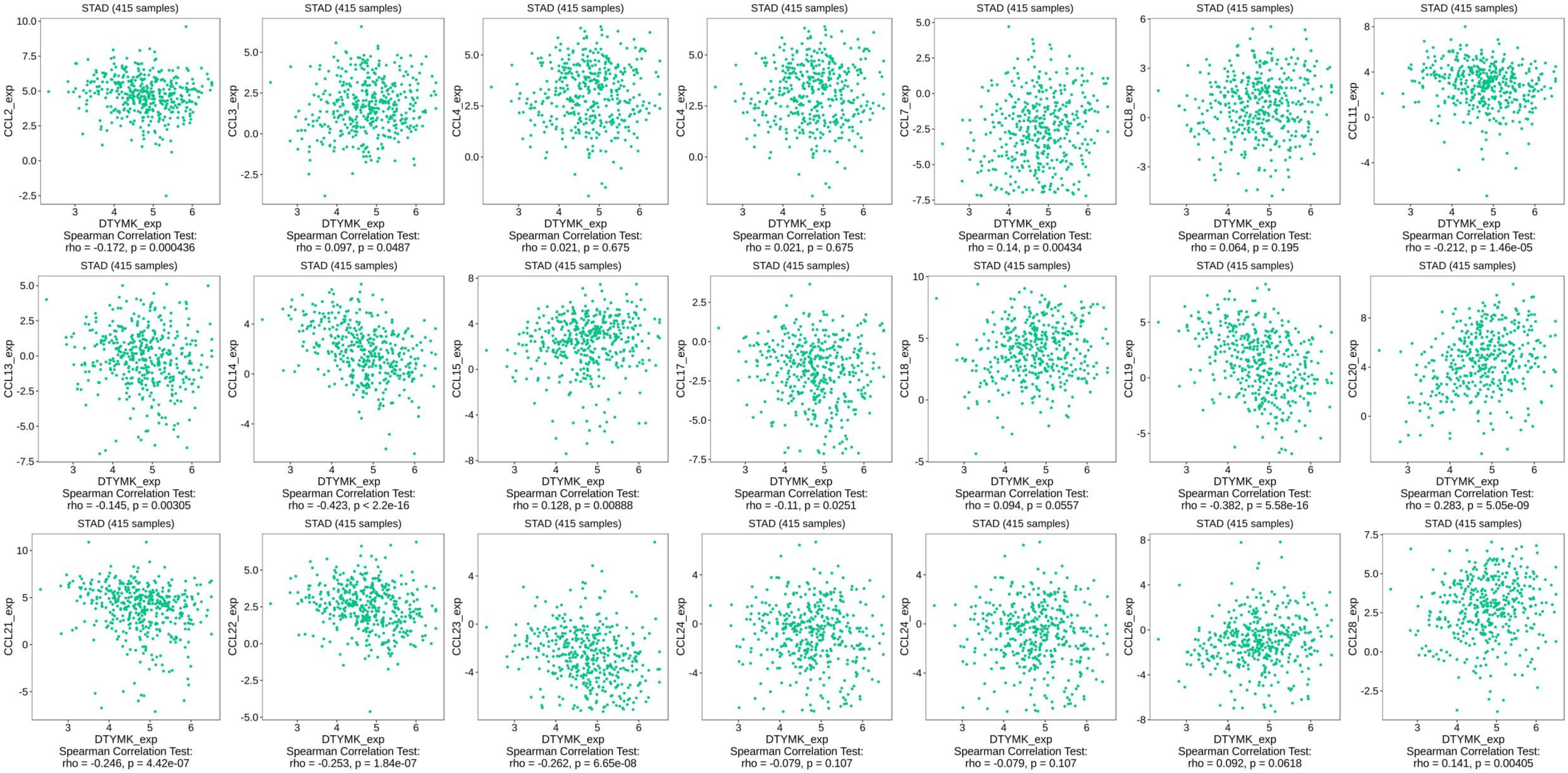

Correlation between DTYMK expression and chemokines expression in STAD

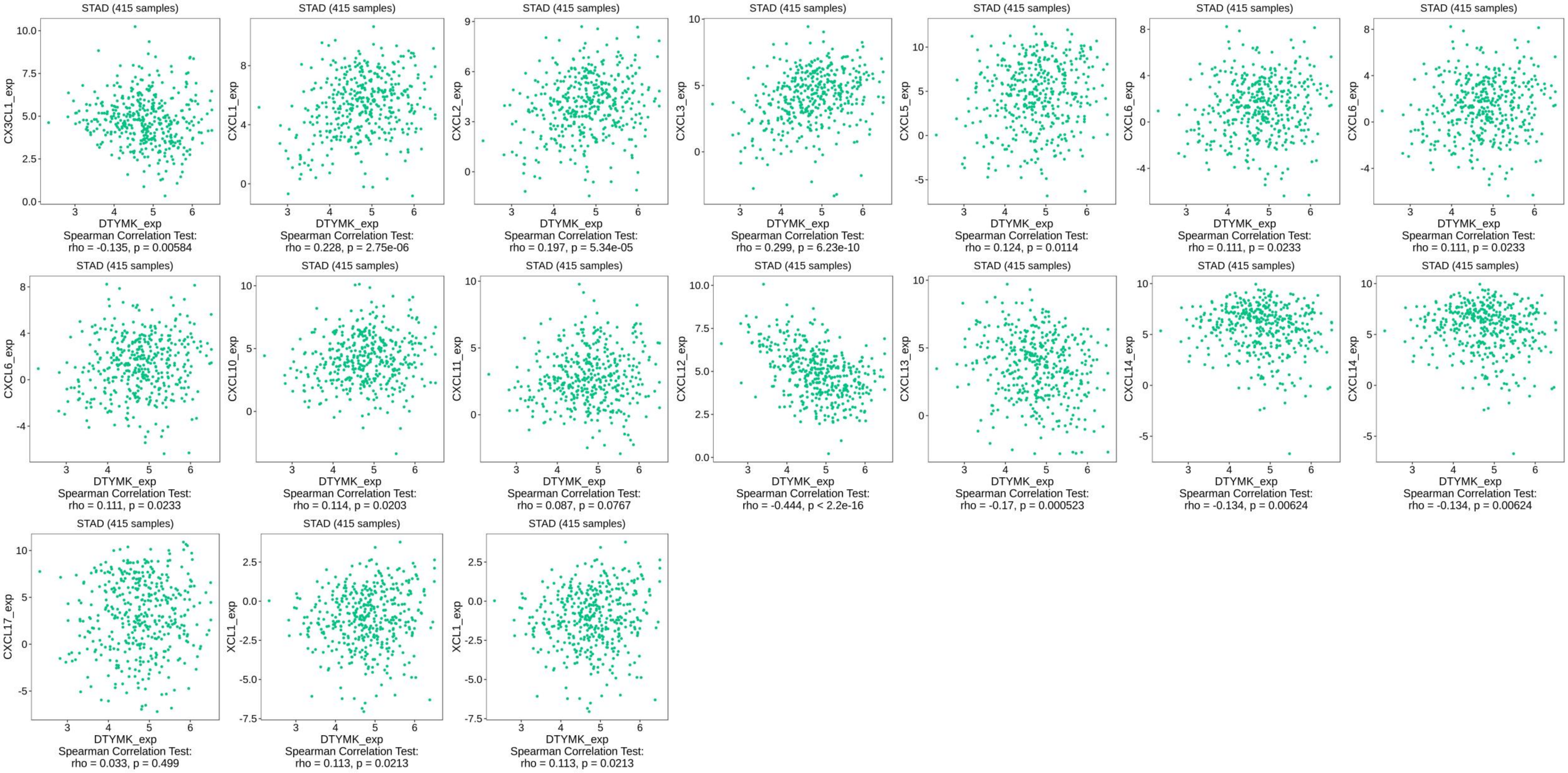

Correlation between DTYMK expression and chemokines expression in STAD

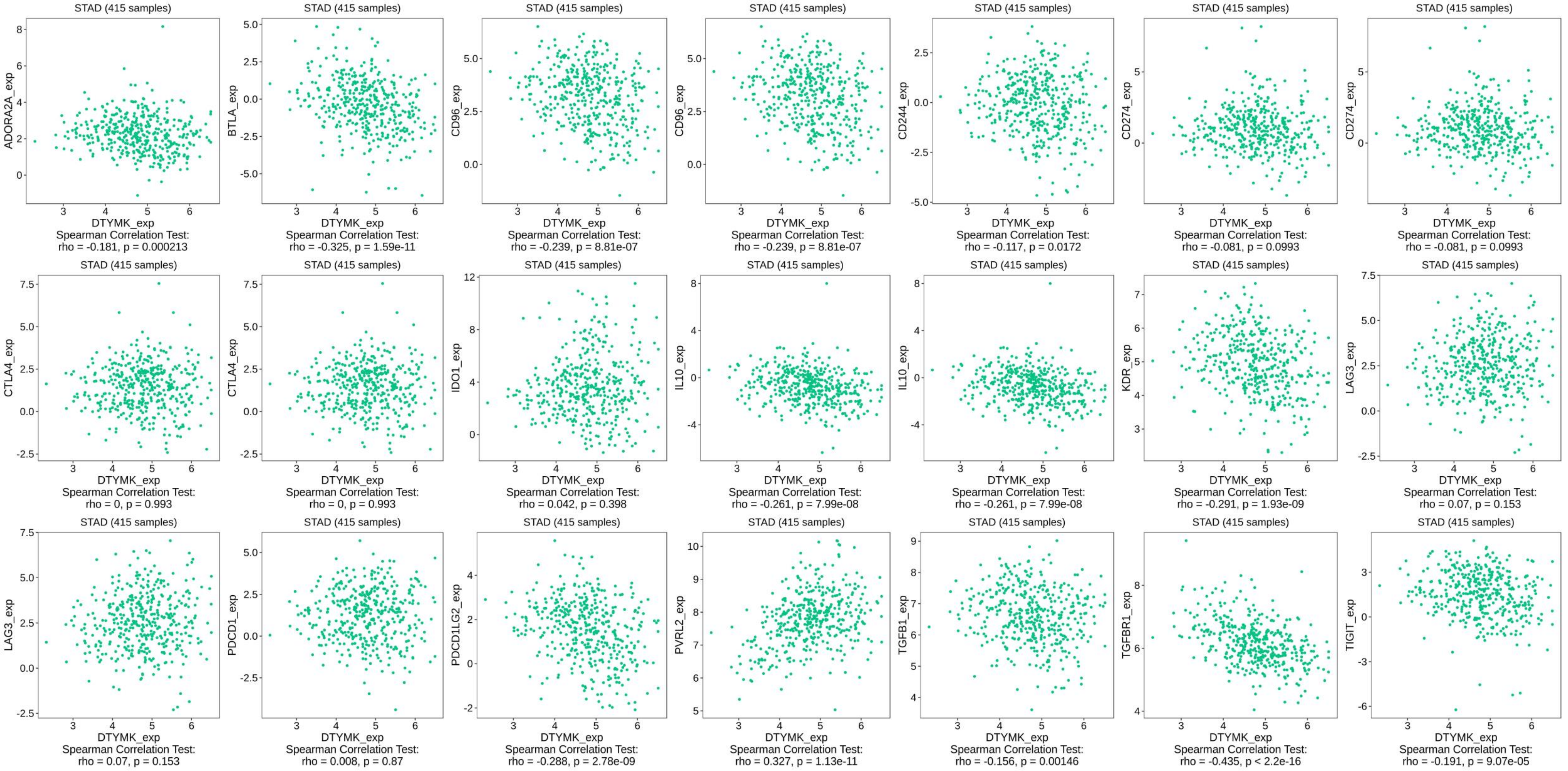

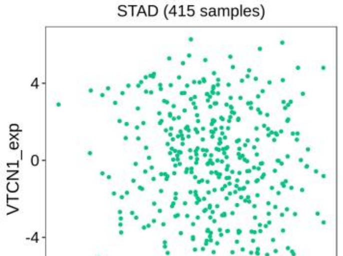

Correlation between DTYMK expression and immunoinhibitors expression in STAD

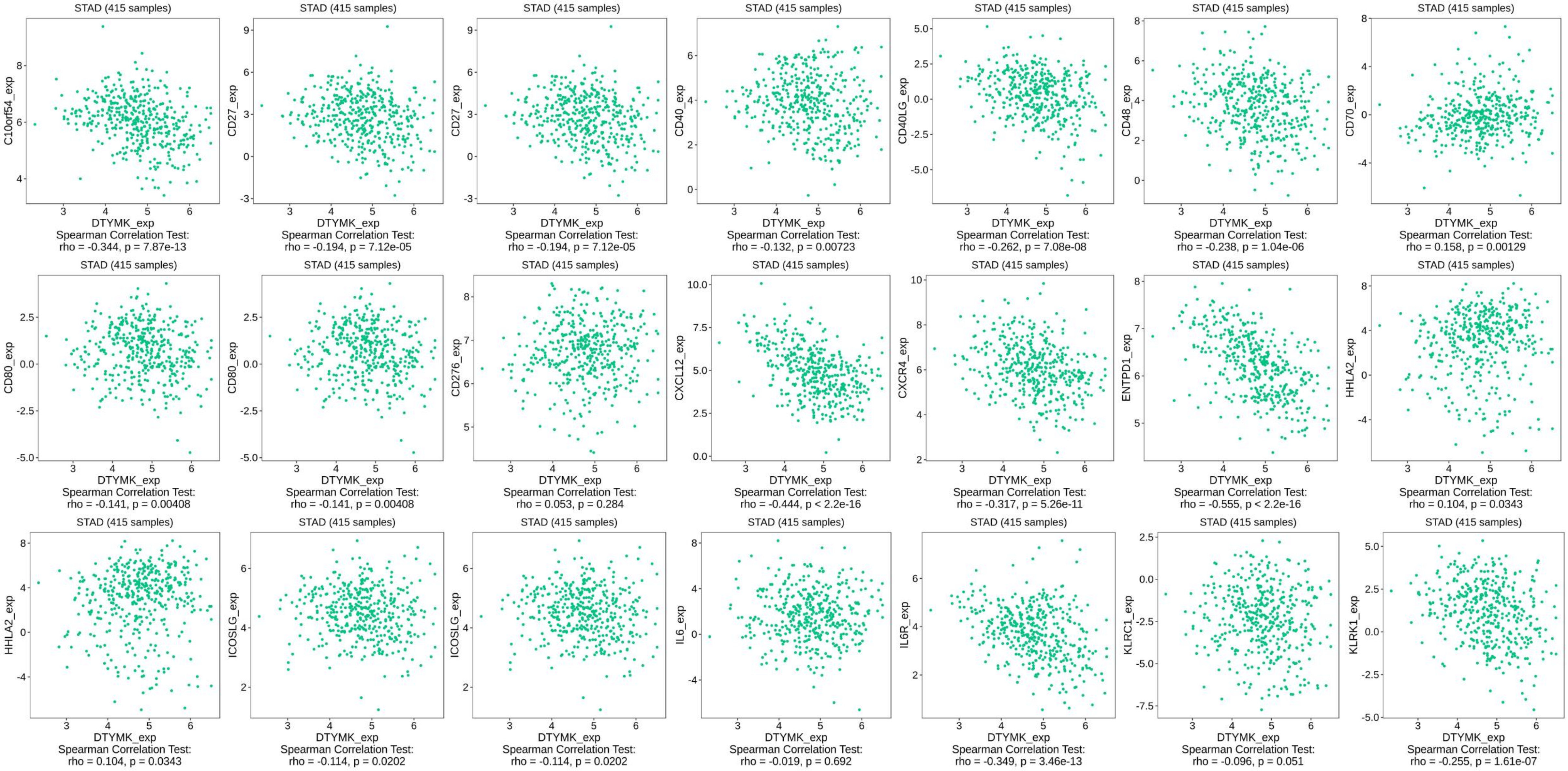

Correlation between DTYMK expression and immunostimulators expression in STAD

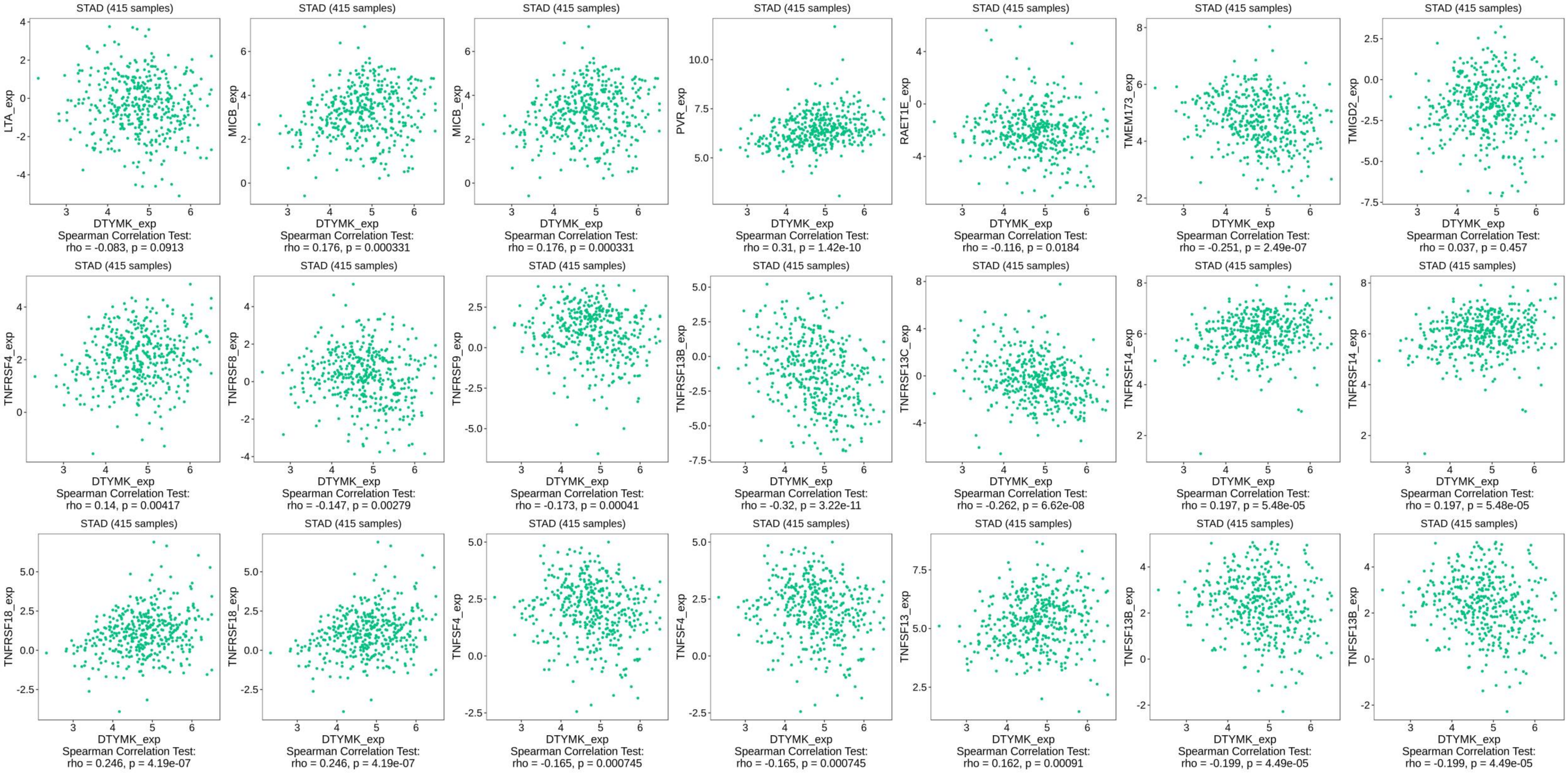

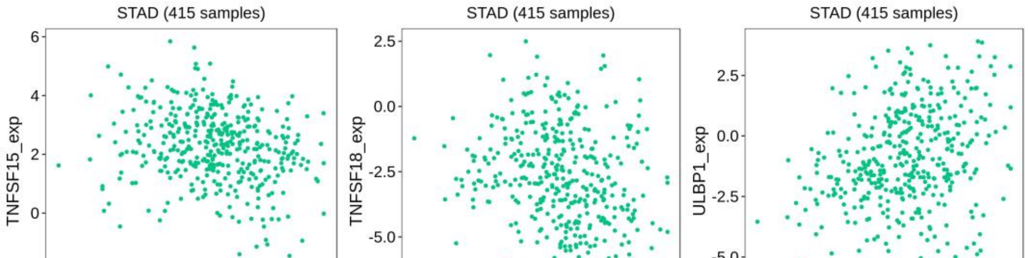

Correlation between DTYMK expression and immunostimulators expression in STAD

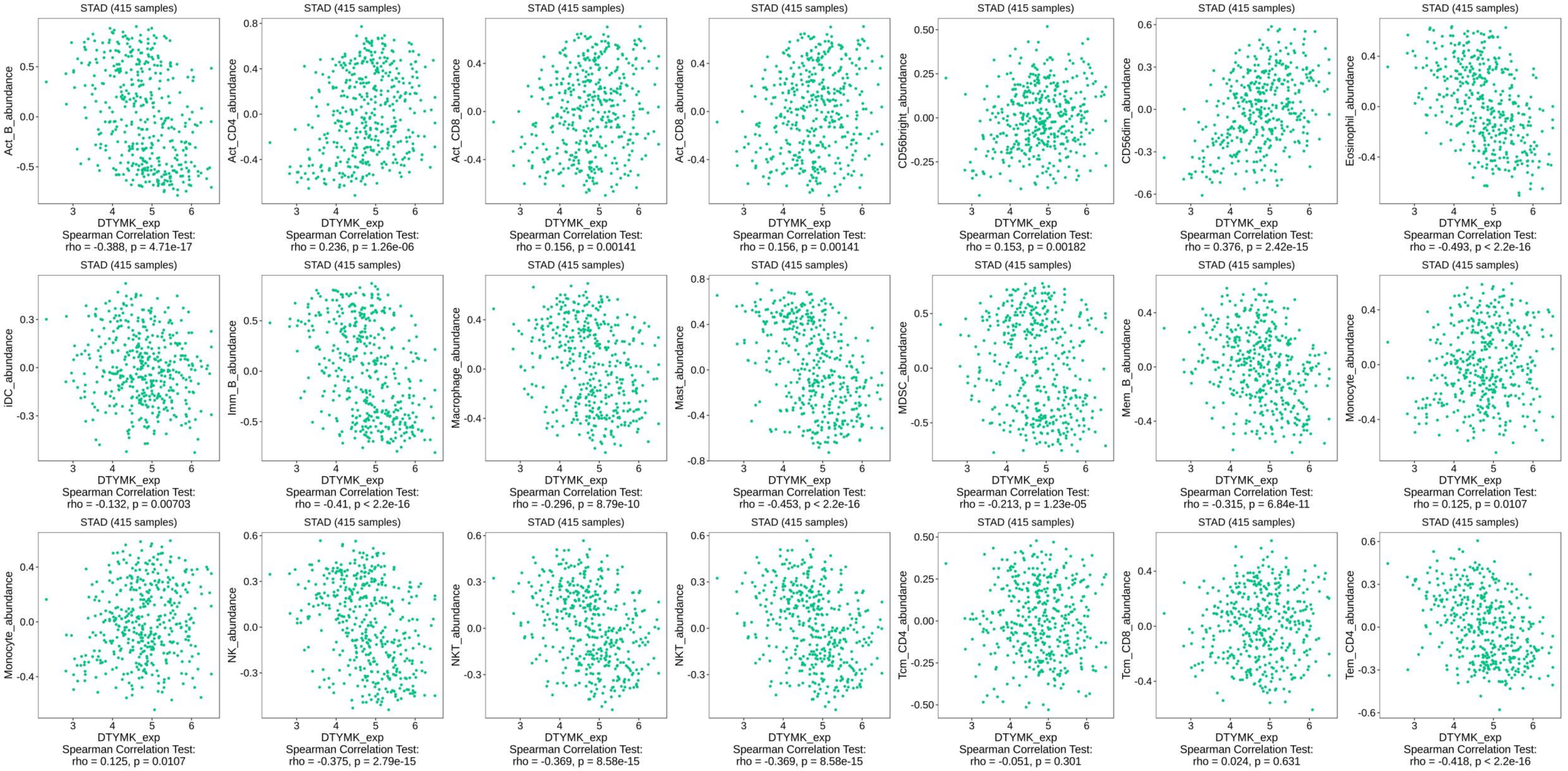

Correlation between DTYMK expression and lymphocytes infiltration in STAD

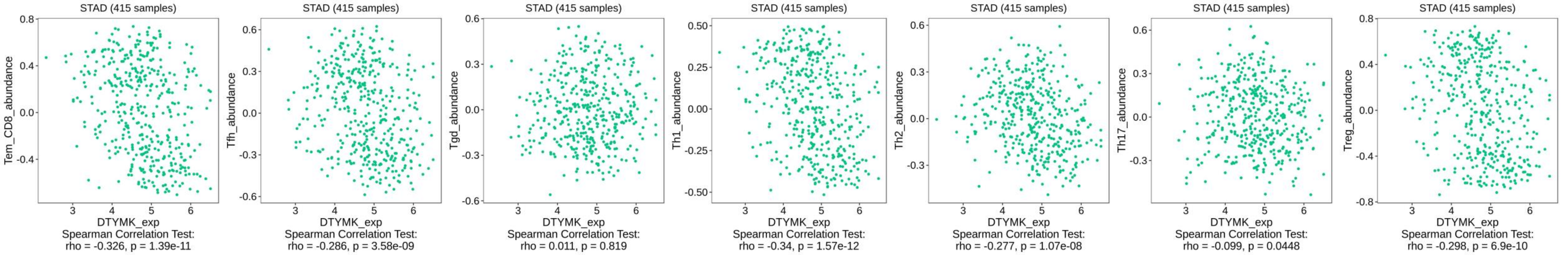

Correlation between DTYMK expression and lymphocytes infiltration in STAD

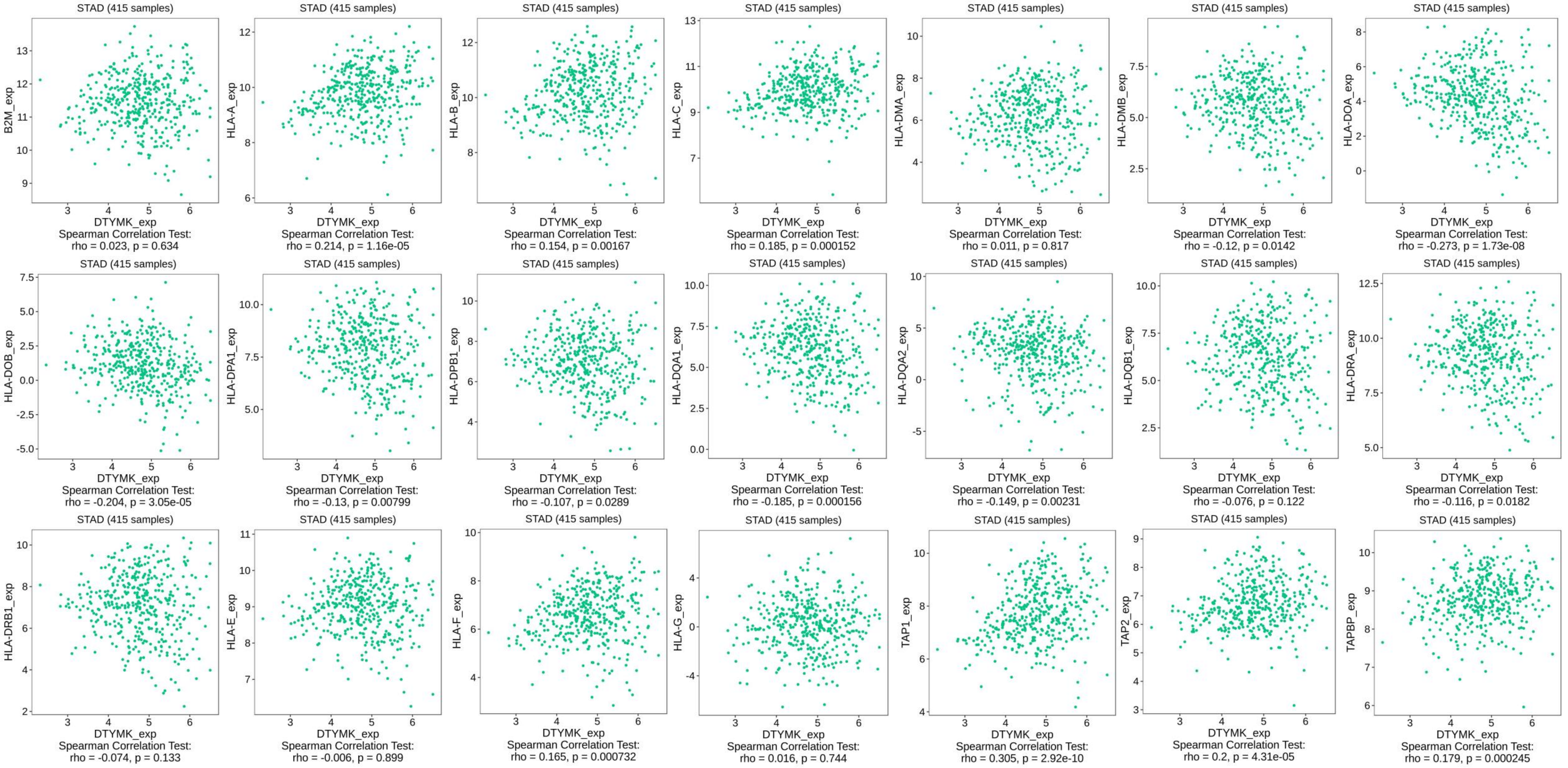

Correlation between DTYMK expression and MHC expression in STAD

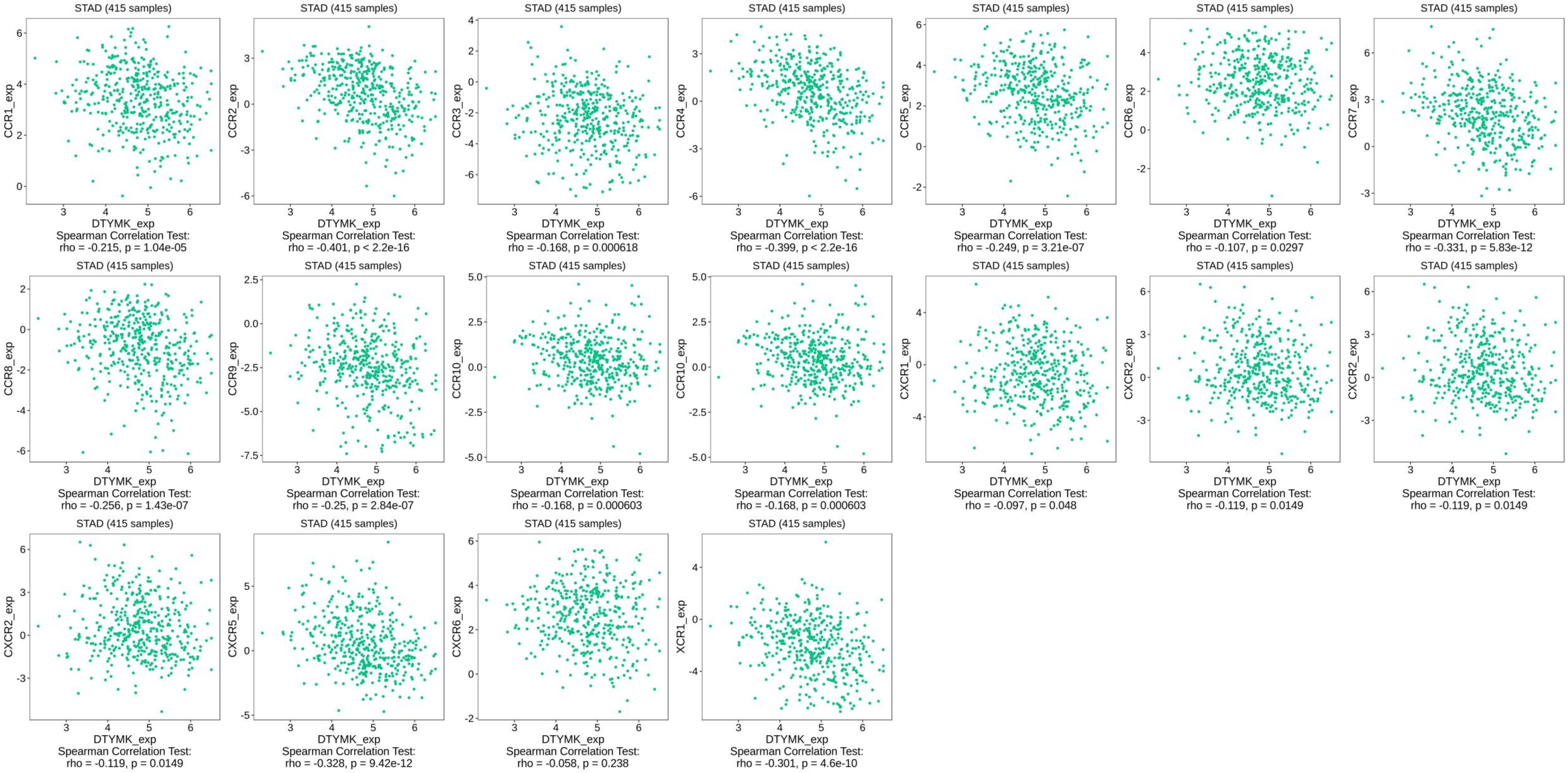

Correlation between DTYMK expression and chemokine receptors expression in STAD
