# Supplementary material for: Deoxythymidylate Kinase as a Promising Marker for Predicting Prognosis and Immune Cell Infiltration of Pan-cancer
Source: Front Mol Biosci. 2022 Jul 12;9:887059. doi: 10.3389/fmolb.2022.887059 (PMC9315941; doi:10.3389/fmolb.2022.887059)
Supplement: Supplementary file 1 [file DataSheet1.zip › supplementary files/supplementary table 1.docx]

Comparison of DTYMK expression between cancers and normal tissues using Oncomine

| Cancer types | Fold change | t value | P value | Reference |
| --- | --- | --- | --- | --- |
| Bladder cancer | 1.751 | 5.424 | 2.09E-7 | Sanchez-Carbayo J Clin Oncol 2006/02/10 |
| Brains and CNS cancer | 2.214 | 8.591 | 5.55E-8 | Bredel  Cancer Res 2005/10/01 |
| Brains and CNS cancer | 1.942 | 6.755 | 3.12E-7 | French  Cancer Res 2006/12/15 |
| Brains and CNS cancer | 2.135 | 6.394 | 7.27E-8 | Sun  Cancer Cell 2006/04/01 |
| Brains and CNS cancer | 2.286 | 10.329 | 1.91E-13 | Sun  Cancer Cell 2006/04/01 |
| Brains and CNS cancer | 1.685 | 6.119 | 6.96E-8 | Sun  Cancer Cell 2006/04/01 |
| Brains and CNS cancer | 4.075 | 14.681 | 1.96E-8 | TCGA |
| Brains and CNS cancer | -2.421 | -11.015 | 3.13E-10 | Lee  Cancer Cell 2006/05/01 |
| Breast cancer | 2.226 | 8.670 | 2.34E-10 | Richardson  Cancer Cell 2006/02/01 |
| Breast cancer | 1.805 | 9.299 | 4.40E-11 | Zhao Mol Biol Cell 2004/06/01 |
| Breast cancer | 1.750 | 8.964 | 2.31E-5 | Perou  Nature 2000/08/17 |
| Breast cancer | 1.714 | 18.832 | 3.64E-42 | TCGA |
| Breast cancer | 1.727 | 8.364 | 1.25E-5 | TCGA |
| Breast cancer | 1.671 | 10.834 | 6.45E-20 | TCGA |
| Breast cancer | 1.516 | 8.471 | 3.53E-12 | TCGA |
| Breast cancer | -2.538 | -13.388 | 1.51E-19 | Finak Nat Med 2008/05/01 |
| Cervical cancer | 2.411 | 5.864 | 2.07E-5 | Zhai Cancer Res 2007/11/01 |
| Cervical cancer | 3.173 | 9.266 | 1.61E-8 | Zhai Cancer Res 2007/11/01 |
| Colorectal cancer | 2.115 | 15.234 | 1.52E-30 | Gaedcke  Genes Chromosomes Cancer 2010/11/01 |
| Colorectal cancer | 3.459 | 10.027 | 3.86E-14 | Sabates-Bellver Mol Cancer Res 2007/12/01 |
| Colorectal cancer | 3.535 | 6.554 | 4.98E-5 | Sabates-Bellver Mol Cancer Res 2007/12/01 |
| Colorectal cancer | 1.898 | 8.573 | 4.97E-11 | TCGA |
| Colorectal cancer | 2.484 | 7.139 | 3.16E-5 | TCGA |
| Colorectal cancer | 1.989 | 11.625 | 9.90E-15 | TCGA |
| Colorectal cancer | 1.928 | 10.467 | 3.62E-14 | TCGA |
| Colorectal cancer | 1.666 | 6.746 | 1.83E-8 | TCGA |
| Colorectal cancer | 2.547 | 11.485 | 1.25E-11 | Hong  Clin Exp Metastasis 2010/02/01 |
| Colorectal cancer | 2.299 | 7.958 | 2.35E-6 | Skrzypczak  PLoS One 2010/10/01 |
| Colorectal cancer | 1.903 | 5.403 | 1.31E-6 | Skrzypczak  PLoS One 2010/10/01 |
| Esophageal cancer | 1.850 | 5.002 | 4.73E-5 | Hu BMC Genomics 2010/10/18 |
| Gastric cancer | 3.140 | 5.963 | 6.80E-7 | DErrico  Eur J Cancer 2009/02/01 |
| Gastric cancer | 3.956 | 6.935 | 6.50E-9 | DErrico  Eur J Cancer 2009/02/01 |
| Head and neck cancer | 1.699 | 5.520 | 1.69E-6 | Cromer  Oncogene 2004/04/01 |
| Head and neck cancer | 1.705 | 6.621 | 5.35E-7 | Sengupta Cancer Res 2006/08/01 |
| Head and neck cancer | 2.070 | 5.479 | 9.13E-7 | Estilo  BMC Cancer 2009/01/12 |
| Head and neck cancer | 2.750 | 6.493 | 9.95E-7 | Ginos  Cancer Res 2004/01/01 |
| Leukemia | 1.841 | 5.477 | 4.26E-6 | Andersson Leukemia 2007/06/01 |
| Leukemia | 2.896 | 6.821 | 8.95E-6 | Andersson Leukemia 2007/06/01 |
| Leukemia | -1.508 | -4.713 | 1.56E-5 | Alizadeh Nature 2000/02/03 |
| Leukemia | -2.199 | -22.961 | 7.66E-41 | Haferlach J Clin Oncol 2010/05/20 |
| Liver cancer | 2.108 | 22.194 | 2.13E-65 | Roessler Cancer Res 2010/12/15 |
| Liver cancer | 1.657 | 5.065 | 1.29E-5 | Cancer Res 2010/12/15 |
| Lung cancer | 2.414 | 14.435 | 3.01E-19 | Hou  PLoS One 2010/04/22 |
| Lung cancer | 2.027 | 9.853 | 8.79E-15 | Hou  PLoS One 2010/04/22 |
| Lung cancer | 3.080 | 7.760 | 9.57E-8 | Hou  PLoS One 2010/04/22 |
| Lung cancer | 1.546 | 11.374 | 7.02E-19 | Landi  PLoS ONE 2008/02/20 |
| Lung cancer | 2.079 | 6.866 | 4.91E-7 | Beer Nat Med 2002/08/01 |
| Lung cancer | 1.533 | 8.354 | 5.52E-11 | Okayama  Cancer Res 2012/01/01 |
| Lymphoma | 3.204 | 6.631 | 5.30E-8 | Basso  Nat Genet 2005/04/01 |
| Lymphoma | 2.984 | 5.499 | 2.12E-5 | Basso  Nat Genet 2005/04/01 |
| Lymphoma | 1.972 | 4.236 | 4.48E-5 | Basso  Nat Genet 2005/04/01 |
| Lymphoma | 3.164 | 7.142 | 3.06E-8 | Brune J Exp Med 2008/09/29 |
| Lymphoma | 4.471 | 8.785 | 3.96E-7 | Brune J Exp Med 2008/09/29 |
| Lymphoma | 2.393 | 5.519 | 2.21E-5 | Brune J Exp Med 2008/09/29 |
| Lymphoma | 2.413 | 4.596 | 5.57E-5 | Brune J Exp Med 2008/09/29 |
| Lymphoma | 1.733 | 4.394 | 2.23E-5 | Alizadeh Nature 2000/02/03 |
| Lymphoma | 1.767 | 4.536 | 1.52E-5 | Alizadeh Nature 2000/02/03 |
| Lymphoma | 5.625 | 10.301 | 1.73E-6 | Eckerle  Leukemia 2009/11/01 |
| Lymphoma | -1.514 | -8.813 | 1.07E-10 | Compagno Nature 2009/06/04 |
| Other cancer | 2.545 | 11.359 | 1.08E-7 | Korkola Cancer Res 2006/01/15 |
| Other cancer | 1.883 | 10.638 | 4.82E-12 | Korkola Cancer Res 2006/01/15 |
| Other cancer | 2.290 | 8.244 | 6.02E-7 | Korkola Cancer Res 2006/01/15 |
| Other cancer | 2.180 | 5.917 | 1.23E-5 | Santegoets  Int J Cancer 2007/08/15 |
| Ovarian cancer | 1.897 | 9.751 | 9.47E-9 | Bonome  Cancer Res 2008/07/01 |
| Ovarian cancer | 1.625 | 8.464 | 7.18E-6 | TCGA |
| Ovarian cancer | 1.545 | 4.563 | 1.78E-5 | Yoshihara  Cancer Sci 2009/08/01 |
| Pancreatic cancer | 1.703 | 4.977 | 8.62E-6 | Pei Cancer Cell 2009/09/08 |
| Prostate cancer | 2.110 | 4.258 | 2.66E-5 | Singh Cancer Cell 2002/03/01 |
| Sarcoma | 1.517 | 9.671 | 4.93E-13 | Barretina  Nat Genet 2010/07/04 |
| Sarcoma | 1.536 | 8.492 | 2.88E-9 | Barretina  Nat Genet 2010/07/04 |
| Sarcoma | 1.561 | 6.796 | 7.00E-8 | Barretina  Nat Genet 2010/07/04 |
| Sarcoma | 1.594 | 6.785 | 3.21E-8 | Barretina  Nat Genet 2010/07/04 |
