# Supplementary figures and images for: Deoxythymidylate Kinase as a Promising Marker for Predicting Prognosis and Immune Cell Infiltration of Pan-cancer
Source: Front Mol Biosci. 2022 Jul 12;9:887059. doi: 10.3389/fmolb.2022.887059 (PMC9315941; doi:10.3389/fmolb.2022.887059)

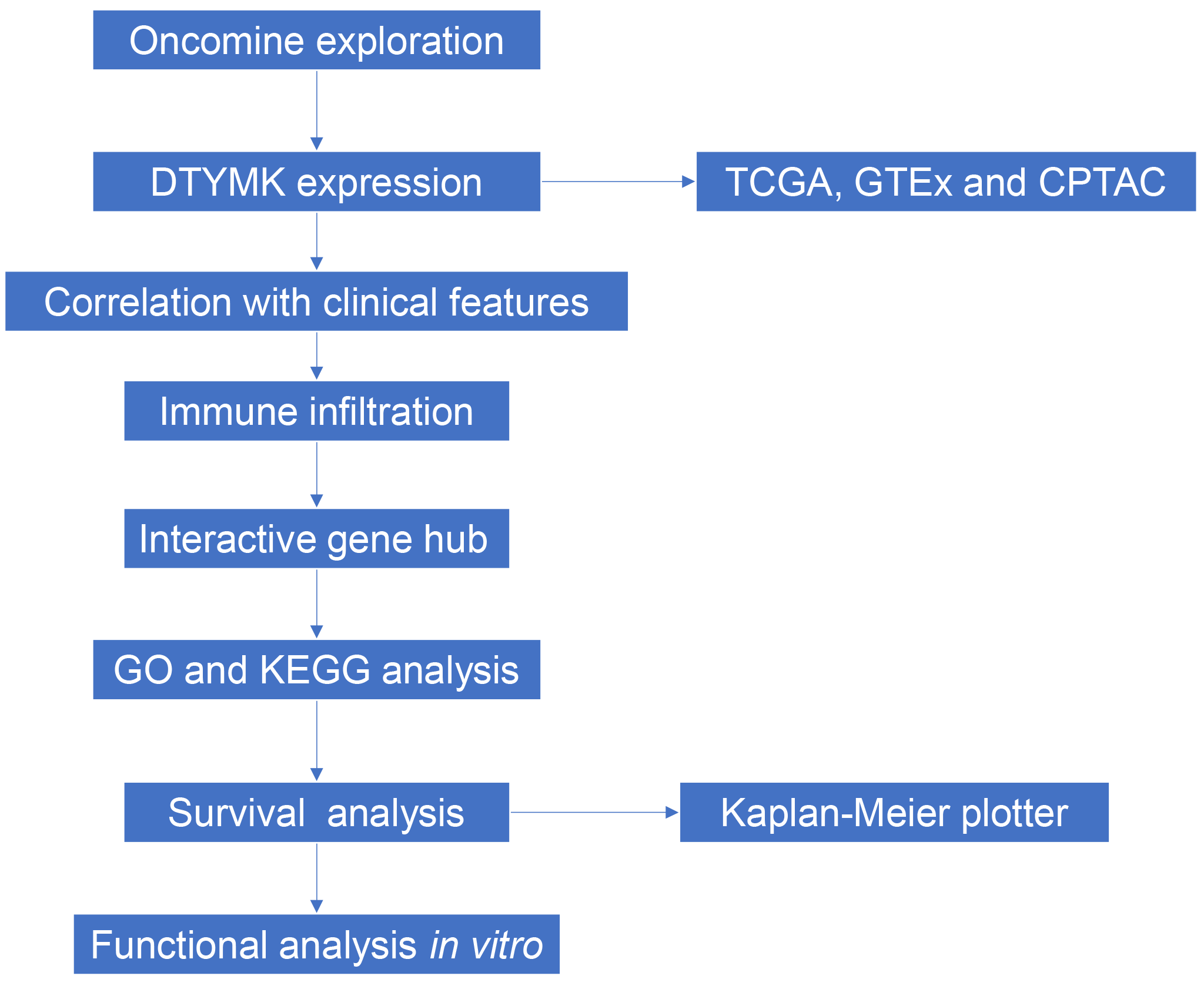

Supplement: Supplementary file 1 [file DataSheet1.zip › supplementary files/supplementary figure 1.tif]

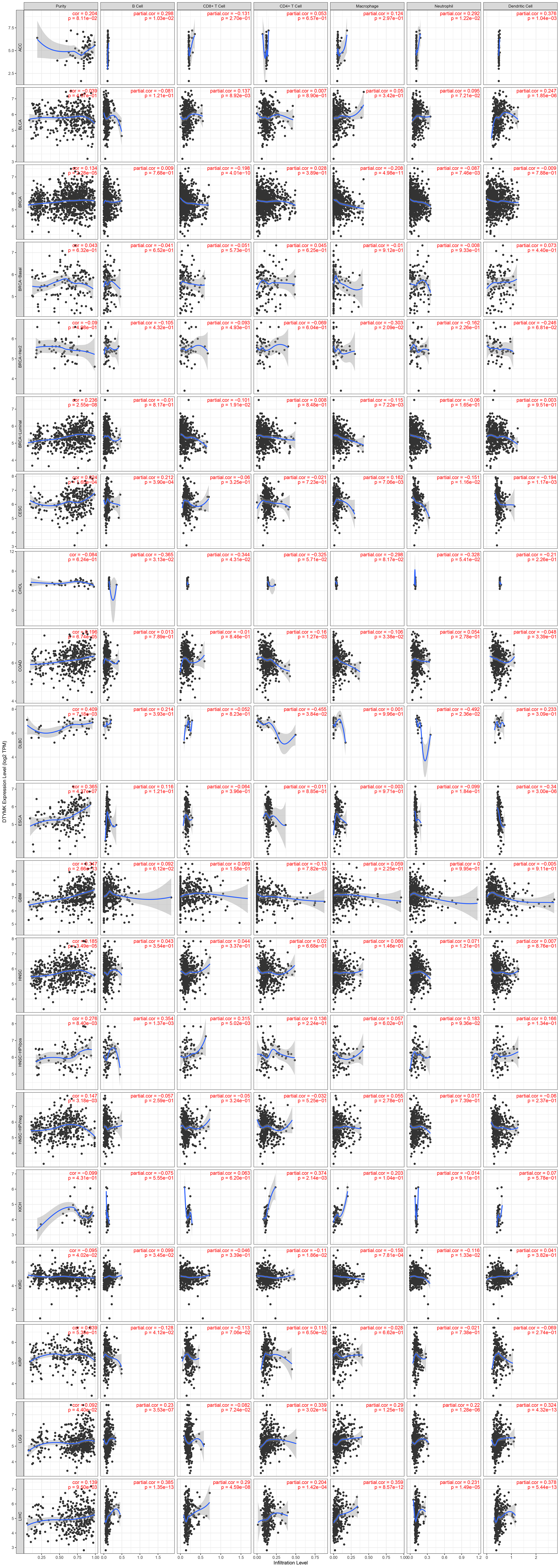

Supplement: Supplementary file 1 [file DataSheet1.zip › supplementary files/supplementary figure 2a.tif]

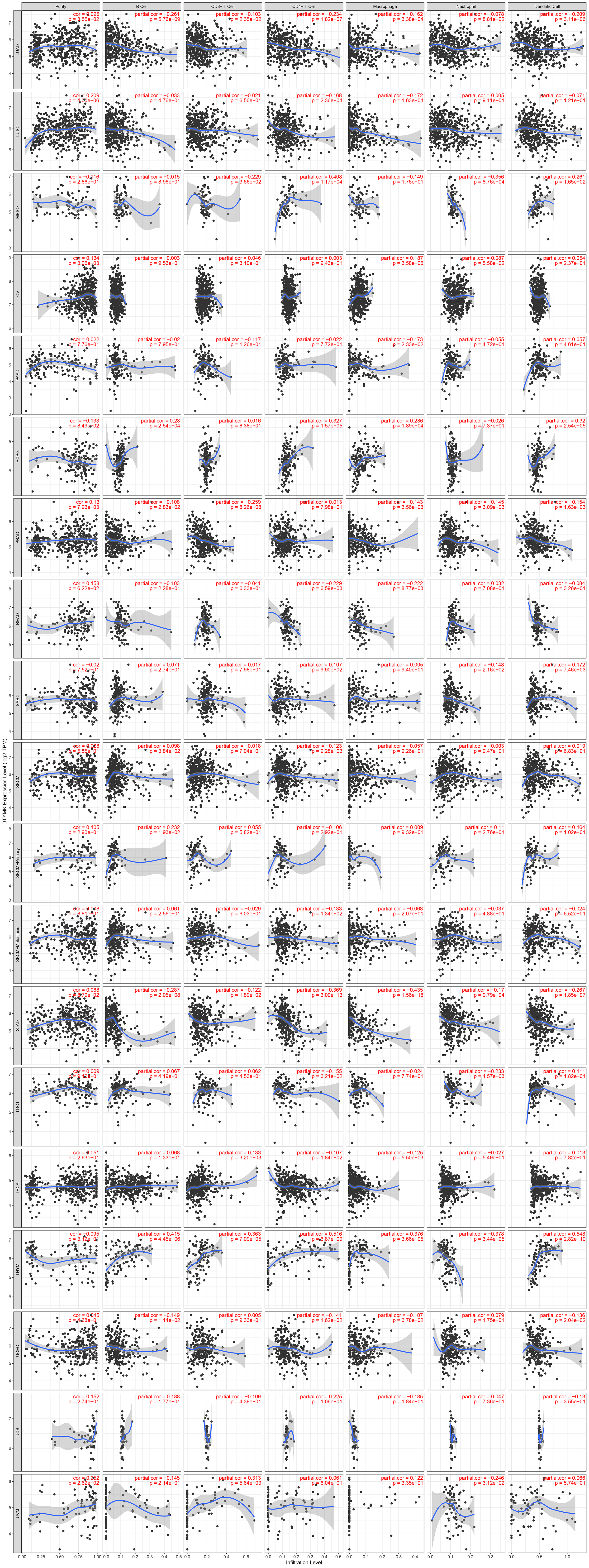

Supplement: Supplementary file 1 [file DataSheet1.zip › supplementary files/supplementary figure 2b.tif]
